# Engineered virus-like particles for transient delivery of prime editor ribonucleoprotein complexes in vivo

**DOI:** 10.1038/s41587-023-02078-y

**Published:** 2024-01-08

**Authors:** Meirui An, Aditya Raguram, Samuel W. Du, Samagya Banskota, Jessie R. Davis, Gregory A. Newby, Paul Z. Chen, Krzysztof Palczewski, David R. Liu

**Affiliations:** 1 Merkin Institute of Transformative Technologies in Healthcare, Broad Institute of MIT and Harvard, Cambridge, MA USA; 2 Department of Chemistry and Chemical Biology, Harvard University, Cambridge, MA USA; 3 Howard Hughes Medical Institute, Harvard University, Cambridge, MA USA; 4 Gavin Herbert Eye Institute, Center for Translational Vision Research, Department of Ophthalmology, University of California, Irvine, CA USA; 5 Department of Physiology and Biophysics, University of California, Irvine, CA USA; 6 Department of Chemistry, University of California, Irvine, CA USA; 7 Department of Molecular Biology and Biochemistry, University of California, Irvine, CA USA

**Keywords:** Genetic engineering, Protein delivery

## Abstract

Prime editing enables precise installation of genomic substitutions, insertions and deletions in living systems. Efficient in vitro and in vivo delivery of prime editing components, however, remains a challenge. Here we report prime editor engineered virus-like particles (PE-eVLPs) that deliver prime editor proteins, prime editing guide RNAs and nicking single guide RNAs as transient ribonucleoprotein complexes. We systematically engineered v3 and v3b PE-eVLPs with 65- to 170-fold higher editing efficiency in human cells compared to a PE-eVLP construct based on our previously reported base editor eVLP architecture. In two mouse models of genetic blindness, single injections of v3 PE-eVLPs resulted in therapeutically relevant levels of prime editing in the retina, protein expression restoration and partial visual function rescue. Optimized PE-eVLPs support transient in vivo delivery of prime editor ribonucleoproteins, enhancing the potential safety of prime editing by reducing off-target editing and obviating the possibility of oncogenic transgene integration.

## Abstract (web summary)

Delivery of prime editors in vivo is improved using virus-like particles.

## Main

Among current genome editing systems that function in both dividing and nondividing mammalian cells in vitro and in vivo, prime editing^1^ offers unusual versatility by enabling the replacement of a target DNA sequence with virtually any other specified sequence containing up to several hundred inserted, deleted or substituted base pairs^2–11^. This versatility makes PE systems particularly promising for the treatment of a broad range of genetic diseases in humans. A prime editor (PE) is an engineered protein consisting of a catalytically impaired programmable nickase domain (such as a Cas9 nickase) fused to an engineered reverse transcriptase (RT) domain. The prime editing guide RNA (pegRNA) specifies the target protospacer sequence and simultaneously encodes the desired edits in the reverse transcription template in the 3′ extension of the pegRNA. The mechanism of prime editing requires three independent nucleic acid hybridization events before editing can take place and does not rely on double-strand DNA breaks or donor DNA templates. As a result of this mechanism, prime editing is inherently resistant to off-target editing or bystander editing, and can proceed with few indel byproducts or other undesired consequences of double-strand DNA breaks^1,12–21^.

Fully realizing the potential of prime editing for research or therapeutic applications in mammals requires safe and efficient methods capable of delivering PEs into tissues in vivo. So far, several groups have reported the in vivo delivery of PE via viral delivery methods, including adenoviruses^8^ and adeno-associated viruses (AAV)^8–12,22–25^. Viral delivery methods, however, require that the transgene be encoded directly in the viral gene expression cassette, limiting transgene size. The AAV genome has a cargo gene size limitation of ~4.7 kb (not including inverted terminal repeats)^26^, requiring large cargoes such as PEs (6.4 kb in gene size for a first-generation PE) to be split into multiple AAVs^25^, limiting editing efficiency especially at moderate or low vector doses^27^. Viral delivery methods also pose potential safety risks including increased off-target editing from sustained transgene expression^28^ and the possibility of unwanted cargo DNA integration into host cell genomes^29^. Nonviral delivery methods, such as lipid nanoparticles, avoid some of these issues by packaging editors as transiently expressing messenger RNAs (mRNAs). In vivo nonviral targeting of tissues beyond the liver for efficient therapeutic gene editing remains a challenge^30,31^, however, despite recent advances targeting hematopoietic stem cells^32^.

Virus-like particles (VLPs) are potentially promising delivery vehicles that in principle offer key benefits of both viral and nonviral delivery methods^33^. VLPs are formed by spontaneous assembly and budding of retroviral polyproteins that encapsulate cargo molecules from producer cells. VLPs lack a packaged genome but retain the ability to transduce mammalian cells and release cargo^34,35^. Previous studies explored VLPs for delivering Cas9 nuclease^36–43^. We recently reported efficient in vivo delivery of adenine base editor (ABE):single guide RNA (sgRNA) ribonucleoproteins (RNPs) with iteratively engineered virus-like particles (eVLPs)^44^ that overcame specific molecular bottlenecks in cargo packaging, release and localization.

Engineered VLPs offer several advantages over other delivery methods as a candidate for in vivo PE delivery. First, eVLPs are not subject to stringent cargo size limitations, obviating the requirement of splitting PEs into multiple separate vectors. In addition, eVLPs can package RNPs, the most transient form of gene editing agents, thereby reducing frequency of off-target editing by minimizing the exposure duration of the genome to editing agents^44–46^. Since eVLPs lack DNA^34,44^, they avoid unwanted integration of viral genetic material into the genomes of transduced cells. Finally, eVLPs can be pseudotyped with different glycoproteins, enabling specific targeting of cell types of interest^42^ with envelope protein engineering efforts.

In this Article, we report the development of a PE-eVLP system that delivers complete PE systems including pegRNAs and nicking sgRNAs (ngRNAs) as RNPs. Simple replacement of base editors (BEs) with PEs in the optimized BE-eVLP system yielded very low functional delivery of prime editing systems (<1% editing efficiency in cultured mammalian cells). Through systematic identification of PE-eVLP delivery bottlenecks and engineering corresponding solutions, we developed third-generation v3 PE-eVLPs that offer a 79-fold improvement in prime editing efficiency compared to v1 PE-eVLPs in mouse Neuro-2A (N2A) cells and a 170-fold improvement in human HEK293T cells. A single subretinal injection of v3 PE-eVLPs demonstrated efficient in vivo prime editing in mouse models, correcting a 4-bp deletion in *Mfrp* in the *rd6* mouse model of retinal degeneration (15% average efficiency) and correcting an *Rpe65* substitution to partially rescue visual function in the *rd12* model (7.2% average efficiency). Our study establishes PE-eVLPs as a virus-free method for the in vivo delivery of prime editing systems in RNP form.

## Results

### Delivery of PE:pegRNA RNPs via eVLPs in cultured cells

**Fig. 1:**
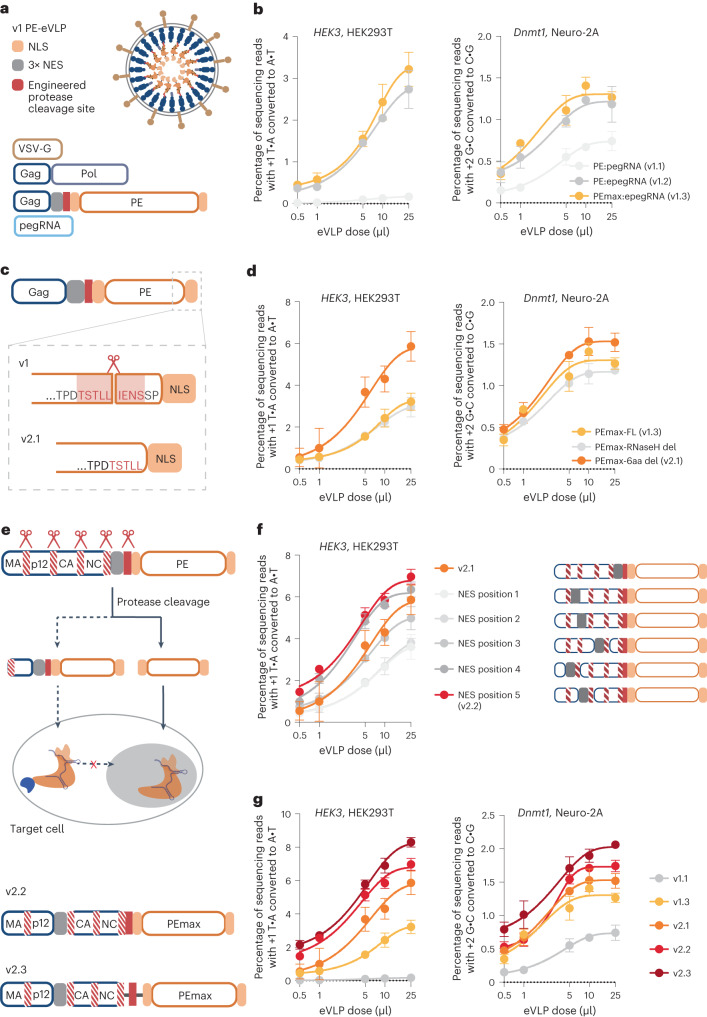
Engineering of PE and eVLP architectures. **a**, Schematic of v1 PE-eVLPs. **b**, Prime editing efficiencies of v1 PE-eVLPs for the *HEK3* +1 T-to-A edit in HEK293T cells and *Dnmt1* +2 G-to-C edit in N2A cells. Adoption of epegRNAs (v1.2) and the PEmax architecture (v1.3) improved prime editing efficiencies compared to v1.1 PE-eVLPs. **c**, Schematic of PE engineering to eliminate the endogenous protease cleavage site (TSTLLIENS) in MMLV RT. **d**, Representative improvements in PE-eVLP editing efficiencies as a result of RT domain engineering. PEmax–FL denotes v1 PE-eVLPs with full-length MMLV RT as PE effector domain; PEmax–RNaseH del denotes v1 PE-eVLPs with RNaseH domain-truncated RT used as the PE effector domain; PEmax-6aa del denotes v2.1 PE-eVLPs with six-amino-acid-deleted RTs used as the PE effector domain. **e**, Schematic of the proposed mechanism of eVLP maturation and cargo delivery, and the design of v2.2 and v2.3 PE-eVLPs. **f**, Prime editing efficiencies of PE-eVLPs with the 3× NES placed at various locations (NES position 1–5) of the Gag domain. NES position 5 (v2.2) showed improved editing compared to that of v2.1 PE-eVLPs. **g**, Comparison of prime editing efficiencies with v1, v2.1, v2.2 and v2.3 PE-eVLPs at the *HEK3* locus in HEK293T cells and *Dnmt1* locus in N2A cells. Values shown in all graphs represent the mean prime editing efficiencies ± s.e.m. of three biological replicates. Data were fitted to four-parameter logistic curves using nonlinear regression. Comparisons of different versions of PE-eVLPs are made with eVLPs produced and transduced in parallel in one large experiment to minimize variability between preparations. Data from all PE-eVLPs produced and tested in parallel are provided in Supplementary Fig 1. The graphs in **b**, **d**, **f** and **g** show a subset of data from Supplementary Fig 1. For all conditions, 30,000–35,000 cells were treated with eVLPs containing ~2.5 × 10^8^ eVLPs μl^−1^.

Previously, we engineered a v4 BE-eVLP architecture that package and deliver adenine BEs^44^. To test if the v4 BE-eVLP architecture can package and deliver PEs (Fig. 1a), we produced PE-eVLPs by transfecting Gesicle 293T cells with plasmids encoding the envelope protein VSV-G, wild-type MMLV Gag–Pol polyprotein, engineered MMLV Gag–PE fusion protein and pegRNA. We then treated target cells with increasing doses of eVLPs. Very low levels of prime editing resulted from this v1.1 PE-eVLP system when programmed to install a +1 T-to-A edit at HEK293T site 3 (hereafter named *HEK3*) in HEK293T cells (0.17% editing efficiency) and a +2 G-to-C edit at *Dnmt1* in N2A cells (0.74% editing efficiency) at the highest dose of eVLPs tested (25 μl, approximately 6.3 × 10^9^ eVLP particles for a well containing 30,000–35,000 cells) (Fig. 1b). We then incorporated two modifications that we recently reported to enhance prime editing. First, we developed v1.2 PE-eVLPs that use engineered pegRNAs (epegRNAs)^4^, which have a pseudoknot motif at their 3′ end that protects the pegRNA from degradation. Second, we replaced the PE protein with PEmax^2^, an improved PE protein architecture that include SpCas9 mutations, an optimized linker between the Cas9 nickase and RT domain, codon optimization, and nuclear localization signal (NLS) optimization. The resulting v1.3 PE-eVLPs showed 19-fold and 1.7-fold improvement in prime editing efficiency at *HEK3* (3.2%) and *Dnmt1* (1.3%), respectively, compared to v1.1 PE-eVLPs (Fig. 1b).

### Systematic engineering of PE and eVLP architecture

To further enhance the efficiency of PE-eVLPs, we sought to identify mechanistic bottlenecks in v1 PE-eVLPs. The formation of eVLPs relies on the proteolytic processing of the Gag–Pol polyprotein into multiple functional domains by MMLV protease. Coincidentally, the RT domain of canonical PEs also originates from MMLV, and therefore contains an endogenous TSTLLIENS protease cleavage site at the C-terminus^47^ (Fig. 1c). We hypothesized that cleavage at this site may eliminate the crucial C-terminal NLS that direct nuclear localization of the PEs. Therefore, we deleted six amino acids at the C-terminus of MMLV RT to remove the protease recognition site, yielding v2.1 PE-eVLPs. This modification offered consistent improvement in PE-eVLP editing efficiency over v1.3 PE-eVLPs (averaging 1.8-fold and 1.2-fold, respectively) at both the *HEK3* locus and *Dnmt1* locus (Fig. 1d). We tested further truncation of the MMLV RT to remove the entire RNaseH domain, a common truncation strategy used by AAV systems to reduce PE size and facilitate dual-AAV packaging^8–12,22–25^. In the PE-eVLP system, however, eliminating the RNaseH domain resulted in consistently lower editing efficiencies in both HEK293T and N2A cells than v2.1 PE-eVLP (Fig. 1d).

The nuclear export signal (NES) promotes cytoplasmic localization of Gag–cargo fusion protein. After protease cleavage, however, gene editing agents must be separated from the NES for efficient nuclear localization. The presence of four additional MMLV protease cleavage sites within the Gag polyprotein raises the possibility of incomplete cleavage, resulting in a fraction of PE cargo retaining some portion of Gag along with NESs (Fig. 1e). Indeed, we previously observed cargo of varying sizes in BE-eVLPs by western blot^44^, supporting the hypothesis that improperly cleaved products can be packaged in eVLPs. To mitigate this problem, we relocated the NESs within the Gag polyprotein (Fig. 1f). We inserted 3× NES before the protease cleavage site of each Gag subdomain and two additional sites that had been previously shown to tolerate large insertions in the MMLV Gag–Pol^48^. Among five tested sites, inserting 3× NES between the p12 and CA domains of Gag (NES position 5) supported the highest prime editing efficiency in HEK293T cells, yielding v2.2 PE-eVLP (Fig. 1f).

Lastly, to facilitate cargo release, we inserted GGS linkers flanking the engineered protease cleavage site to increase the accessibility of the site for protease processing. The resulting v2.3 PE-eVLP architecture (Fig. 1e) containing MMLV RT truncation, NES optimization and linker optimization showed an average 2.6-fold and 1.6-fold improvement in prime editing efficiency over the v1.3 PE-eVLP architecture at the highest dose tested at the *HEK3* locus in HEK293T cells and *Dnmt1* locus in N2A cells, respectively (Fig. 1g).

### Dependence of PE-eVLP performance on edit type

We previously demonstrated that prime editing efficiencies can be impaired by cellular mismatch repair (MMR) pathways, and that evasion or inhibition of MMR improves prime editing efficiencies^2^. Given the transient nature of PE:pegRNA RNPs packaged in eVLPs, we hypothesized that the reversion of the installed edit by MMR might be especially detrimental to editing efficiency.

**Extended Data Fig. 1:**
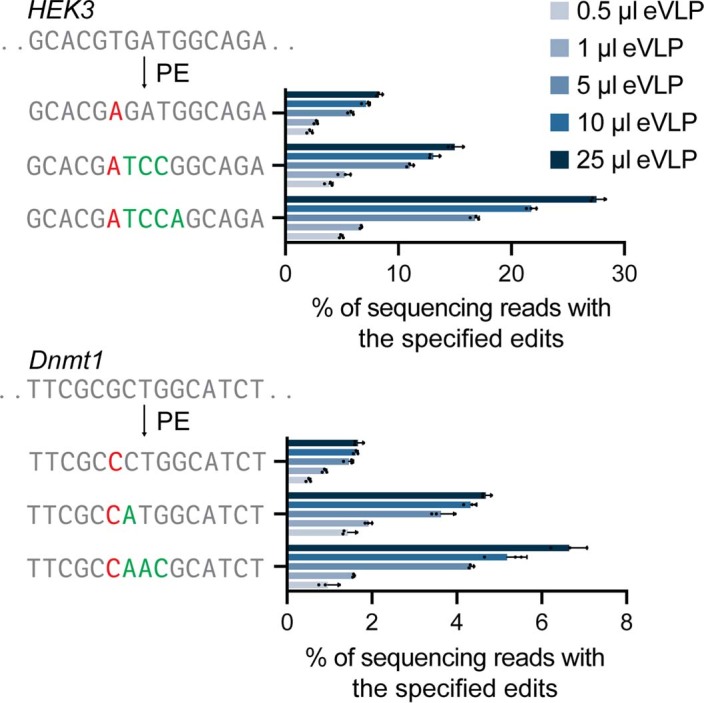
MMR-evading edits support more efficient prime editing. Installation of nearby mutations improves prime editing efficiencies of v2.3 PE-eVLPs at the *HEK3* locus and *Dnmt1* locus in HEK293T and N2A cells respectively. Values shown in all graphs represent the average prime editing efficiency of three biological replicates and error bars represent the standard deviation. Data were fitted to four-parameter logistic curves using nonlinear regression.

We previously discovered that the installation of additional silent or benign mutations near the target edit causes the prime editing intermediate to natively evade MMR, enhancing editing efficiencies and product purities^2^. To test this possibility for eVLP-delivered prime editing systems, we installed additional nearby substitutions at the *HEK3* locus and *Dnmt1* locus with the v2.3 PE-eVLP system. In both cases, prime editing efficiencies were substantially improved, achieving 28% average editing for a 5-bp substitution edit at *HEK3* and 6.6% editing for a 4-bp substitution edit at *Dnmt1* at the highest dose tested (Extended Data Fig. 1). Due to codon redundancy, many therapeutically relevant edits can be achieved with the concurrent installation of additional silent mutations to evade MMR^49,50^. The substantial benefit of MMR evasion to PE-eVLP editing outcomes informs the design of optimal epegRNAs when using PE-eVLPs.

### Insufficient epegRNA packaging limits PE-eVLP efficiency

**Fig. 2:**
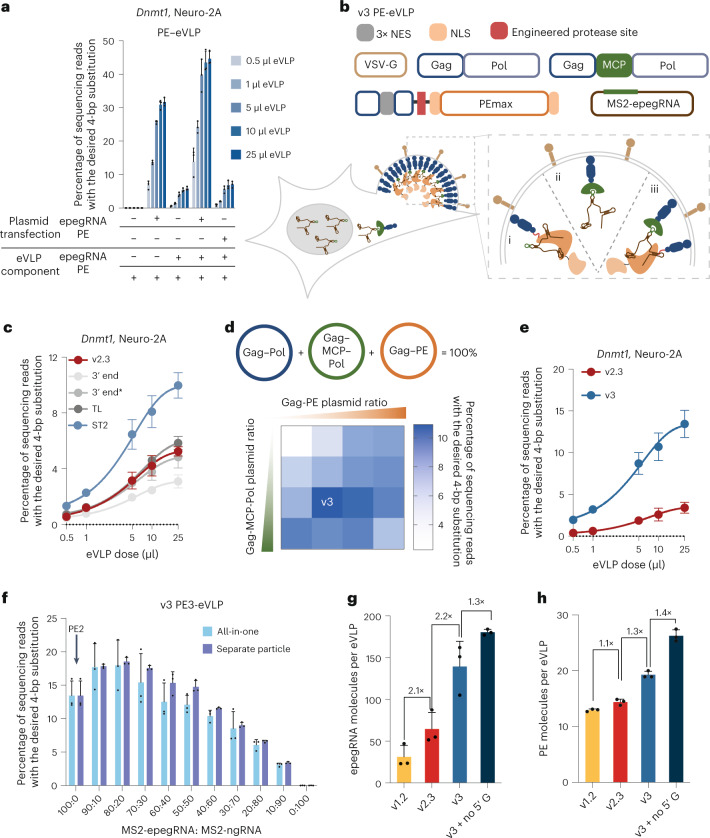
Engineering of pegRNA and ngRNA. **a**, A dual transfection/transduction experiment identifies epegRNAs as the limiting component in the v2.3 PE-eVLPs. **b**, Schematic of v3 PE-eVLPs utilizing the MCP–MS2 strategy for the recruitment of epegRNAs. The incorporation of MCP–MS2 strategy enables three modes of guide RNA loading into eVLPs: (i) via binding to PE, (ii) via MS2 stem–loop binding to MCP and (iii) via MCP:MS2-gRNAs:PE three-component interaction. **c**, Editing efficiencies of v2.3 PE-eVLPs at the *Dnmt1* locus in N2A cells with MS2 stem–loop insertion at various locations in epegRNAs. The 3′ end denotes v2.3 PE-eVLPs with insertion of the MS2 stem–loop after the structured tevoPreQ1 motif of the epegRNA; 3′ end* denotes v2.3 PE-eVLPs with insertion of the MS2 stem–loop directly after the 3′ extension of the pegRNA, thereby using the MS2 stem–loop to mimic a structured motif at the 3′ end of epegRNAs; TL denotes v2.3 PE-eVLPs with insertion of the MS2 stem–loop within the tetraloop of the pegRNA scaffold; and ST2 denotes v2.3 PE-eVLPs with insertion of the MS2 stem–loop within the ST2 loop of the pegRNA scaffold. **d**, Heatmap of editing efficiencies from stoichiometry optimization of Gag–Pol, Gag–MCP–Pol and Gag–PE plasmids for production of v3 PE-eVLPs. **e**, Comparison of editing efficiencies with v2.3 and v3 PE-eVLPs at the *Dnmt1* locus in N2A cells. **f**, Editing efficiencies of all-in-one and separate-particle v3 PE3-eVLP systems with varying MS2-epegRNA to MS2-ngRNA ratios. **g**, Quantification of the number of epegRNAs molecules packaged per eVLP in successive generations of PE-eVLPs. **h**, Quantification of the number of PE protein molecules packaged per eVLP in successive generations of PE-eVLPs. Values represent the mean prime editing efficiencies ± s.e.m. of three biological replicates (**a** and **c**–**f**) or three technical replicates (**g** and **h**). Data were fitted to four-parameter logistic curves using nonlinear regression. Data from all PE-eVLPs produced and tested in parallel are provided in Supplementary Fig 2. The graphs in **c**, **d** and **e** show a subset of data from Supplementary Fig 2. For all conditions, 30,000–35,000 cells were treated with eVLPs containing ~2.5 × 10^8^ eVLPs μl^−1^.

**Extended Data Fig. 2:**
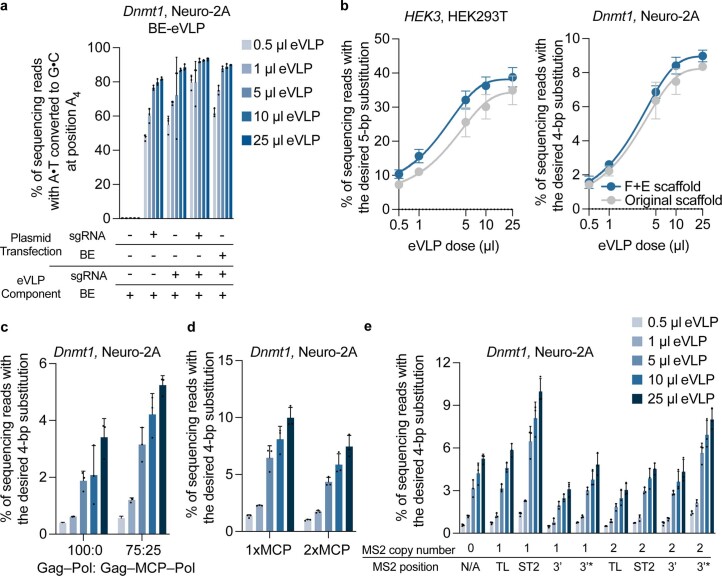
Optimization of eVLP cargo loading and delivery. **a**, A dual transfection/transduction experiment with base editor-delivering BE-eVLPs demonstrates that supplementation of sgRNA does not improve BE-eVLP editing efficiency. **b**, Adopting the flip-and-extend (F+E) guide RNA scaffold in epegRNAs modestly improves editing efficiencies of v2.3 PE-eVLPs at *HEK3* and *Dnmt1* in HEK293T and N2A cells respectively. **c**, Comparison of v2.3 PE-eVLP editing efficiencies at the *Dnmt1* locus in N2A cells with Gag–Pol, or a 3:1 ratio of Gag–Pol:Gag–MCP–Pol. **d**, Comparison of v2.3 PE-eVLPs with one copy or two copies of MCP fused to Gag–Pol. **e**, Editing efficiencies of v2.3 PE-eVLPs at the *Dnmt1* locus in N2A cells with MS2 stem–loop insertions in epegRNA. Zero, one, or two copies of MS2 stem–loop were inserted at various locations of epegRNAs. The position of the MS2 stem–loop insertion is as follows: 3’ denotes v2.3 PE-eVLPs with insertion of the MS2 stem–loop after the structured tevoPreQ1 motif of the epegRNA; 3’* denotes v2.3 PE-eVLPs with insertion of the MS2 stem–loop directly after the 3’-extension of the pegRNA, thereby using the MS2 stem–loop to mimic a structured motif at the 3’ end of the epegRNAs; TL denotes v2.3 PE-eVLPs with insertion of the MS2 stem–loop within the tetraloop of the pegRNA scaffold; ST2 denotes v2.3 PE-eVLPs with insertion of the MS2 stem–loop within the ST2 loop of the pegRNA scaffold. Values shown in all graphs represent the average base editing efficiency (a) or prime editing efficiency (b-e) of three biological replicates and error bars represent the standard deviation. Data were fitted to four-parameter logistic curves using nonlinear regression.

In contrast to the mechanism of PE protein packaging, which depends on fusion with the Gag polyprotein, epegRNA packaging relies entirely on noncovalent association with the Cas9 domain of PEs. To test if epegRNA delivery is a bottleneck in PE-eVLP performance, we supplemented epegRNAs via transfection of plasmids encoding epegRNAs 24 h before PE-eVLP transduction. Indeed, epegRNA supplementation greatly improved PE-eVLP editing efficiency by greater than eightfold on average, while sgRNA supplementation offered minor improvement to BE-eVLP editing efficiency in an analogous experiment (Fig. 2a and Extended Data Fig. 2a). These results reveal that epegRNA loading, delivery or lifetime limits PE-eVLP editing to a much greater extent than sgRNA loading or delivery impacts base editing with BE-eVLPs. In contrast, PE protein supplementation via transfection of plasmids expressing PEs did not improve PE-eVLP editing efficiency (Fig. 2a), consistent with the insufficiency of epegRNAs but not PE proteins in PE-eVLPs.

We previously observed that the apparent affinity of Cas9 for sgRNAs, pegRNAs and epegRNAs decreases in that order^4^ (Supplementary Note 1). To increase Cas9–epegRNA affinity, we adopted the F + E guide RNA scaffold^51^, which improves Cas9–guide RNA binding through a stem extension in the scaffold structure that provides an extended handle for Cas9 binding. The F + E guide RNA scaffold moderately improved PE-eVLP editing efficiencies (Extended Data Fig. 2b) and was used in all subsequent epegRNAs.

To further address the inefficiency of epegRNA packaging in v2.3 PE-eVLPs, we used the MCP–MS2 system^52^ as an additional mechanism to promote epegRNA packaging. Binding of the MS2 coat protein (MCP) to the MS2 RNA stem–loop can mediate sgRNA packaging for Cas9 nuclease and BE delivery^53,54^, can recruit an untethered RT to Cas9 nickase in split PE systems^24,55^, and can recruit additional protein domains to PEs^56^. To apply this system to PE:epegRNA RNP packaging in eVLPs, we designed a Gag–MCP–Pol fusion plasmid by inserting one copy of MCP between the C-terminus of the Gag nucleocapsid domain and the N-terminus of the Pol domain (Fig. 2b). We reasoned that MCP insertion at this position would minimally disturb Gag polyprotein processing for eVLP particle maturation (Extended Data Fig. 2c and Supplementary Note 2) and that fused MCP would be presented at the interior of the particle, facilitating recruitment of epegRNAs into eVLPs.

Next, we tested a series of epegRNAs with MS2 stem–loop insertions in scaffold loops or at the 3′ end. We observed that inserting one copy of the MS2 stem–loop in the ST2 loop of epegRNAs (MS2-epegRNAs) led to the highest level of prime editing (Fig. 2c). Increasing the copy number of both MCP and MS2 aptamers impaired editing efficiency, probably because the larger fusions destabilize Gag–Pol and epegRNAs (Extended Data Fig. 2d,e). Lastly, we reoptimized the stoichiometry of Gag-fusion plasmids by varying the ratio of wild-type Gag–Pol plasmid, Gag–PE plasmid and Gag–MCP–Pol plasmid (Fig. 2d). The optimized ratio of 5:2:1 Gag–Pol plasmid:Gag–MCP–Pol plasmid:Gag–PE plasmid resulted in v3 PE-eVLPs that improved average prime editing efficiency by 4.0-fold over v2.3 PE-eVLPs at *Dnmt1* locus in N2A cells at the highest dose tested (Fig. 2e).

### PE3 further improves prime editing with PE-eVLPs

The PE-eVLP system described thus far packages one PE and one epegRNA (the ‘PE2 strategy’). The ‘PE3 strategy’ adds an additional ngRNA that directs the PE to nick the unedited strand, stimulating its replacement using the edited strand as a template, increasing editing efficiency at the cost of increased indel frequencies^1^. To enable PE3-eVLPs, we tested the possibility of packaging the ngRNA in the same or in a separate particle as the epegRNA. To screen for an optimal all-in-one particle v3 PE3-eVLP system, we transfected the producer cells with varying ratios of MS2-epegRNA and MS2-ngRNA plasmids simultaneously, along with other PE-eVLP components. The results demonstrated that transfecting MS2-epegRNA and MS2-ngRNA plasmids in an optimized ratio of 4:1 led to v3 PE3-eVLPs that support 1.3-fold higher average editing efficiency over the v3 PE2-eVLP system at *Dnmt1* locus in N2A cells with 25 μl of eVLPs (Fig. 2f). Alternatively, producing separate eVLP batches packaging PE:MS2-epegRNA RNP or PE:MS2-ngRNA RNP and transducing target cells with both eVLPs yielded comparable editing levels (Fig. 2f). Given the comparable levels of editing with two strategies, we moved forward with the all-in-one particle v3 PE3-eVLP system for the ease of production, ability to precisely control epegRNA and ngRNA stoichiometry, and potentially higher in vivo delivery efficiency from a single-particle system that does not require co-transduction by multiple eVLPs.

### 5′-G extension removal enhances editing with certain epegRNAs

Guide RNAs are transcribed from the U6 promoter in the PE-eVLP system, with a preference for initiating from guanine (G) at the +1 position^57^. epegRNAs that do not start with a 5′ G are often appended with an extra G at their 5′ end. Such a 5′-G extension may sterically hinder pegRNA binding to the Cas9 domain^58^, however, and thus may introduce an additional challenge for PE:epegRNA complex formation. Moreover, the unpaired 5′ G may impede R-loop formation and DNA cleavage by Cas9 nuclease in a target sequence-dependent manner^59^. Because PE:epegRNA RNPs delivered by PE-eVLPs are short lived by design, we reasoned that any impairment of the rate of target search and DNA nicking may be detrimental to PE-eVLP-mediated prime editing efficiencies.

**Extended Data Fig. 3:**
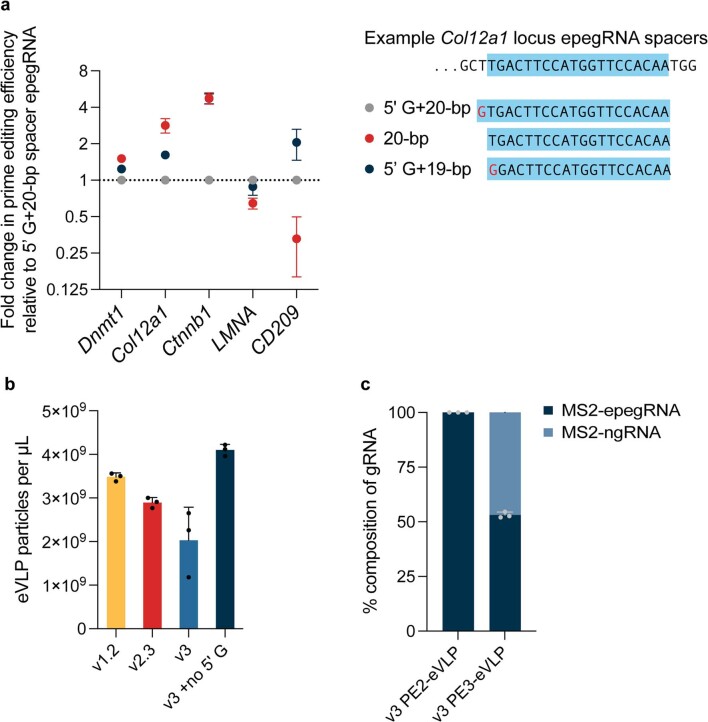
Optimization of eVLP cargo loading and delivery. **a**, Fold change in PE-eVLP editing efficiency compared to the original mismatched 5’ G + 20-bp epegRNA protospacer. **b**, Quantification of the number of eVLP particles per unit volume in preparations of successive generations of PE-eVLPs by anti-MLV p30 ELISA. These quantification data were used in experiments to determine the number of prime editor protein and epegRNA molecules per eVLP shown in Fig. 2g,h. **c**, Percentage of epegRNA and ngRNA composition in v3 PE2-eVLPs and v3 PE3-eVLPs. Data represent the average value of three technical replicates and error bars represent the standard deviation.

To overcome this potential hindrance, we tested v3 PE-eVLP systems that use epegRNAs containing either a 20-bp spacer starting with cytosine (C), uracil (U) or adenine (A), or a mismatched 5′ G followed by a 19-bp spacer (Extended Data Fig. 3a). The resulting data indicates that the improvement from omitting the 5′-G extension can be substantial, although the effect varies depending on the target sequence. At the *Col12a1* locus in N2A cells, a 20-bp spacer beginning with a 5′ T outperformed the original 5′ G + 20-bp spacer by 2.8-fold (Extended Data Fig. 3a). At the *Ctnnb1* locus, both the 20-bp spacer beginning with a 5′ A and the 5′ G + 19-bp spacer yielded comparable results, both offering 4.7-fold improvement over the original 5′ G + 20-bp spacer.

### Characterization of PE-eVLP cargo loading

To validate the mechanisms used to enhance cargo packaging, we quantified the epegRNAs and PE protein packaged in each PE-eVLP version. Intact epegRNAs were quantified by RT–quantitative polymerase chain reaction (qPCR) of total RNA extracted from eVLPs (Fig. 2g). The number of eVLP particles per unit volume measured using anti-MLV p30 (capsid) enzyme-linked immunosorbent assay (ELISA; Extended Data Fig. 3b) was used to calculate the number of epegRNAs packaged per eVLP. Although the PE and eVLP architecture engineering in v2.3 PE-eVLP was not designed to enhance epegRNA recruitment, v2.3 PE-eVLPs still improved epegRNA packaging over v1.2 PE-eVLPs by 2.1-fold, presumably because epegRNA packaging occurs as a complex with PE protein and therefore is correlated with PE protein packaging. The MCP–MS2 strategy in the v3 PE-eVLP system improved epegRNA loading by 2.2-fold compared to v2.3 PE-eVLPs, consistent with improved editing efficiency. Lastly, omitting the 5′-G extension on the epegRNAs offered an additional 1.3-fold improvement in epegRNA loading, supporting the hypothesis that the improvement in editing efficiency from extended 5′-G omission may arise from improved binding of epegRNAs to PEs. Additional characterization of the percent composition of MS2-epegRNA and MS2-ngRNA in v3 PE-eVLPs revealed an approximate 1:1 ratio of the two species (Extended Data Fig. 3c). Since the composition of MS2-ngRNAs increased in the eVLPs compared to the 4:1 stoichiometry used for eVLP production, we hypothesize that ngRNAs have higher binding affinity than epegRNAs for PE protein, resulting in more efficient loading into eVLPs.

ELISA with anti-Cas9 antibodies revealed an increase in PE protein per particle in successive versions of PE-eVLPs (Fig. 2h). The MS2–MCP strategy in v3 PE-eVLP improved both epegRNA recruitment and PE protein levels per particle by 1.3-fold, probably due to the three-component interaction between MCP:MS2-gRNAs:PE that synergistically enhances incorporation of all prime editing components (Fig. 2b). Likewise, omitting the 5′-G extension further improved the PE packaging by an additional 1.4-fold to an average of 26 molecules of PE protein per eVLP (Fig. 2h). Overall, these findings reveal that increases in both epegRNA and PE protein packaging efficiency in PE-eVLPs correlate with improvements in prime editing efficiencies over successive generations of PE-eVLPs, suggesting that v3 PE-eVLPs enhance prime editing outcomes by addressing bottlenecks in protein and RNA packaging.

### PE recruitment via coiled-coil peptide association

**Fig. 3:**
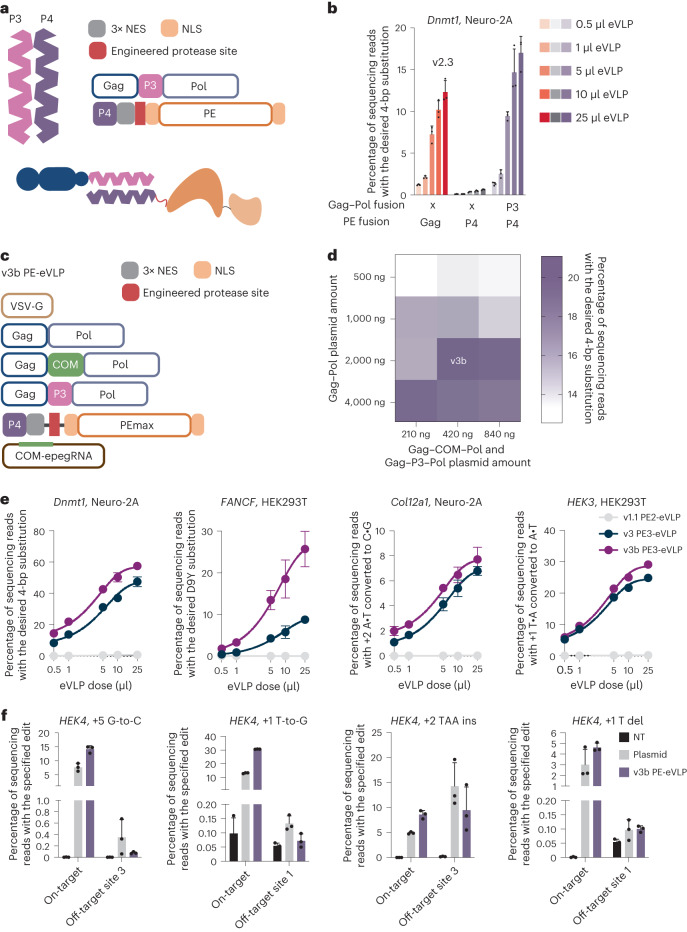
Development of v3b PE-eVLPs, which use an alternative PE recruitment mechanism. **a**, Schematic of coiled-coil peptide-dependent recruitment of PE. NES, nuclear export signal. NLS, nuclear localization signal. **b**, Comparison of prime editing efficiency with v2.3 PE-eVLPs versus the alternative system employing coiled-coil peptide for the recruitment of PE. **c**, Schematic of v3b PE-eVLP system. NES, nuclear export signal. NLS, nuclear localization signal. **d**, Heatmap of editing efficiencies from stoichiometry optimization of Gag–Pol, Gag–COM–Pol and Gag–P3–Pol plasmids for production of v3b PE-eVLPs. **e**, Prime editing efficiencies comparing v1.1 PE2-eVLPs, v3 PE3-eVLPs and v3b PE3-eVLPs at *Dnmt1*, *FANCF*, *Col12a1* and *HEK3* locus in N2A and HEK293T cells. **f**, Comparison of editing efficiencies for four different prime edits targeting *HEK4* locus in HEK293T cells following plasmid transfection or treatment with v3b PE-eVLPs at the on-target *HEK4* site and known off-target sites for the corresponding pegRNA^1^. Values shown in all graphs and heatmaps represent the average prime editing efficiency of three biological replicates, and error bars represent the standard deviation. Data were fitted to four-parameter logistic curves using nonlinear regression. For all conditions other than **f**, 30,000–35,000 cells were treated with eVLPs containing ~2.5 × 10^8^ eVLPs μl^−1^.

**Extended Data Fig. 4:**
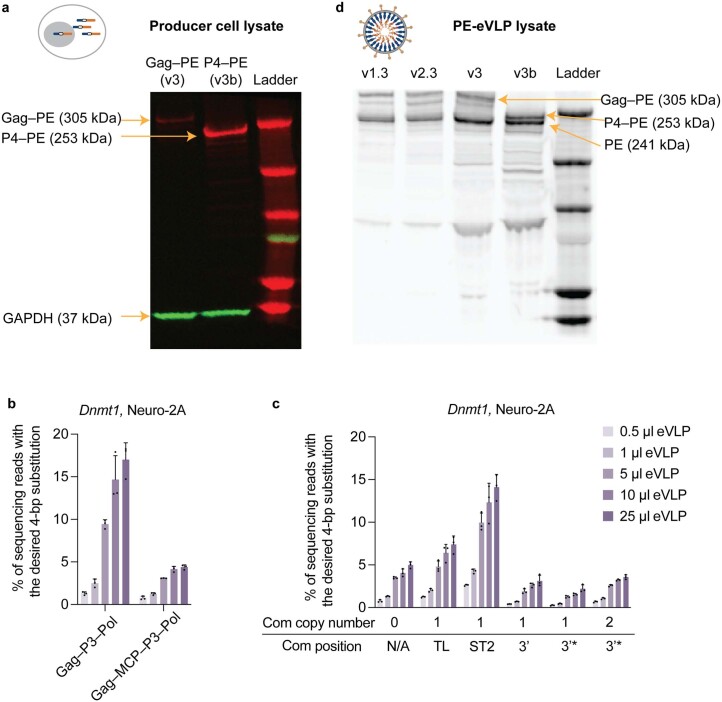
v3b PE-eVLP optimization and characterization. **a**, Representative western blot comparing expression of the gag-PE fusion protein from v3 PE-eVLPs versus the P4–PE fusion protein from v3b PE-eVLPs in producer cells transfected with the corresponding fusion proteins. **b**, Prime editing efficiencies of v3b PE-eVLPs with Gag–P3–Pol or Gag–MCP–P3–Pol. The Gag–MCP–P3–Pol fusion construct is not compatible with the efficient production of PE-eVLPs. **c**, Editing efficiencies of v3b PE-eVLPs at the *Dnmt1* locus in N2A cells with the Com aptamer inserted at various locations in the epegRNAs. The position of the Com aptamer insertion is as follows: 3’ denotes v3b PE-eVLPs with insertion of the Com aptamer after the structured tevoPreQ1 motif of the epegRNA; 3’* denotes v3b PE-eVLPs with insertion of the Com aptamer directly after the 3’-extension of the pegRNA, thereby using the Com aptamer to mimic a structured motif at the 3’ end of epegRNAs; TL denotes v3b PE-eVLPs with insertion of the Com aptamer within the tetraloop of the pegRNA scaffold; ST2 denotes v3b PE-eVLPs with insertion of the Com aptamer within the ST2 loop of the pegRNA scaffold. **d**, Representative western blot evaluating the amount of PE cargo packaged in v1.3, v2.3, v3 and v3b PE-eVLPs. Figures shown in (a) and (d) are representative images from two independently repeated experiments. Values shown in (b) and (c) represent the average prime editing efficiency of three biological replicates and error bars represent the standard deviation.

Although PE packaging in PE-eVLPs adopted the same strategy as BE packaging in v4 BE-eVLP and successive rounds of PE-specific optimizations led to an overall 2.0-fold average improvement in PE packaging in eVLPs, the absolute number of PE molecules packaged per particle (26 per eVLP) remained about half the value for BEs in BE-eVLPs (55 per eVLP)^44^. We hypothesized that the large size of the Gag–PE fusion protein (309 kDa) might hinder its production in the producer cells. To overcome this problem, we designed an alternative PE packaging system that does not require Gag fusion to PE (Fig. 3a). Instead, we used coiled-coil peptides, α-helical peptides that can strongly and specifically bind other α-helical peptides to form a superhelix structure^60^. We chose the P3–P4 coiled-coil peptide pair that has been previously used in mammalian cells to recruit protein domains to Cas9 (ref. ^61^). We designed Gag–P3–Pol fusion construct by fusing P3 to the C-terminus of the Gag nucleocapsid domain, and fused the partner P4 peptide, followed by 3× NES and the engineered protease cleavage site, to the N-terminus of PE (hereafter referred to as P4–PE). We produced PE-eVLPs by transfecting plasmids encoding VSV-G, wild-type MMLV Gag–Pol, Gag–P3–Pol, P4–PE and epegRNA into producer cells. This alternative system exhibited a 1.3-fold higher average prime editing efficiency compared to the analogous v2.3 Gag–PE-eVLP system at *Dnmt1* locus in N2A cells at the highest dose tested (Fig. 3b). The control eVLP lacking the P3 peptide showed 28-fold lower editing efficiency, supporting the role of P3–P4 association in facilitating PE packaging. Additionally, we confirmed via western blot of producer cell lysates substantially higher levels of P4–PE compared to Gag–PE (Extended Data Fig. 4a and Supplementary Note 3), consistent with our hypothesis that the smaller P4–PE fusion protein improved protein production levels.

Next, we explored the synergy of this alternative system with the pegRNA recruitment strategy using aptamer-binding proteins, which yielded substantial improvements in the v3 PE-eVLP system. Initial attempts using a single Gag–MCP–P3–Pol fusion construct proved inefficient (Extended Data Fig. 4b). Therefore, we tested PE-eVLP production using both Gag–aptamer-binding proteins–Pol and Gag–P3–Pol constructs. Instead of the MCP–MS2 pair, we used the COM–Com protein–RNA aptamer pair^38^ (Extended Data Fig. 4c), reasoning that the smaller size of both the COM protein and the Com aptamer may be less perturbative to particle maturation or PE function. We optimized the stoichiometry of Gag–COM–Pol, Gag–P3–Pol and P4–PE plasmids to yield the v3b PE-eVLP system, an alternative to v3 PE-eVLP system (Fig. 3c and Fig. 3d). We compared the v3 PE3-eVLP and v3b PE3-eVLP system at multiple loci and observed locus-dependent differences in editing efficiency (Fig. 3e). At the *Dnmt1* and *FANCF* loci, v3b outperformed v3 in prime editing efficiency by 1.2- and 2.9-fold, respectively, at the highest dose tested. At *HEK3* and *Col12a*1 loci, the two systems performed comparably, consistent with comparable levels of PE protein packaged in v3 and v3b PE-eVLPs analyzed via western blot (Extended Data Fig. 4d and Supplementary Note 3).

**Extended Data Fig. 5:**
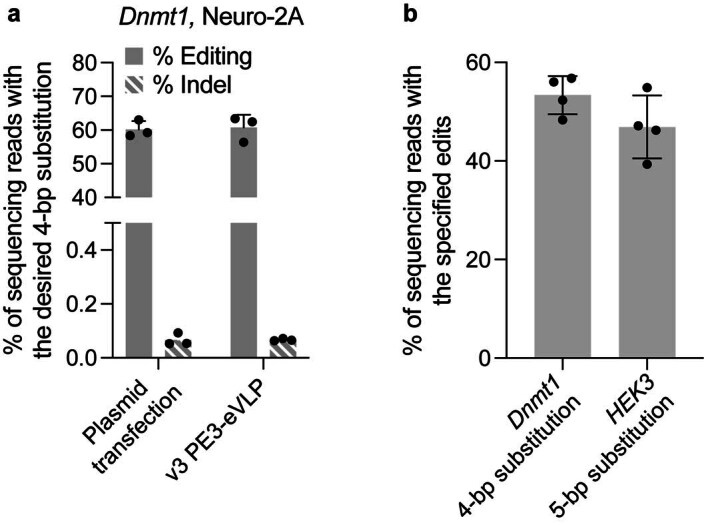
Characterization of PE-eVLPs. **a**, Comparison of prime editing efficiency (% editing) and insertion-deletion byproduct generation (% indel) of PE3 system delivered by plasmid transfection versus v3 PE3-eVLP. PE3 system targets *Dnmt1* locus in N2A cells. Data represent the average prime editing efficiency of three biological replicates and error bars represent the standard deviation. **b**, Prime editing efficiencies at *Dnmt1* locus in N2A cells and *HEK3* locus in HEK293T cells from treatment with four independent batches of v3 PE3-eVLPs produced on different days, with each dot indicating the prime editing efficiency of each of the four v3 PE3-eVLP batches. Data shown represent the mean prime editing efficiency from four different v3 PE3-eVLP batches and error bars represent the standard deviation.

**Extended Data Fig. 6:**
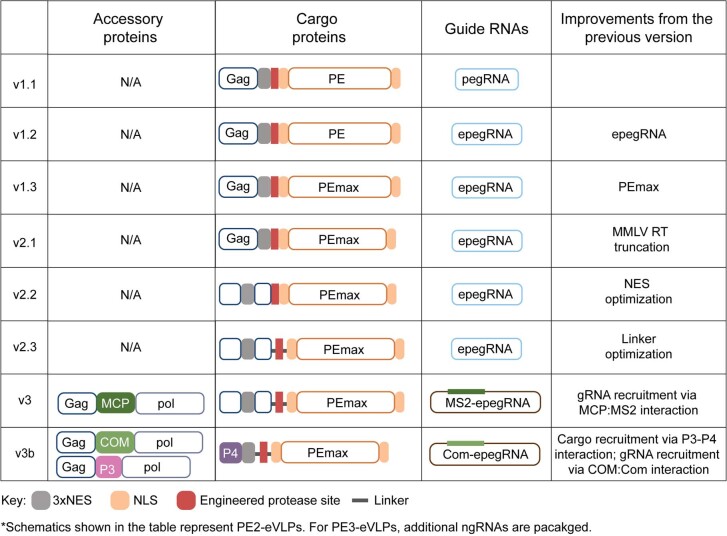
Schematic summary of PE-eVLP designs. Schematic of accessory proteins, cargo proteins, guide RNA designs, and description of improvements from the previous version for successive generations of PE-eVLPs. Envelope protein VSV-G, and capsid protein MMLV Gag–Pol that are common in all versions of PE-eVLPs are omitted from the table. Schematics shown in the table represent PE2-eVLPs. For PE3-eVLPs, additional ngRNAs are packaged at a ratio of 4:1 for pegRNAs and ngRNAs with corresponding scaffold modification and aptamer insertion.

The cumulative improvements in v3 and v3b PE3-eVLPs yielded large increases in prime editing efficiencies compared to the v1.1 PE2-eVLPs (Fig. 3e). v3 and v3b PE3-eVLPs outperformed v1.1 PE2-eVLPs by 79- and 65-fold at the *Dnmt1* locus in N2A cells, and 170- and 150-fold at the *HEK3* locus in HEK293T cells, respectively. At the *Col12a1* locus in N2A cells and *FANCF* locus in HEK293T cells, we did not observe any editing above background level (<0.1%) with v1.1 PE2-eVLPs, while the third-generation PE-eVLP architectures achieved on average 7.7% and 26% editing at the respective loci at the highest dose tested. Examination of the frequency of insertion–deletion byproducts revealed no apparent difference between PEs delivered by PE-eVLPs versus by plasmid transfection (Extended Data Fig. 5a). Furthermore, we observed low batch-to-batch variability as evidenced by consistent prime editing efficiencies using four independent batches of PE-eVLPs prepared on different days (Extended Data Fig. 5b). These data collectively highlight substantial benefits that resulted from the systematic engineering of third-generation PE-eVLP architectures (Extended Data Fig. 6).

### BE-eVLPs benefit from the engineered PE-eVLPs architecture

**Extended Data Fig. 7:**
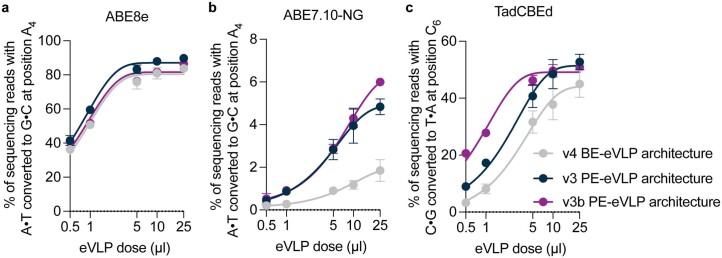
BE-eVLPs benefit from the engineered architectures of v3 and v3b PE-eVLPs. **a-c**, Comparison of base editing efficiencies of (a) ABE8e, (b) ABE7.10-NG, and (c) TadCBE at the *BCL11A* locus in HEK293T cells treated with eVLPs that use the v4 BE-eVLP architecture, the v3 PE-eVLP architecture, or the v3b PE-eVLP architecture. Values shown in all graphs represent the average base editing efficiency of three biological replicates and error bars represent the standard deviation. Data were fitted to four-parameter logistic curves using nonlinear regression.

Given the substantial improvements in prime editing efficiencies offered by v3 and v3b PE-eVLPs, we sought to further evaluate whether these alterations also enhance eVLP-mediated BE delivery. We packaged two ABEs, ABE8e^62^ and ABE7.10-NG^63^, as well as cytosine BE, TadCBEd^64^, in the previously reported v4 BE-eVLP architecture, and in the v3 or v3b PE-eVLP architectures (replacing the PE with either BE), targeting the *BCL11A* locus in HEK293T cells (Extended Data Fig. 7a–c). While the v3 and v3b PE-eVLP architecture offered minor improvements for highly active ABE8e, they substantially enhanced editing efficiencies for less active BEs such as ABE7.10-NG. For ABE7.10-NG, v3 and v3b PE-eVLP architectures yielded 2.6-fold and 3.2-fold higher base editing efficiencies, respectively, at the highest dose tested (Extended Data Fig. 7b). These results suggest that some of the strategies that improved PE protein and pegRNA packaging can also benefit the packaging and post-transduction editing efficiency of other gene editing cargoes.

### Transient delivery of PE by eVLPs reduces off-target editing

Previous studies have shown that transient delivery of genome editing agents can reduce off-target editing^44,46,65,66^. Direct delivery of RNP complexes in theory should result in the shortest duration of editing agent exposure to the genome compared to mRNA or DNA delivery. Prime editing has been shown to exhibit high DNA specificity, probably due to the requirement of three distinct DNA hybridization steps for productive editing^1,12–21,66^. Given the difficulty of finding genomic loci that support readily measured levels of off-target prime editing, we measured off-target editing from PE-eVLPs compared to plasmid transfection at the two most highly edited off-target sites discovered in a previous off-target prime editing profiling experiment^1^. Despite comparable or higher levels of on-target editing, off-target editing at these two unusually prone off-target sites by PE-eVLPs was reduced compared to plasmid transfection in HEK293T cells 7 days after treatment (Fig. 3f). For example, for a +2 TAA insertion edit, the ratio of on-target to off-target editing improved by 3.0-fold for PE-eVLPs compared to plasmid transfection (Fig. 3f). These findings confirm that the frequency of off-target prime editing can be further reduced by transient delivery of PE RNPs using PE-eVLPs.

### PE-eVLPs mediate potent in vivo prime editing in the brain

Next, we tested the ability of v3 and v3b PE-eVLPs to mediate in vivo prime editing in the mouse central nervous system (CNS). We produced v3 and v3b PE3-eVLPs programmed to perform a 4-bp substitution at the *Dnmt1* locus. We injected PE3-eVLPs into C57BL/6 mice via intracerebroventricular (ICV) injection on postnatal day 0 (P0), a stage at which the brain undergoes rapid development^67,68^. We co-injected VSV-G-pseudotyped lentivirus encoding EGFP fused to a nuclear membrane-localized Klarsicht/ANC-1/Syne-1 homology (KASH) domain to enrich cells that had the opportunity to encounter eVLPs.

**Fig. 4:**
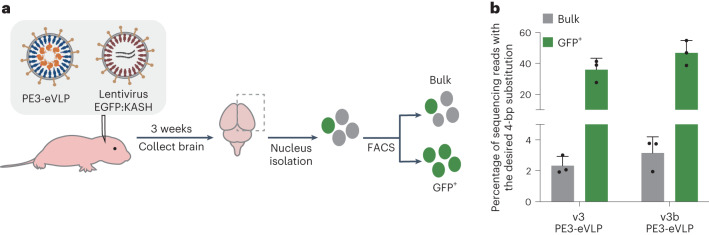
CNS editing with PE-eVLPs via P0 ICV injection. **a**, Schematic of workflow for neonatal ICV injection and subsequent analysis. FACS, fluorescence-activated cell sorting. **b**, Prime editing efficiency in bulk or GFP-positive population from the brain cortex collected 3 weeks following P0 ICV injection targeting the *Dnmt1* locus with v3 PE3-eVLPs and v3b PE3-eVLPs. Bars represent the average prime editing efficiency of three mice and error bars represent the standard deviation, with each dot representing an individual mouse. Each mouse received approximately 1.0 × 10^11^ eVLPs in total.

**Extended Data Fig. 8:**
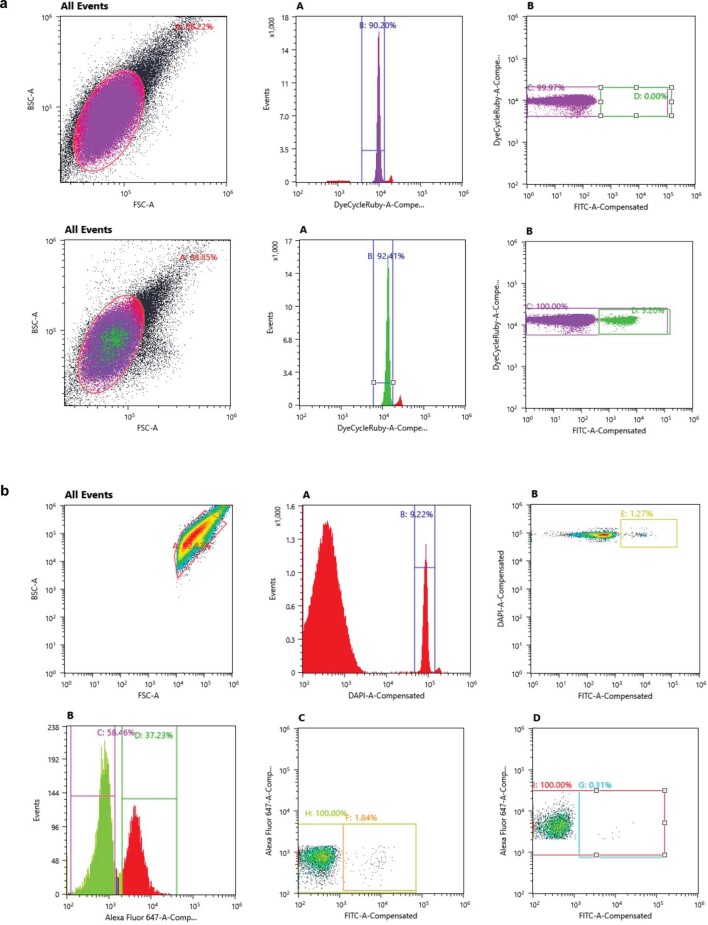
Example FACS gating for single nucleus sorting. **a**, Single nucleus was gated based on forward scatter (FSC-A) and back scatter (BCS-A) ratios and DyeCycle Ruby signal. GFP-positive nuclei were gated based on the FITC signal. The first row displays representative FACS data for untreated samples and the second row displays representative FACS data for cortex samples harvested from neonatal mice co-injected with 4 μl PE-eVLPs and 0.3 μl VSV-G pseudotyped GFP:KASH lentivirus via ICV injection. Bulk nuclei correspond to events that passed gate C and GFP-positive nuclei correspond to events that passed gate D. **b**, Example FACS gating for neuron-specific sorting. Single nucleus was gated based on forward scatter (FSC-A) and back scatter (BCS-A) ratios and DAPI signal. The signal from Alexa 647-conjugated NeuN antibody distinguishes NeuN-positive and NeuN-negative populations. GFP-positive nuclei were gated based on FITC signal in both NeuN-positive and NeuN-negative populations. Gates displayed represent FACS data for midbrain samples harvested from neonatal mice co-injected with 4 μl PE-eVLPs and 0.3 μl VSV-G pseudotyped GFP:KASH lentivirus via ICV injection. Gate F represents GFP-positive nuclei from the NeuN-negative population. Gate G represents GFP-positive nuclei from the NeuN-positive population.

We collected the brain hemispheres 3 weeks after injection, isolated both bulk and green fluorescent protein (GFP)^+^ nuclei (Extended Data Fig. 8a), extracted genomic DNA and performed high-throughput sequencing (HTS) (Fig. 4a). We achieved on average 2.3% bulk cortex editing and 36% editing among GFP+ nuclei with v3 PE3-eVLPs (Fig. 4b). The v3b PE3-eVLPs that showed more efficient editing in cell culture outperformed the v3 PE3-eVLP construct in vivo, achieving 3.2% editing in bulk cortex and 47% average editing among GFP+ nuclei (Fig. 4b). Efficient prime editing among GFP+ nuclei in the brain indicates that PE RNPs delivered by PE-eVLPs can induce efficient prime editing in cells that are transduced. As we previously reported^44^, however, transduction events occur primarily near the site of injection, probably due to the slow rate of diffusion of eVLPs (~117 nm in diameter as measured by dynamic light scattering (DLS)) within the brain. Staining isolated nuclei with neuron-specific anti-NeuN antibody^69^ revealed that most GFP^+^ nuclei were NeuN negative, suggesting VSV-G pseudotyped GFP:KASH lentivirus and PE-eVLPs primarily transduced nonneuronal cells (Extended Data Fig. 8b). Given the ability of eVLPs to alter tropism by using alternative envelope proteins^44^, using a neuron-specific envelope protein such as FuG-B2 (ref. ^70^) may enable more efficient targeting of neurons.

### PE-eVLP corrects mutations causing retinal diseases in vivo

**Fig. 5:**
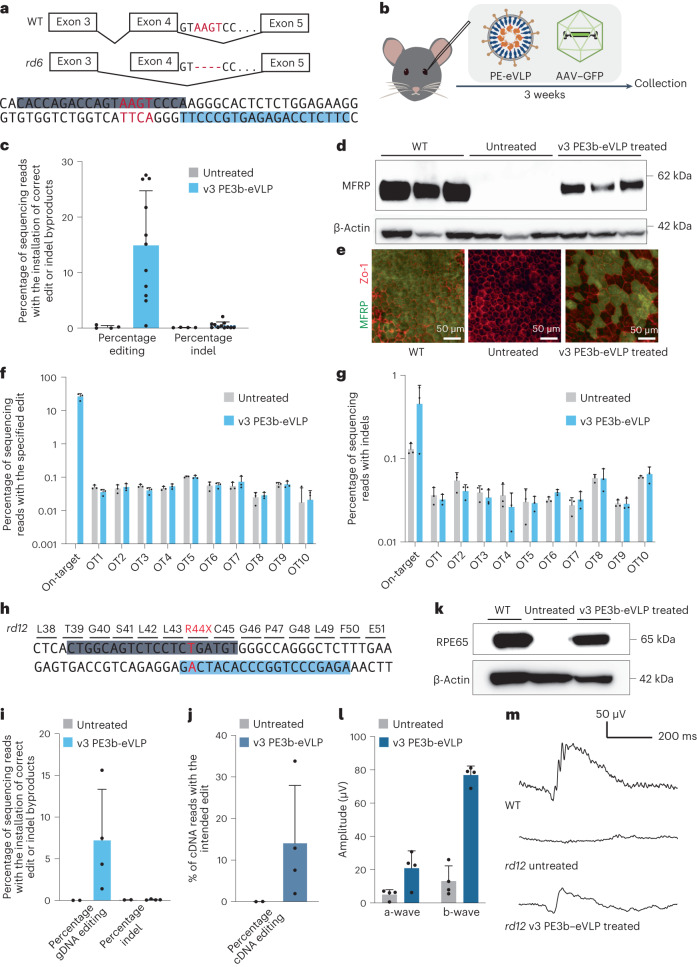
Retinal disease correction in vivo with PE-eVLPs. **a**, An optimized PE3b strategy to correct a 4-bp deletion in *Mfrp* in the *rd6* mice. The mutation (red), the epegRNA spacer (light blue) and the ngRNA spacer sequence (dark blue) are highlighted. **b**, Schematic of subretinal injection of *rd6* mice. **c**, Prime editing efficiency and the associated indel frequency in the genomic DNA collected from the *rd6* mice. Data are represented as mean values ± s.e.m. Each dot represents an individual mouse for *n* = 4 (untreated) or *n* = 11 (v3 PE3b-eVLP treated). **d**,**e**, Western blot of RPE tissue protein extracts (**d**) and immunohistochemistry blot on RPE flatmounts (**e**) from wild-type C57BL/6J mice, untreated *rd6* mice and v3 PE3-eVLP-treated *rd6* mice. Flatmounts were stained with anti-MFRP antibody (green) and anti-ZO-1 antibody (red). **f**,**g**, Analysis of PE-dependent editing (**f**) and indel byproducts (**g**) at the on-target site and top ten CIRCLE-seq nominated off-target sites associated with the *rd6* epegRNA (**f**) and ngRNA (**g**) sequence. For **f** and **g**, data are represented as mean values ± s.e.m. Each dot represents an individual mouse for *n* = 3 (untreated) or *n* = 3 (v3 PE3b-eVLP treated). **h**, The R44X mutation in *Rpe65* exon 3 in the *rd12* mouse model and the optimized PE3b strategy used for correction of this mutation. The mutation (red), the epegRNA spacer (light blue) and the ngRNA spacer sequence (dark blue) are highlighted. **i**, Prime editing efficiency of *Rpe65* R44X mutation correction and the associated indel frequency in genomic DNA collected from *rd12* mice. **j**, Prime editing efficiency of *Rpe65* R44X mutation correction in *Rpe65* RNA collected from *rd12* mice. **k**, Western blot of RPE tissue protein extracts from wild-type C57BL/6J mice, untreated *rd12* mice and v3 PE3b-eVLP-treated *rd12* mice. **l**, Scotopic A- and B-wave amplitudes measured by ERG following overnight dark adaptation. For **i**, **j** and **l**, data are represented as mean values ± s.e.m. Each dot represents an individual mouse for *n* = 4 (untreated) or *n* = 4 (v3 PE3b-eVLP treated). **m**, Representative ERG waveforms from wild-type C57BL/6J mice, untreated *rd12* mice and v3 PE3-eVLP-treated *rd12* mice. Each mouse received approximately 4.2 × 10^10^ eVLPs per eye. WT, wild type. Source data

Next, we sought to test the therapeutic utility of in vivo PE delivery via PE-eVLPs by correcting a mutation causing retinal disease in *rd6* mice, a model of autosomal recessive retinal degeneration caused by a 4-bp deletion in the splice donor of the membrane-type frizzled-related protein (*Mfrp*) gene^71^ (Fig. 5a). The mutation leads to early onset, slowly progressive retinal degeneration in mice, akin to genetic defects in *MFRP* associated with microphthalmos and retinitis pigmentosa in humans^72^. Given the unique ability of prime editing to directly and precisely correct a 4-bp deletion, and the potential of prime editing to treat congenital blindness^73^, we developed a PE3b strategy—in which the ngRNA is designed to nick only after the desired prime edit is installed into the edited strand^1,7^—to correct this 4-bp deletion in *Mfrp* (Fig. 5a). Following plasmid transfection in N2A^*Mfrp*^ cells that contained the 4-bp deletion at the endogenous locus, the best PE3b strategy yielded on average 18% correction of the mutation with 0.66% indels (Supplementary Table 4). Encouraged by this result, we produced v3 PE3b-eVLPs pseudotyped with VSV-G to efficiently transduce the retinal pigment epithelium (RPE) cells^44,74,75^, where MFRP is most abundantly expressed^71^.

**Extended Data Fig. 9:**
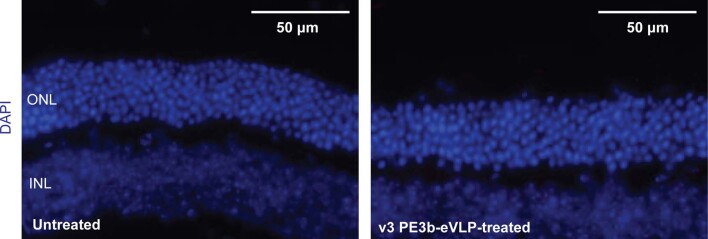
Immunohistochemistry blot on eye cryosections of rd6 mice. Retina cryosections from untreated *rd6* mice and v3 PE3b-eVLP-treated *rd6* mice were stained with DAPI (blue). Figure shown is a representative image from two independently repeated experiments.

We performed subretinal injections into 5-week-old *rd6* mice to administer the mutation-correcting v3 PE3b-eVLPs (Fig. 5b). A total of 3 weeks post-injection, we performed HTS on the genomic DNA extracted from the RPE tissue. Sequencing analysis revealed on average 15% bulk retinal editing with v3 PE3b-eVLPs (Fig. 5c). Western blot revealed that MFRP expression was restored in v3 PE3b-eVLP-treated eyes (Fig. 5d). We performed immunohistochemistry on the RPE flatmounts and confirmed robust restoration of MFRP expression in the RPE (Fig. 5e). We assessed off-target prime editing from v3 PE3b-eVLP treatment by deep sequencing the top ten off-target sites associated with the *Mfrp* epegRNA and ngRNA as nominated by circularization for in vitro reporting of cleavage effects by sequencing (CIRCLE-seq)^76^. We did not detect any off-target editing in PE-eVLP-treated samples (>0.1%, the limit of detection of the targeted HTS method used) (Fig. 5f,g). These data are consistent with the inherently high DNA specificity of prime editing^1,12–21,66^, further enhanced by transient exposure of cells to PEs from PE-eVLPs. Additionally, no signs of toxicity or morphology changes were observed in treated eye cryosections (Extended Data Fig. 9).

Finally, to further test the ability of PE-eVLPs to mediate therapeutic in vivo prime editing, we applied PE-eVLPs to treat the *rd12* mouse model^77^, which displays more severe retinal degeneration that allows evaluation of disease phenotype rescue. The *rd12* model contains a nonsense mutation in exon 3 of the retinal pigment epithelium 65 (*Rpe65*) gene (c.130 C > T; p.R44X) (Fig. 5h), leading to diminishment of electroretinogram (ERG) responses from 3 weeks of age^77^. The corresponding mutation in humans in homozygous form causes Leber congenital amaurosis^78^. We previously demonstrated partial rescue of this disease phenotype using BE-eVLPs^44^. However, base editing at this site generates bystander edits at nearby A•T base pairs that may inactivate the RPE65 enzyme and cause adverse effects^75^, whereas the mechanism of prime editing inherently avoids bystander editing^1^.

We developed a PE3b strategy to correct the *Rpe65* R44X mutation (Fig. 5h) by screening a panel of epegRNAs with varying primer binding site lengths (8–11 nt) and reverse transcription template lengths (11–25 nt), and seven candidate ngRNAs via plasmid transfection in an engineered NIH 3T3 cell line^75^ containing the corresponding mutation. The most promising PE3b strategy yielded 46% precise correction with 0.58% indels (Supplementary Table 5). We performed subretinal injection of 5-week-old *rd12* mice with mutation-correcting v3 PE3b-eVLPs. HTS on the genomic DNA extracted from RPE tissues of the treated mice resulted in on average 7.2% correction of the mutation in bulk RPE tissue (Fig. 5i). This editing efficiency is higher than the reported value from triple AAV^23^ and low-dose dual-vector AAV-mediated PE delivery^22^ and comparable to that achieved by high-dose dual-vector AAV-mediated PE delivery^22^, but using a virus-free, single-particle delivery method.

**Extended Data Fig. 10:**
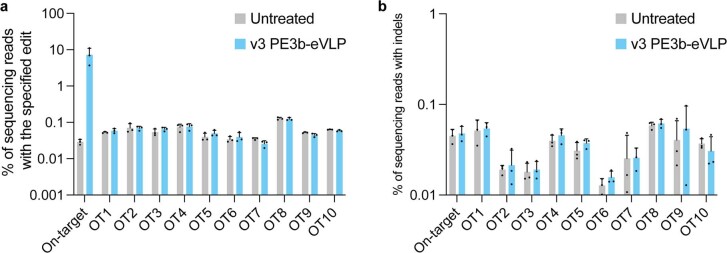
Off-target analysis in rd12 mice. **a**, Analysis of PE-dependent editing at the on-target site and at the top 10 CIRCLE-seq nominated off-target sites associated with the *rd12* epegRNA sequence. **b**, Analysis of indels at the on-target site and the top 10 CIRCLE-seq nominated off-target sites associated with the *rd12* ngRNA sequence. Bars represent average values for n = 3 (untreated) or n = 3 (v3 PE3b-eVLP-treated), with each dot representing an individual mouse and error bars representing standard deviation.

We further analyzed *Rpe65* transcripts in the extracted RNA by sequencing the complementary DNA. Editing was enriched at the transcript level, achieving on average 14% correction of the target mutation (Fig. 5j), presumably due to nonsense-mediated decay accelerating the degradation of uncorrected transcripts and enrichment of the major RPE65*-*expressing RPE cells that are preferentially transduced by PE-eVLPs among other cell types co-collected from tissue dissection. We confirmed robust expression of full-length RPE65 in v3 PE3b-eVLP-treated eyes by western blot (Fig. 5k). ERG of the v3 PE3b-eVLP-treated animals indeed showed substantial rescue of visual function in response to the stimuli compared to the untreated eyes (Fig. 5l,m). No off-target prime editing was detected above the 0.1% limit of detection at top ten CIRCLE-seq-nominated off-target sites associated with the *rd12* epegRNA and ngRNA (Extended Data Fig. 10a,b). Together, these results demonstrate that in vivo application of optimized PE-eVLPs can correct a pathogenic mutation and partially rescue disease phenotype in mammals.

## Discussion

Through extensive engineering of each major component, we developed an all-in-one virus-like particle that delivers PE RNPs into mammalian cells in culture and in vivo. Recent improvements to prime editing systems, including epegRNAs^4^, the PEmax architecture^2^ and MMR evasion^2^, contributed to improved outcomes with PE-eVLPs. Identification of bottlenecks in cargo packaging yielded PE variants that promote delivery by PE-eVLPs, as well as optimized eVLP architectures that facilitate cargo release and cargo localization. Introducing an additional mechanism for guide RNA recruitment addressed guide RNA packaging limitations, and an alternative v3b PE-eVLP system eliminated the need for covalent fusion to the Gag polyprotein. Together, these improvements yielded 170-fold higher average prime editing efficiency compared to v1.1 PE-eVLPs at a benchmark *HEK3* test edit in HEK293T cells.

The optimized v3 and v3b PE-eVLPs systems proved efficacious in vivo. Potent prime editing was achieved in the mouse CNS via neonatal ICV injection, marking the first demonstration of CNS editing with transient delivery of a PE RNP. In the mouse retina, a single injection of v3 PE3-eVLPs precisely corrected a pathogenic 4-bp deletion in the *rd6* model of retinal degeneration, restoring production of full-length MFRP protein. In the *rd12* mouse model of genetic blindness, v3 PE3-eVLPs achieved comparable prime editing levels to a recently reported triple-vector AAV–PE system^23^, but using a nonviral, single-particle delivery vehicle, resulting in partial rescue of visual function. These findings demonstrate that v3 and v3b PE-eVLPs can achieve prime editing efficiency comparable to that attained using an AAV–PE delivery system, while avoiding drawbacks of viral delivery systems such as prolonged editor expression that increases off-target editing frequencies and the risk of oncogenic DNA integration^29,79^. To our knowledge, these findings also represent the first use of PE RNPs to achieve phenotypic rescue of an animal model of genetic disease.

While v3 and v3b PE-eVLPs demonstrated therapeutically relevant editing levels, PE-eVLP systems would benefit from the continued engineering effort for the next-generation PEs and improved eVLP systems. Furthermore, tissue-specific envelope protein engineering could expand the scope of PE-eVLP applications to diverse tissues. The possibility that single-dose, transient delivery of PE RNPs by PE-eVLPs may mitigate clinically relevant immunogenicity^80^ warrants further investigation. Lastly, future optimization in large-scale eVLP production will be necessary to fully realize the therapeutic potential of eVLPs. Nonetheless, the PE-eVLP system reported here offers unique advantages of nonviral, single-particle delivery of PEs in their most transient form as RNPs, presenting safety and target specificity advantages over DNA or mRNA delivery methods.

## Methods

### Molecular cloning

All plasmids were cloned using either USER, Gibson or Golden Gate assembly. DNA was PCR amplified with PhusionU Green Multiplex PCR Master Mix (Thermo Fisher Scientific, F564S). Plasmids were transformed into Mach1 (Thermo Fisher Scientific, C862003) chemically competent *Escherichia* *coli* and were prepared using Plasmid Plus Midiprep kits (Qiagen, 12945).

### Cell culture

HEK293T cells (ATCC, CRL-3216), Neuro-2a cells (ATCC, CCL-131) and Gesicle Producer 293T cells (Takara, 632617) were cultured in Dulbecco’s modified Eagle medium (DMEM) plus GlutaMax (Life Technologies; 10569044) supplemented with 10% (v/v) fetal bovine serum (FBS). Cells were maintained at 37 °C with 5% CO_2_. Cell lines were confirmed to be negative for mycoplasma during this study.

### PE-eVLP production

eVLPs were produced as previously described^44^. Briefly, Gesicle Producer 293T cells were plated at a density of 5 × 10^6^ cells per flask in 10 ml of DMEM + 10% FBS media in T75 flask (Corning, 353136). A total of 18–24 h after seeding, a mixture of plasmids was transfected to producer cells with jetPRIME transfection reagent (Polyplus, 101000001) following the manufacturer’s protocol. For production of v3 PE-eVLPs, plasmids expressing VSV-G (400 ng), wild-type MMLV Gag–Pol (2,813 ng), Gag–MCP–Pol (1,125 ng), Gag–PE (563 ng) and MS2-guide RNA (4,400 ng MS2-epegRNA for v3 PE2-eVLP, 3520 ng MS2-epegRNA and 880 ng MS2-ngRNA for v3 PE3-eVLP) were co-transfected to each T75 flask. For production of v3b PE-eVLPs, plasmids expressing VSV-G (400 ng), wild-type MMLV Gag–Pol (2,813 ng), Gag–COM–Pol (2,000 ng), Gag–P3–Pol (422 ng), P4–PE (422 ng) and COM-gRNAs (4,400 ng COM-epegRNA for v3b PE2-eVLP, 3,520 ng COM-epegRNA and 880 ng COM-ngRNA for v3b PE3-eVLP) were co-transfected to each T75 flask. A total of 40–48 h after transfection, supernatants were collected, centrifuged at 500*g* for 5 min, then the supernatant was filtered through 0.45-μm polyvinylidene difluoride (PVDF) filter. For PE-eVLPs used with cultured cells, 5× PEG-it Virus Precipitation Solution (System Biosciences, LV825A-1) was subsequently added to the supernatant to precipitate eVLPs overnight at 4 °C. The next day, the eVLPs were pelleted by centrifugation at 1,500*g* for 30 min at 4 °C and were concentrated 100-fold by resuspending in 100 μl of Opti-MEM (Life Technologies; 31985070). All eVLPs tested for optimization experiments in cell culture were concentrated uniformly using the above mentioned method to facilitate direct comparison of PE-eVLP potency at the same volume of eVLPs transduced. PE-eVLPs concentrated by this method contain approximately 2.5 × 10^8^ eVLPs μl^−1^. For PE-eVLPs used in vivo, eVLPs were concentrated using a 20% (w/v) sucrose in phosphate-buffered saline (PBS) cushion solution via ultracentrifugation at 26,000 rpm (141,000*g* for an rAV of 118.2 mm) for 2 h at 4 °C using an SW28 rotor in an Optima XPN Ultracentrifuge (Beckman Coulter). The eVLP pellets were resuspended in cold PBS solution following ultracentrifugation. The eVLP solution was further centrifuged at 1,000*g* for 5 min on a fixed-angle tabletop centrifuge to remove debris. eVLPs purified by ultracentrifugation and used for in vivo applications were resuspended in a minimum volume of PBS solution to maximize the dose of PE-eVLPs within the permitted volume of injection. For short-term storage, eVLPs were stored at 4 °C for up to 1 week. For long-term storage, eVLPs were stored at −80 °C and thawed on ice immediately before use. Repeated freeze–thaw was avoided.

### PE-eVLP transduction in cultured cells and genomic DNA collection

Target cells were plated at a density of 30,000–35,000 cells per well in 48-well plates (Corning, 354509). A total of 18–24 h after seeding, PE-eVLPs were added to the media of target cells. Unless otherwise noted, cellular genomic DNA was collected 72 h after transduction as previously described^44^. Briefly, medium was removed from each well and cells were washed with 1× PBS. Then 130 μl of lysis buffer (10 mM Tris–HCl pH 8.0, 0.05% SDS and 25 μg ml^−1^ proteinase K) was added to each well. Following incubation at 37 °C for 1 h, the lysate was heated to 80 °C for 30 min and was used directly as an input for downstream HTS preparation.

### HTS of genomic DNA samples

HTS was performed as described previously^1^. Primers used for the amplification of genomic loci and corresponding amplicons are listed in Supplementary Table 2. Briefly, 1–5 μl of cell lysate containing genomic DNA described above was used directly for the amplification of the target locus in the first round of PCR (PCR1). For base substitution edits, the target locus was amplified using Phusion U Green Multiplex PCR Master Mix (Thermo Fisher Scientific, F564S) under the following conditions: 98 °C (3 min); 30 cycles of 98 °C (10 s), 61 °C (20 s) and 72 °C (40 s); and 72 °C (2 min). For insertion and deletion edits that are more susceptible to PCR bias, PCR1 was monitored using SYBR Green fluorescence with qPCR and the reaction was stopped at the exponential phase to avoid over-amplification of the target locus. Subsequently, 1–2 μl of PCR1 product was used as a template for the second round of PCR (PCR2) to append unique Illumina barcodes. PCR2 was conducted using Phusion U Green Multiplex PCR Master Mix under the following condition: 98 °C (3 min); 10 cycles of 98 °C (10 s), 61 °C (20 s) and 72 °C (30 s); and 72 °C (2 min). PCR2 products were pooled and purified on 1.5 % agarose gel by gel extraction using QIAquick Gel Extraction Kit (Qiagen; 28704). The library was quantified by Qubit dsDNA HS Assay Kit (Thermo Scientific, Q32852) and was sequenced using Illumina MiSeq 300 v2 Kit (Illumina) on Illumina MiSeq instrument.

### HTS data analysis

HTS reads were demultiplexed using the MiSeq Reporter software v2.6 (Illumina). Data analysis was conducted using CRISPResso2 as previously described^2^. Briefly, reads were filtered by minimum average quality score (*Q* > 30) before analysis. CRISPResso2 analysis was performed with ‘discard_indel_reads’ on, and the quantification window was set to encompass at least ten nucleotides upstream and downstream of the pegRNA and/or ngRNA nick site. Prime editing efficiency was calculated as the percentage of reads with the desired editing without indels divided by the total number of reference-aligned reads. Indel frequency was calculated as the number of discarded reads divided by the total number of reference-aligned reads. The lower limit of detection is assumed to be 0.1%, defined by the error rate of the HTS method used.

### Plasmid transfection

Plasmid transfection for purposes other than eVLP production was performed using Lipofectamine 2000 (Invitrogen, 11668500) following the manufacturer’s protocol as described previously^1,2^. Briefly, cells were seeded in either 96-well plates (Corning, 353075) at a density of 15,000–20,000 cells per well or 48-well plates (Corning, 354509) at a density of 30,000–35,000 cells per well. A total of 16–24 h after seeding, test plasmids were mixed in Opti-MEM (Life Technologies, 31985070). For 96-well transfection, editor plasmids (250 ng) and guide RNA plasmids (40 ng epegRNA for PE2; 30 ng pegRNA and 10 ng ngRNA for PE3) were mixed with 0.5 μl of Lipofectamine 2000. For 48-well transfection, editor plasmids (750 ng) and guide RNA plasmids (250 ng epegRNA for PE2; 188 ng epegRNA and 62.5 ng ngRNA for PE3) were mixed with 1 μl of Lipofectamine 2000. Following incubation at room temperature for 10 min, the transfection mixture was added directly to the media of the target cells. Genomic DNA was collected 72 h after transfection following the protocol described above.

### PE-eVLP protein content quantification by ELISA

The protein content of PE-eVLPs was quantified as described previously^44^. Briefly, PE-eVLPs used for protein content quantification were concentrated via ultracentrifugation as described above for optimal detection of protein. A total of 5 μl of ultracentrifuged PE-eVLPs was mixed with 2× dye-free Laemmli sample buffer (100 mM Tris pH 7.5, 4% SDS and 20% (v/v) glycerol) and was incubated at 95 °C for 15 min. The lysed PE-eVLPs were used as input for quantification of PE protein and MLV p30 protein by ELISA. PE content in PE-eVLPs was quantified using the FastScan Cas9 (*Streptococcus* *pyogenes*) ELISA kit (Cell Signaling Technology, 29666C) following the manufacturer’s protocol. A standard curve was generated using recombinant Cas9 (*S. pyogenes*) nuclease protein (New England Biolabs, M0386). The number of eVLPs per volume was measured by quantifying MLV p30 content with the MuLV Core Antigen ELISA kit (Cell Biolabs, VPK-156) following the manufacturer’s protocol and calculated by assuming that 20% of the measured p30 in solution is associated with VLPs and that each VLP molecule contains 1,800 molecules of p30 (ref. ^81^).

### PE-eVLP pegRNA content quantification by RT–qPCR

PE-eVLPs used for pegRNA content quantification were concentrated via ultracentrifugation as described above. A total of 10 μl of ultracentrifuged PE-eVLPs was treated with DNase I (Qiagen, 79254) to remove any residual plasmid DNA carry-over. RNA was extracted from PE-eVLPs using QIAamp Viral RNA Mini Kit (Qiagen, 52906) following the manufacturer’s protocol.

For standard curve generation, guide RNAs (epegRNAs or ngRNAs) were transcribed in vitro using the HiScribe T7 High Yield RNA Synthesis Kit (New England Biolabs, E2040S) following the manufacturer’s protocol. RNA was purified using Monarch RNA Cleanup Kit (New England Biolabs; T2030S). In vitro-transcribed epegRNAs were subjected to the same DNase treatment and RNA extraction procedure as PE-eVLPs as described above.

Standard and test gRNAs extracted from PE-eVLPs were serially diluted and reverse-transcribed to generate cDNA using SuperScript IV Reverse Transcriptase (Invitrogen, 18090010) following manufacturer’s protocol. Briefly, a sequence-specific reverse primer that binds the 3′ end of gRNAs was annealed to the template RNA upon incubation at 65 °C for 5 min. RT mix was then added to the annealed RNA, and the reaction was incubated at 65 °C for 20 min, followed by 80 °C for 10 min. The cDNA generated was used as an input for qPCR. qPCR was performed using Power SYBR Green Master Mix (Applied Biosystems, 4368577) under the following condition: 95 °C (10 min), and 40 cycles of 95 °C (15 s) and 67 °C (1 min). Because all RNAs including gRNAs are potentially susceptible to degradation in cells, to exclusively quantify functional epegRNAs that retain their spacer, scaffold and 3′ extension, qPCR primers were designed to anneal to part of the spacer and scaffold at the 5′ end, and to part of the PBS and structured motif at the 3′ end. RT–qPCR primers are listed in Supplementary Table 3.

### DLS

DLS was performed with a Zetasizer Nano ZS (Malvern Panalytical). A total of 5 μl of PE-eVLPs purified by ultracentrifugation were diluted in 800 μl of PBS, and the samples were transferred to cuvettes for measurement. Backscatter (173°) measurements (*n* = 3 per sample) were taken each using ten runs of 8 s and an equilibration time of 10 s. The number size distribution was calculated using an estimated refractive index of 1.45 and absorption of 0.001 based on the preset values for proteins and phospholipids, and mean diameter reported represents the average size of three technical replicates.

### Western blot analysis of producer cell lysate protein content

Gesicle Producer 293T cells were plated at a density of 300,000–320,000 cells per well in six-well plates (Corning; 3506). A total of 16–24 h later, wild-type Gag–Pol plasmids (6,000 ng) and editor fusion plasmids (2,000 ng) were mixed with 8 μl of Lipofectamine 2000. A total of 48 h later, cells were washed with PBS and lysed in 200 µl of RIPA buffer (supplied by Broad Institute Internal Store; 2.5 mM sodium deoxycholic acid, 1 mM EDTA, 1% Triton X-100, 500 mM NaCl, 20 mM Tris–HCl pH 8 and 0.1% SDS in RODI water) supplemented with 1 mM phenylmethylsulfonyl fluoride (Sigma-Aldrich, 93482) and cOmplete Protease Inhibitor (Sigma-Aldrich, 4693159001) by incubating at 4 °C for 30 min. The lysate was centrifuged at 12,000 rpm (13,700*g*) for 20 min and the supernatant was collected. Total protein level was measured by bicinchoninic acid assay (Thermo Scientific, #23252) following the manufacturer’s protocol and samples were normalized on the basis of the protein concentration measured.

Western blots were performed as described previously^44^. Briefly, lysates were separated on a NuPAGE 3–8% Tris-acetate gel (Thermo Fisher Scientific; EA0376) in NuPAGE Tris-acetate SDS running buffer (Thermo Fisher Scientific, LA0041) for 45 min at 150 V. The gel was transferred to a PVDF membrane (Life Technologies, IB24002) using an iBlot2 Gel Transfer Device (Thermo Fisher Scientific, IB21001) at 20 V for 7 min. The membrane was blocked using Intercept Blocking Buffer (LI-COR, 927-70050) for 1 h at room temperature with gentle rocking. The membrane was washed three times with 1x TBS-Tween by rocking at room temperature for 5 min per wash. Then the membrane was incubated with primary antibodies (mouse Cas9 antibody: Thermo Fisher Scientific, #MA5-23519; rabbit GAPDH antibody: Cell Signaling Technology, #2118) at 1:1,000 dilution in Superblock Binding Buffer (1% bovine serum albumin (BSA) in TBS-Tween). The next day, the membrane was washed three times with 1× TBS-Tween as described above. Then the membrane was incubated with secondary antibodies (goat anti-mouse antibody: LI-COR IRDye 680RD and 926-68070, and goat anti-rabbit antibody: LI-COR IRDye 800RD and 926-32211) at 1:10,000 dilution in Superblock Binding Buffer for 1 h at room temperature with gentle rocking. The membrane was washed three times before imaging using a ChemiDoc MP Imaging System (Bio-Rad, 12003154).

### Western blot analysis of PE-eVLP protein content

PE-eVLPs were concentrated via ultracentrifugation and lysed in 2× dye-free Laemmli buffer as described before. Western blots were performed as described above, using mouse Cas9 antibody (Thermo Fisher Scientific, MA5-23519) as the primary antibody and goat anti-mouse antibody (LI-COR IRDye 680RD and 926-68070) as the secondary antibody.

### Off-target analysis in cultured cells

For comparison of off-target editing between plasmid transfection and PE-eVLPs, cells were seeded at a density of 30,000–35,000 cells per well in 48-well plates as described previously. After 1 day, plasmid transfection was performed as described previously and PE-eVLP transduction was performed by adding 10 μl of ultracentrifuge-concentrated PE-eVLPs to media containing target cells. A total of 3 days after treatment, cells were split into new 48-well plates to prevent cells from being overconfluent. A total of 7 days after treatment, genomic DNA was extracted from cells as described previously. Genomic DNAs were used for the amplification of the on-target *HEK4* locus, off-target site 1 and off-target site 3.

The off-target editing was analyzed as described previously^2^. Briefly, reads were aligned to reference off-target amplicons using CRISPResso2 with parameters ‘-q 30’, ‘discard_indel_reads TRUE’ and ‘-w 25’. Off-target reads were called as leniently as possible to capture all potential reverse transcription products at the Cas9 nick site. To assess potential pegRNA-mediated off-target editing, nucleotide sequence 3′ of the Cas9 nick site was compared to the 3′ DNA flap sequence encoded by the epegRNA reverse transcription template. The minimum sequence of the 3′ DNA flap that deviates from Cas9 nick site was designated as an off-target marker sequence. All reference-aligned reads that contain this off-target marker sequence were called as off-target reads and pegRNA-mediated off-target editing efficiency was calculated as the percentage of (reads containing the off-target marker sequence)/(the total number of reference-aligned reads). Frequency of insertions or deletions at the off-target Cas9 nick sites were quantified as a percentage of (discarded reads)/(the total reference-aligned reads). Total off-target editing is calculated as (pegRNA-mediated off-target editing frequency) + (indel frequency at the Cas9 nick site).

### Lentivirus production

Lentivirus used in this study was produced as described previously^44^. Briefly, HEK293T/17 (ATCC CRL-11268) cells were plated in T75 flasks (Corning; 353136) at a density of 5 × 10^6^ cells per flask in 10 ml of DMEM + 10% FBS medium. A total of 20–24 h after seeding, for production of lentivirus expressing GFP:KASH, plasmids expressing VSV-G (6,000 ng), psPAX2 (9,000 ng) and lenti-GFP:KASH (9,000 ng) were mixed in 1.5 ml of Opti-MEM and were incubated with FuGENE HD Transfection Reagent (Promega; E2312) following the manufacture’s protocol. The plasmid transfection mixture was added directly to the media of the cells. A total of 40–48 h after transfection, supernatants were collected and centrifuged at 500*g* for 5 min to remove the cell debris. Then the supernatant was filtered through 0.45-μm PVDF filter. Lentivirus was subsequently concentrated into 20% (w/v) sucrose in PBS cushion solution via ultracentrifugation as described above for eVLP production.

### Animals

Timed pregnant C57BL/6J mice for P0 studies were purchased from Charles River Laboratories (027). Retinal degeneration mouse models *rd6* (003684) and *rd12* (005379) were purchased from the Jackson Laboratory. All experiments involving live animals were approved by the Broad Institute Institutional Animal Care and Use Committee (D16-00903; 0048-04-15-2) and the University of California, Irvine Institutional Animal Care and Use Committee (D16-00259; AUP-21-096). Mouse housing facilities were maintained at 20–22 °C with 30–50% humidity, on a 12 h light/12 h dark cycle with ad libitum access to standard rodent diet and water. Animals were randomly assigned to various experimental groups.

### P0 ICV injections and tissue collection

P0 ICV injections were performed as previously described^25,44,82^. Briefly, syringes for microinjection were generated by pulling PCR Micropipettes (Drummond Scientific Company, 5-000-1001-X10) on the Sutter P1000 micropipette puller. Injection solution was made immediately before injection by mixing 4 μl of PE-eVLPs, 0.3 μl of VSV-G pseudotyped GFP:KASH lentivirus and 0.1 μl of Fast Green. A total of 4 μl injection solution (containing approximately 1.0 × 10^11^ eVLPs) was front-loaded to Drummond PCR pipettes. Neonatal mice were cryo-anesthetized on ice until they were unresponsive to bilateral toe pinch. Then 2 μl of injection solution was injected into each ventricle. Injection was verified by the spread of Fast Green via transillumination of the head. A total of 3 weeks after injection, mice were killed by CO_2_ asphyxiation. Brain tissues were collected by splitting the hemispheres along the sagittal plane.

### Nuclear isolation and sorting

Nuclei isolation was performed as previously described^25,44,82^. Briefly, collected brain hemispheres were transferred to the Dounce homogenizer (Sigma-Aldrich, D8938) along with 2 ml of EZ-PREP buffer (Sigma-Aldrich, NUC-101). Tissues were homogenized with 20 strokes with pestle A and 20 strokes with pestle B. The homogenates were combined with 2 ml of fresh EZ-PREP buffer and were centrifuged at 500*g* for 5 min. Supernatant was decanted and the nuclei pellet was washed by resuspending in 4 ml of ice-cold Nuclei Suspension Buffer (100 μg ml^−1^ BSA and 3.33 μM Vybrant DyeCycle Ruby (Thermo Fisher, V10309) in PBS). The mixture was centrifuged again at 500*g* for 5 min. Following two rounds of wash total, the pellet was resuspended in 3 ml of nuclear resuspension buffer and was filtered through 35-μm cell strainer. The isolated nuclei were flow-sorted using the Sony MA900 Cell Sorter (Sony Biotechnology) at the Broad Institute flow cytometry core using MA900 Cell Sorter software v3.1. See Extended Data Fig. 7 for a representative example of fluorescence-activated cell sorting gating. Nuclei were sorted into DNAdvance lysis buffer (Beckman Coulter, A48705) supplemented with 25 mM dithiothreitol and Proteinase K (Thermo Fisher). The genomic DNA was subsequently purified following the manufacturer’s protocol using DNAdvance kit (Beckman Coulter, A48705). For neuron-specific sorting, nuclei isolation was performed as described above. After the first centrifugation step, nuclei were washed with 4 ml of PBS + BSA (100 μg ml^−1^). Following centrifugation and decanting supernatant, nuclei were resuspended with 1 ml of PBS + BSA (100 μg ml^−1^) and 1 μl of anti-NeuN antibody (Abcam, ab190565) was added. Following incubation at 4 °C for 45 min in the dark with rocking, the mixture was centrifuged at 500*g* for 5 min. The supernatant was decanted and the pellet was washed twice with 1 ml of PBS supplemented with 100 μg ml^−1^ BSA and 3 μM DAPI (Thermo Fisher, D1306). The stained nuclei were then flow-sorted and processed as described above.

### Subretinal injection

The injection mix for subretinal injection was prepared immediately before injection by mixing 15–20 μl of PE-eVLP with 0.3 μl AAV–GFP (Addgene, 105530-AAV1). Mice were anesthetized by intraperitoneal injection of a cocktail consisting of 20 mg ml^−1^ ketamine and 1.75 mg ml^−1^ xylazine in PBS at a dose of 0.1 ml per 20 g body weight, and their pupils were dilated with topical administration of 1% tropicamide ophthalmic solution (Akorn, 17478-102-12) and 10% phenylephrine (Valeant, 42702-0103-05). Subretinal injections were performed under an ophthalmic surgical microscope (Zeiss). The corneas were hydrated with the application of GenTeal Severe Lubricant Eye Gel (0.3% hypromellose, Alcon). An incision was made through the cornea adjacent to the limbus at the nasal side using a 27-gauge needle. A 34-gauge blunt-end needle (World Precision Instruments, NF34BL-2) connected to an RPE-KIT (World Precision Instruments, RPE-KIT) by SilFlex tubing (World Precision Instruments, SILFLEX-2) was inserted through the corneal incision while avoiding the lens and advanced through the retina. Each mouse was injected with 1 μl of PE-eVLP (containing approximately 4.2 × 10^10^ eVLPs) + AAV1–GFP (used to confirm injection efficiency) mixture per eye. After injections, the gel was reapplied, anesthesia was reversed with intraperitoneal atipamezole (2.5 mg kg^−1^; MWI Animal Health, 032800) and mice were allowed to recover on a heat pad. Two weeks after injection, GFP signal was assessed by scanning laser ophthalmoscopy as a marker for injection efficiency and retinas that showed >80% GFP^+^ were collected for downstream analysis.

### Electroretinography

Before recording, mice were dark adapted for 24 h overnight. Under a safety light, mice were anesthetized by intraperitoneal injection of a cocktail consisting of 20 mg ml^−1^ ketamine and 1.75 mg ml^−1^ xylazine in PBS at a dose of 0.1 ml per 20 g body weight, and their pupils were dilated with topical administration of 1% tropicamide ophthalmic solution (Akorn, 17478-102-12) and 10% phenylephrine (Valeant, 42702-0103-05). The corneas were hydrated with the application of GenTeal Severe Lubricant Eye Gel (0.3% hypromellose, Alcon). The mouse was placed on a heated Diagnosys Celeris rodent ERG device (Diagnosys LCC). Ocular electrodes were placed on the corneas, the reference electrode was positioned subdermally between the ears, and the ground electrode was placed in the rear leg. The eyes were stimulated with a green light (peak emission 544 nm, bandwidth ∼160 nm) stimulus of −0.3 log (candela second per meter squared (cd s m^−2^)). The responses for ten stimuli with an inter-stimulus interval of 10 s were averaged together, and the a- and b-wave amplitudes were acquired from the averaged ERG waveform. The ERGs were recorded with the Celeris rodent electrophysiology system (Diagnosys LLC) and analyzed with Espion V6 software (Diagnosys LLC).

### RPE dissociation and genomic DNA and RNA preparation

Under a light microscope, mouse eyes were dissected to separate the posterior eyecup (containing RPE, choroid and sclera) from the retina and anterior segments. Each posterior eyecup was immediately immersed in PBS. RPE, choroid and scleral cells were detached in PBS from the posterior eyecup by gentle pipetting, followed by a removal of the remaining posterior eyecup. Cells from *rd6* mice were then processed for genomic DNA using the DNeasy Blood & Tissue Kit (Qiagen, 69504) and cells from *rd12* mice were processed with the AllPrep DNA/RNA Micro Kit (Qiagen, 80284).

### Western blot analysis of mouse RPE tissue extracts

To prepare the protein lysate from the mouse RPE tissue, the dissected mouse eyecup, consisting of RPE, choroid and sclera, was transferred to a microcentrifuge tube containing 40 μl of RIPA buffer with protease inhibitors and homogenized with a motorized grinder (Fisher Scientific, K749540-0000), incubated on ice for 20 min and then centrifuged for 20 min at 21,000*g* at 4 °C. The resulting supernatant was precleared with Dynabeads Protein G (Thermo Fisher, 10003D) to remove immunoglobulin contaminants from the blood before gel loading. A total of 10 μl of RPE lysates premixed with NuPAGE LDS Sample Buffer (Thermo Fisher, NP0007) and NuPAGE Sample Reducing Agent (Thermo Fisher, NP0004), and denatured at 70 °C for 10 min, was loaded into each well of a NuPAGE 4–12% Bis-Tris gel (Thermo Fisher, NP0321BOX), separated for 1 h at 130 V and transferred onto a PVDF membrane (Millipore, IPVH00010). After 1 h blocking in 5% (w/v) nonfat milk in PBS containing 0.1% (v/v) Tween-20 (PBS-T), the membrane was incubated with primary antibody, goat anti-mouse MFRP monoclonal antibody (1:1,000; R&D Systems, AF3445) or mouse anti-mouse RPE65 (1:1,000; in-house production^83^) diluted in 1% (w/v) nonfat milk in PBS-T overnight at 4 °C. After overnight incubation, membranes were washed three times with PBS-T for 5 min each and then incubated with donkey anti-goat IgG–horseradish peroxidase (HRP) antibody (1:10,000; Abcam, ab97110) or goat anti-mouse IgG–HRP antibody (1:5,000; Cell Signaling Technology, 7076S) for 1 h at room temperature. After washing the membrane three times with PBS-T for 5 min each, protein bands were visualized after exposure to SuperSignal West Pico Plus Chemiluminescent substrate (Thermo Fisher; 34577). Membranes were stripped (Thermo Fisher, 21059), reblocked and reprobed for β-actin expression using rabbit anti-β-actin polyclonal antibody (1:1,000; Cell Signaling Technology, 4970S), following the same protocol. The corresponding secondary antibody was goat anti-rabbit IgG–HRP antibody (1:5,000; Cell Signaling Technology, 7074S).

### Immunohistochemistry of RPE flatmounts and cryosections

Mouse eyes were enucleated and fixed with 4% paraformaldehyde in PBS for 20 min at room temperature and washed three times in PBS for 5 min each. To make RPE flatmounts, the anterior segment and retina were removed from the posterior eyecup under a dissecting microscope, and four radial cuts were made toward the optic nerve to flatten the eyecup into an RPE flatmount. Samples were permeabilized and blocked in 0.1% Triton X-100 (Sigma-Aldrich, T8532) with 3% normal donkey serum (NDS) in PBS for 30 min and incubated with the appropriate primary antibody in PBS, 0.1% Triton X-100 and 3% NDS, including goat anti-MFRP antibody (1:100; R&D Systems, AF3445) and rabbit anti-ZO-1 polyclonal antibody (1:100; Invitrogen, 61-7300) overnight at 4 °C. The next day, samples were washed three times in PBS for 5 min each and then incubated with the appropriate secondary antibody in PBS + 0.1% Triton X-100 and 3% NDS, including Alexa Fluor 594-conjugated donkey anti-rabbit IgG (1:200; Thermo Fisher, A21207) and Alexa Fluor 647-conjugated donkey anti-goat IgG (1:200; Thermo Fisher, A32849) for 2 h at room temperature in the dark. Cryosection samples were incubated in 1 ml DAPI (Thermo Fisher, 62248) in PBS for 10 min. Samples were washed three times in PBS for 5 min each. The samples were then mounted with VECTASHIELD HardSet Antifade Mounting Medium (Vector Labs H-1400-10) and imaged on a Keyence BZ-X800 All-in-One fluorescence microscope.

### CIRCLE-seq nomination of off-target sites for the *rd6* and *rd12* models

CIRCLE-seq off-target editing analysis was performed as previously described^76,84^. Genomic DNA from *rd6* mouse liver was isolated using Gentra Puregene Kit (Qiagen, 158845) following the manufacturer’s instructions. Purified genomic DNA was sheared with a Covaris S2 instrument to an average length of 300 bp. The fragmented DNA was end repaired, A-tailed and ligated to a uracil-containing stem–loop adaptor, using the KAPA HTP Library Preparation Kit, PCR Free (KAPA Biosystems, KK8235). Adaptor-ligated DNA was treated with Lambda Exonuclease (New England Biolabs, M0262) and *E. coli* Exonuclease I (New England Biolabs, M0293) and then with USER enzyme (New England Biolabs, M5505) and T4 poly-nucleotide kinase (New England Biolabs, M0201). Intramolecular circularization of the DNA was performed with T4 DNA ligase (New England Biolabs, M0202) and residual linear DNA was degraded by Plasmid-Safe ATP-dependent DNase (Lucigen, E3110). Synthetic guide RNAs were ordered from IDT with standard 2′-*O*-methyl modification at first three and last three bases. The synthetic guide RNAs were resuspended to 9 µM in nuclease-free water, denatured at 90 °C for 5 min and slowly annealed at 0.1 °C s^−1^ to 25 °C. In vitro cleavage reactions were performed with 125 ng Plasmid-Safe-treated circularized DNA, 90 nM Cas9 nuclease protein (New England Biolabs, M0386) and 270 nM synthetic guide RNA in a 50 µl volume for 1 h. Cleaved products were treated with proteinase K as described^84^, A-tailed, ligated with a hairpin adaptor (New England Biolabs, E7600S), treated with USER enzyme (New England Biolabs, M5505) and amplified by PCR with barcoded universal primers (New England Biolabs, E7600S) using Kapa HiFi Polymerase (KAPA Biosystems, KK4824). Libraries were sequenced with 150-bp/150-bp paired-end reads with an Illumina MiSeq instrument. CIRCLE-seq data analyses were performed using open-source CIRCLE-seq analysis software and default recommended parameters^85^. The top ten nominated off-target sites for epegRNA used for the *rd6* and *rd12* models were analyzed by HTS from the RPE tissue of untreated or v3 PE3b-eVLP-treated mice. Off-target editing for epegRNA-associated off-target sites was analyzed, as described above, as (pegRNA-mediated off-target editing frequency) + (indel frequency at the Cas9 nick site). Insertions or deletions at ngRNA-associated off-target sites were analyzed as a percentage of discarded reads divided by the total reference-aligned reads. Top ten CIRCLE-seq nominated off-target sites are listed in Supplementary Table 6 (*rd6* model) and Supplementary Table 7 (*rd12* model).

### Statistics and reproducibility

Data are presented as mean and standard error of the mean (s.e.m.). Comparisons of different versions of PE-eVLPs were made with eVLPs produced and transduced in parallel in one large experiment. Biological replicates were obtained by treating three independently maintained cell line splits (aliquots) for cell culture studies, or three or more animals for in vivo studies, with a single batch of PE-eVLPs. Low batch-to-batch variability for different PE-eVLP batches is shown in Extended Data Fig. 5b. The sample size and the statistical tests used for each experiment are described in the figure legends. No statistical methods were used to predetermine sample size. Statistical analysis was performed using GraphPad Prism software.

### Reporting summary

Further information on research design is available in the Nature Portfolio Reporting Summary linked to this article.

## Online content

Any methods, additional references, Nature Portfolio reporting summaries, source data, extended data, supplementary information, acknowledgements, peer review information; details of author contributions and competing interests; and statements of data and code availability are available at 10.1038/s41587-023-02078-y.

## Supplementary information

Supplementary Information Supplementary Figs. 1–2, Notes 1–3, sequences and Tables 1–7.

Reporting Summary

Supplementary Tables Supplementary Tables 1–7.

## Source data

Source Data Fig. 5 Unprocessed western blots for Fig. 5d,k.

## Data Availability

HTS data files were deposited to the NCBI Sequence Read Archive database under accession codes PRJNA980181 (ref. ^86^). DNA sequences of the PE-eVLP architecture are provided in Supplementary Information. The following key plasmids from this work are deposited to Addgene for distribution: Gag–MCP–Pol (Addgene #211370), Gag–PE (Addgene #211371), MS2-epegRNA–Dnmt1 (Addgene #211372), Gag–COM–Pol (Addgene #211373), Gag–PE3–Pol (#211374), P4–PE (#211375), COM-epegRNA–Dnmt1 (#211376). Other plasmids and raw data are available from the corresponding author on request. Unmodified image of the western blots shown in Fig. 5d,k are provided as Source data. Source data are provided with this paper.

## Code availability

The code used for analysis of HTS data is available at https://github.com/pinellolab/CRISPResso2. The code used for analysis for CIRCLE-seq data is available at https://github.com/tsailabSJ/circleseq.
