# Supplementary material for: Engineered virus-like particles for transient delivery of prime editor ribonucleoprotein complexes in vivo
Source: Nat Biotechnol. 2024 Jan 8;42(10):1526–37. doi: 10.1038/s41587-023-02078-y (PMC11228131; doi:10.1038/s41587-023-02078-y)
Supplement: Supplementary file 1 — Supplementary Figs. 1–2, Notes 1–3, sequences and Tables 1–7. [file 41587_2023_2078_MOESM1_ESM.pdf]

nature biotechnology

Article

https://doi.org/10.1038/s41587-023-02078-y

Engineered virus-like particles for transient delivery of prime editor ribonucleoprotein complexes in vivo

In the format provided by the authors and unedited

Supplementary Information

Supplementary Figure 1. Data showing all PE-eVLPs produced and tested in parallel for Fig. 1.

Supplementary Figure 2. Data showing all PE-eVLPs produced and tested in parallel for Fig 2.

Supplementary Note 1. epegRNAs have reduced binding affinity to Cas9 compared to sgRNAs.

Supplementary Note 2. MCP insertion does not impair eVLP production.

Supplementary Note 3. Western blot analysis of protein content in the producer cell lysates and successive generations of PE-eVLPs.

Supplementary Sequences. Sequences of key eVLP plasmids used in this study.

- **Supplementary Sequence 1.** PEmax with a 6-amino acid deletion at the C-terminus of MMLV RT
- **Supplementary Sequence 2.** Gag-PE in v3 PE3-eVLPs
- **Supplementary Sequence 3.** Gag-MCP-pol in v3 PE-eVLPs
- **Supplementary Sequence 4.** P4-PE in v3b PE-eVLPs
- **Supplementary Sequence 5.** Gag-P3-pol in v3b PE-eVLPs
- **Supplementary Sequence 6.** Gag-COM-pol in v3b PE-eVLPs

Supplementary Tables (provided as a separate file):

- **Supplementary Table 1.** Sequences of pegRNAs, epegRNAs, and ngRNAs used in this study.
- **Supplementary Table 2.** Sequences of primers used for genomic DNA amplification and amplicons analyzed with high-throughput sequencing.
- **Supplementary Table 3.** Sequences of primers used for RT-qPCR.
- **Supplementary Table 4.** epegRNA and ngRNA optimization for *rd6* model correction*.*
- **Supplementary Table 5.** epegRNA and ngRNA optimization for *rd12* model correction*.*
- **Supplementary Table 6.** Sequences of top 10 CIRCLE-seq nominated OT sites for *rd6.*
- **Supplementary Table 7.** Sequences of top 10 CIRCLE-seq nominated OT sites for *rd12.*

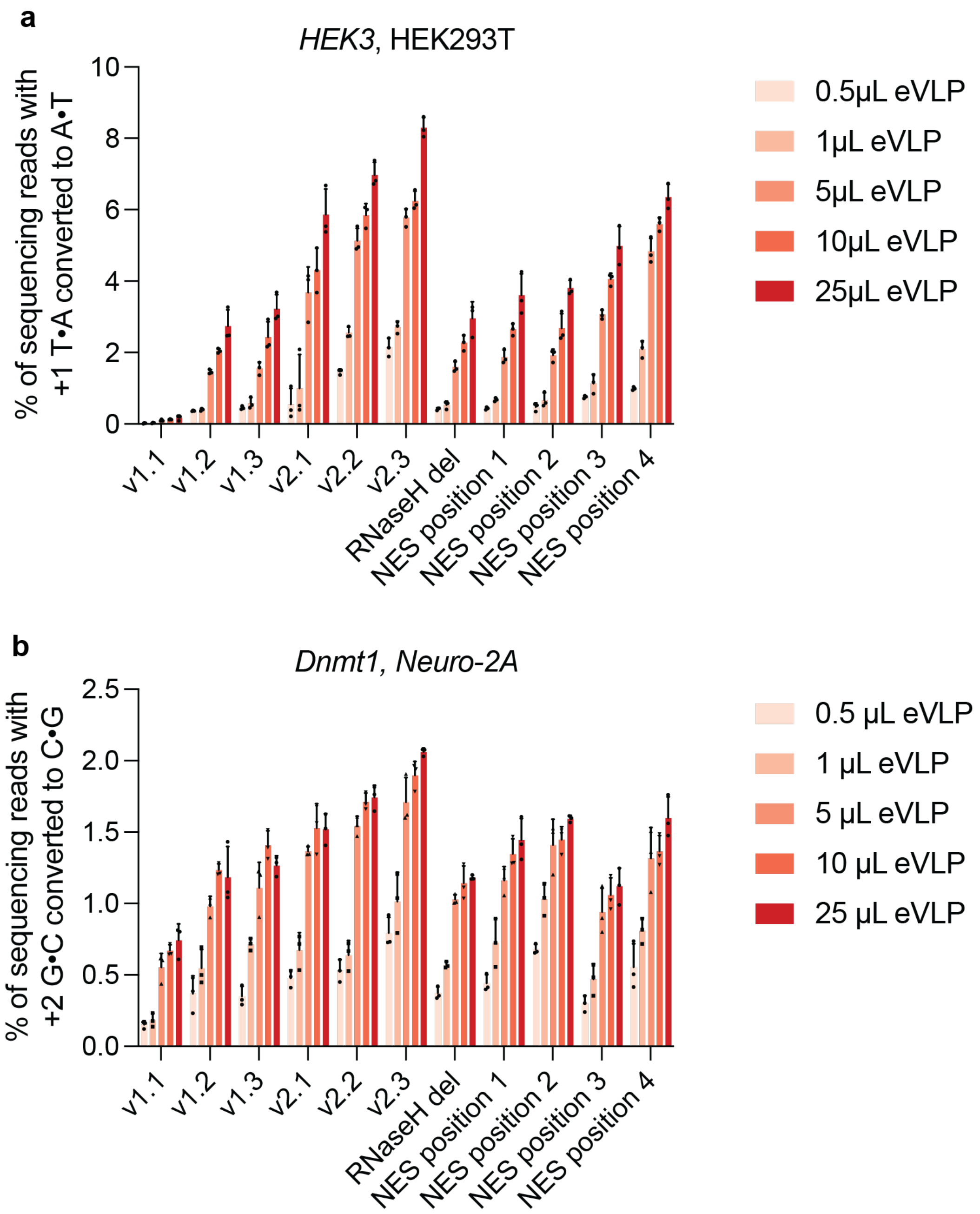

Supplementary Figure 1. Data showing all PE-eVLPs produced and tested in parallel for Fig. 1. a, Prime editing efficiencies of “v1.1”, “v1.2”, “v1.3”, “v2.1”, “v2.2”, “v2.3”, “RNaseH del”, “NES position 1”, “NES position 2”, “NES position 3”, “NES position 4” PE-eVLPs at *HEK3* locus in HEK293T cells. Fig 1b, 1d, 1f and 1g show a subset of data from this experiment. Fig. 1b shows results of “v1.1”, “v1.2” and “v1.3” PE-eVLPs fitted to four-parameter logistic curves using nonlinear regression. Fig. 1d shows results of “v1.3”, “RNaseH del”, and “v2.1” PE-eVLPs fitted to four-parameter logistic curves using nonlinear regression. Fig. 1f shows results from “v2.1”, “NES position 1”, “NES position 2”, “NES

position 3”, “NES position 4”, and “v2.3” PE-eVLPs fitted to four-parameter logistic curves using nonlinear regression. Fig. 1g shows results of “v1.1”, “v1.3”, “v2.1”, “v2.2”, and “v2.3” PE-eVLPs fitted to four-parameter logistic curves using nonlinear regression. **b**, Prime editing efficiencies of “v1.1”, “v1.2”, “v1.3”, “v2.1”, “v2.2”, “v2.3”, “RNaseH del”, “NES position 1”, “NES position 2”, “NES position 3”, “NES position 4” PE-eVLPs at *Dnmt1* locus in Neuro-2A cells. Fig 1b, 1d and 1g show a subset of data from this experiment. Fig. 1b shows results of “v1.1”, “v1.2” and “v1.3” PE-eVLPs fitted to four-parameter logistic curves using nonlinear regression. Fig. 1d shows results of “v1.3”, “RNaseH del”, and “v2.1” PE-eVLPs fitted to four-parameter logistic curves using nonlinear regression. Fig. 1g shows results of “v1.1”, “v1.3”, “v2.1”, “v2.2”, and “v2.3” PE-eVLPs fitted to four-parameter logistic curves using nonlinear regression. Values shown in all graphs represent the average prime editing efficiency of three biological replicates and error bars represent the standard deviation.

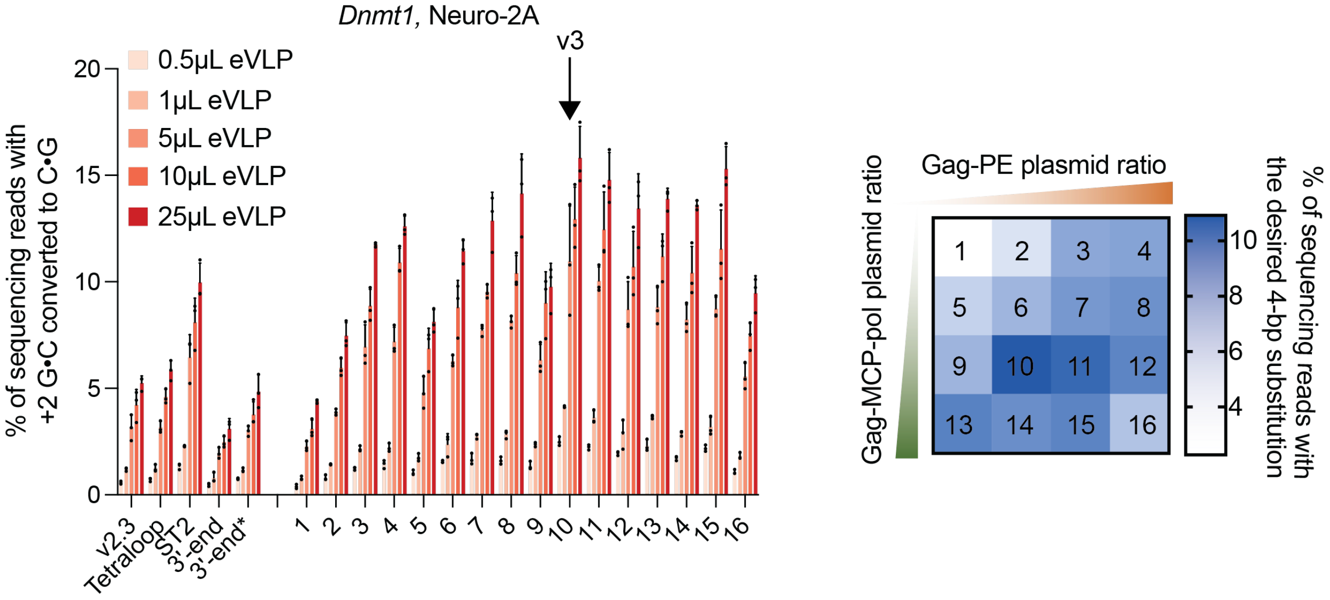

Supplementary Figure 2. Data showing all PE-eVLPs produced and tested in parallel for Fig. 2. Prime editing efficiencies of "v2.3", "Tetraloop", "ST2", "3'-end", "3'-end*" PE-eVLPs, and PE-eVLPs 1-16 tested for stoichiometry optimization in Fig. 2d (number ID is shown on the right to match Fig. 2d and Supplementary Fig 2), at *Dnmt1* locus in Neuro-2A cells. Figs. 2c, 2d, and 2e show a subset of data from this experiment. Fig. 2c show prime editing efficiencies of "v2.3", "Tetraloop", "ST2", "3'-end", and "3'-end*" PE-eVLPs fitted to four-parameter logistic curves using nonlinear regression. Fig. 2d shows prime editing efficiencies of PE-eVLPs 1-16 at 5 μL dose, shown as heatmap. Fig. 2e shows prime editing efficiencies of "v2.3" PE-eVLP and PE-eVLP 10 (which is v3 PE-eVLPs) fitted to four-parameter logistic curves using nonlinear regression. Values shown in all graphs represent the average prime editing efficiency of three biological replicates and error bars represent the standard deviation.

Supplementary Note 1. epegRNAs have reduced binding affinity to Cas9 compared to sgRNAs.

We previously observed that the apparent affinity of Cas9 for sgRNAs, pegRNAs, and epegRNAs decreases in that order[1] . The reduced binding affinity can be attributed to the addition of a 3’ extension in pegRNAs and an additional structured 3’ end motif in epegRNAs that adds steric hindrance to the Cas9 binding. As a result, while the enhanced stability of epegRNAs improves prime editing efficiencies compared to pegRNAs, epegRNAs might be improved further by enhancing their binding affinity to Cas9.

Supplementary Note 2. MCP insertion does not impair eVLP production.

To first assess whether incorporation of an MCP domain into gag-pol would disrupt the formation of eVLPs, we transfected the producer cells with a 3:1 ratio of wild-type gag-pol plasmid to gag-MCP-pol plasmid, as well as the rest of the components for PE-eVLP production. Prime editing efficiency of a 4-bp substitution at *Dnmt1* locus in N2A cells was maintained upon the addition of gag-MCP-pol, indicating that the MCP fusion did not significantly impair eVLP production (Extended Data Fig. 2c).

Supplementary Note 3. Western blot analysis of protein content in the producer cell lysates and successive generations of PE-eVLPs.

To assess whether the P4-PE construct indeed improves prime editor expression in producer cells, we transfected eVLP producer cells with gag-pol and cargo fusion plasmids (gag-PE or P4-PE), and performed western blots of producer cell lysates 48 h after transfection. We indeed observed that P4-PE protein levels were substantially higher than gag–PE levels in producer cells (Extended Data Fig. 4a), consistent with our hypothesis that the smaller P4-PE fusion protein improved protein production levels. We also performed western blot analysis on lysed v1.3, v2.3, v3 and v3b PE-eVLPs to assess the amount of cargo packaged in each generation of PE-eVLPs. Consistent with the PE-eVLP protein quantification results from ELISA, we observed improvement in the prime editor protein packaging over successive generations of PE-eVLPs from v1.3 to v3 (Extended Data Fig. 4d). Comparing v3 and v3b PE-eVLPs, we did not observe notable differences in the prime editor protein packaging, consistent with the comparable editing efficiencies observed for v3 and v3b PE-eVLPs at multiple target sites tested (Fig. 3e). The advantage in producer cell expression levels offered by the v3b PE-eVLP architecture may be partially counteracted by less efficient cargo recruitment via P3-P4 coiled-coil peptide interaction compared to the direct gag-PE fusion in v3 PE-eVLPs. Nonetheless, these findings confirmed that the v3b PE-eVLP architecture improves cargo expression in the producer cells and mediates cargo packaging into eVLPs with comparable efficiency to the v3 PE-eVLP architecture.

Supplementary Sequence 1. PEmax with a 6-amino acid deletion at the C-terminus of MMLV RT.

Key: NLS-Cas9 nickase (R221K, N393K, H840A)-linker-MMLV RT (6 amino acid del)-NLS-cmycNLS

AAACGGACAGCCGACGGAAGCGAGTTCGAGTCACCAAAGAAGAAGCGGAAAGTCGACAAGAAGTACAGCA
TCGGCCTGGACATCGGCACCAACTCTGTGGGCTGGGCCGTGATCACCGACGAGTACAAGGTGCCCAGCAA
GAAATTCAAGGTGCTGGGCAACACCGACCGGCACAGCATCAAGAAGAACCTGATCGGAGCCCTGCTGTTC
GACAGCGGCGAAACAGCCGAGGCCACCCGGCTGAAGAGAACCGCCAGAAGAAGATACACCAGACGGAAG
AACCGGATCTGCTATCTGCAAGAGATCTTCAGCAACGAGATGGCCAAGGTGGACGACAGCTTCTTCCACAG
ACTGGAAGAGTCCTTCCTGGTGGAAGAGGATAAGAAGCACGAGCGGCACCCCATCTTCGGCAACATCGTG
GACGAGGTGGCCTACCACGAGAAGTACCCCACCATCTACCACCTGAGAAAGAAACTGGTGGACAGCACCG
ACAAGGCCGACCTGCGGCTGATCTATCTGGCCCTGGCCCACATGATCAAGTTCCGGGGCCACTTCCTGATC
GAGGGCGACCTGAACCCCGACAACAGCGACGTGGACAAGCTGTTCATCCAGCTGGTGCAGACCTACAACC
AGCTGTTCGAGGAAAACCCCATCAACGCCAGCGGCGTGGACGCCAAGGCCATCCTGTCTGCCAGACTGAG
CAAGAGCAGAAAGCTGGAAAATCTGATCGCCCAGCTGCCCGGCGAGAAGAAGAATGGCCTGTTCGGAAAC
CTGATTGCCCTGAGCCTGGGCCTGACCCCCAACTTCAAGAGCAACTTCGACCTGGCCGAGGATGCCAAAC
TGCAGCTGAGCAAGGACACCTACGACGACGACCTGGACAACCTGCTGGCCCAGATCGGCGACCAGTACGC
CGACCTGTTTCTGGCCGCCAAGAACCTGTCCGACGCCATCCTGCTGAGCGACATCCTGAGAGTGAACACC
GAGATCACCAAGGCCCCCCTGAGCGCCTCTATGATCAAGAGATACGACGAGCACCACCAGGACCTGACCCT
GCTGAAAGCTCTCGTGCGGCAGCAGCTGCCTGAGAAGTACAAAGAGATTTTCTTCGACCAGAGCAAGAACG
GCTACGCCGGCTACATTGACGGCGGAGCCAGCCAGGAAGAGTTCTACAAGTTCATCAAGCCCATCCTGGAA
AAGATGGACGGCACCGAGGAACTGCTCGTGAAGCTGAAGAGAGAGGACCTGCTGCGGAAGCAGCGGACC
TTCGACAACGGCAGCATCCCCCACCAGATCCACCTGGGAGAGCTGCACGCCATTCTGCGGCGGCAGGAAG
ATTTTTACCCATTCCTGAAGGACAACCGGGAAAAGATCGAGAAGATCCTGACCTTCCGCATCCCCTACTACG
TGGGCCCTCTGGCCAGGGGAAACAGCAGATTCGCCTGGATGACCAGAAAGAGCGAGGAAACCATCACCCC
CTGGAACTTCGAGGAAGTGGTGGACAAGGGCGCTTCCGCCCAGAGCTTCATCGAGCGGATGACCAACTTC
GATAAGAACCTGCCCAACGAGAAGGTGCTGCCCAAGCACAGCCTGCTGTACGAGTACTTCACCGTGTATAA
CGAGCTGACCAAAGTGAAATACGTGACCGAGGGAATGAGAAAGCCCGCCTTCCTGAGCGGCGAGCAGAAA
AAGGCCATCGTGGACCTGCTGTTCAAGACCAACCGGAAAGTGACCGTGAAGCAGCTGAAAGAGGACTACT
TCAAGAAAATCGAGTGCTTCGACTCCGTGGAAATCTCCGGCGTGGAAGATCGGTTCAACGCCTCCCTGGGC
ACATACCACGATCTGCTGAAAATTATCAAGGACAAGGACTTCCTGGACAATGAGGAAAACGAGGACATTCTG
GAAGATATCGTGCTGACCCTGACACTGTTTGAGGACAGAGAGATGATCGAGGAACGGCTGAAAACCTATGC
CCACCTGTTCGACGACAAAGTGATGAAGCAGCTGAAGCGGCGGAGATACACCGGCTGGGGCAGGCTGAG
CCGGAAGCTGATCAACGGCATCCGGGACAAGCAGTCCGGCAAGACAATCCTGGATTTCCTGAAGTCCGAC
GGCTTCGCCAACAGAAACTTCATGCAGCTGATCCACGACGACAGCCTGACCTTTAAAGAGGACATCCAGAA
AGCCCAGGTGTCCGGCCAGGGCGATAGCCTGCACGAGCACATTGCCAATCTGGCCGGCAGCCCCGCCATT
AAGAAGGGCATCCTGCAGACAGTGAAGGTGGTGGACGAGCTCGTGAAAGTGATGGGCCGGCACAAGCCC

GAGAACATCGTGATCGAAATGGCCAGAGAGAACCAGACCACCCAGAAGGGACAGAAGAACAGCCGCGAGA
GAATGAAGCGGATCGAAGAGGGCATCAAAGAGCTGGGCAGCCAGATCCTGAAAGAACACCCCGTGGAAAA
CACCCAGCTGCAGAACGAGAAGCTGTACCTGTACTACCTGCAGAATGGGCGGGATATGTACGTGGACCAGG
AACTGGACATCAACCGGCTGTCCGACTACGATGTGGACGCTATCGTGCCTCAGAGCTTTCTGAAGGACGAC
TCCATCGACAACAAGGTGCTGACCAGAAGCGACAAGAACCGGGGCAAGAGCGACAACGTGCCCTCCGAAG
AGGTCGTGAAGAAGATGAAGAACTACTGGCGGCAGCTGCTGAACGCCAAGCTGATTACCCAGAGAAAGTTC
GACAATCTGACCAAGGCCGAGAGAGGCGGCCTGAGCGAACTGGATAAGGCCGGCTTCATCAAGAGACAGC
TGGTGGAAACCCGGCAGATCACAAAGCACGTGGCACAGATCCTGGACTCCCGGATGAACACTAAGTACGAC
GAGAATGACAAGCTGATCCGGGAAGTGAAAGTGATCACCCTGAAGTCCAAGCTGGTGTCCGATTTCCGGAA
GGATTTCCAGTTTTACAAAGTGCGCGAGATCAACAACTACCACCACGCCCACGACGCCTACCTGAACGCCG
TCGTGGGAACCGCCCTGATCAAAAAGTACCCTAAGCTGGAAAGCGAGTTCGTGTACGGCGACTACAAGGTG
TACGACGTGCGGAAGATGATCGCCAAGAGCGAGCAGGAAATCGGCAAGGCTACCGCCAAGTACTTCTTCTA
CAGCAACATCATGAACTTTTTCAAGACCGAGATTACCCTGGCCAACGGCGAGATCCGGAAGCGGCCTCTGA
TCGAGACAAACGGCGAAACCGGGGAGATCGTGTGGGATAAGGGCCGGGATTTTGCCACCGTGCGGAAAGT
GCTGAGCATGCCCCAAGTGAATATCGTGAAAAAGACCGAGGTGCAGACAGGCGGCTTCAGCAAAGAGTCTA
TCCTGCCCAAGAGGAACAGCGATAAGCTGATCGCCAGAAAGAAGGACTGGGACCCTAAGAAGTACGGCGG
CTTCGACAGCCCCACCGTGGCCTATTCTGTGCTGGTGGTGGCCAAAGTGGAAAAGGGCAAGTCCAAGAAA
CTGAAGAGTGTGAAAGAGCTGCTGGGGATCACCATCATGGAAAGAAGCAGCTTCGAGAAGAATCCCATCGA
CTTTCTGGAAGCCAAGGGCTACAAAGAAGTGAAAAAGGACCTGATCATCAAGCTGCCTAAGTACTCCCTGTT
CGAGCTGGAAAACGGCCGGAAGAGAATGCTGGCCTCTGCCGGCGAACTGCAGAAGGGAAACGAACTGGC
CCTGCCCTCCAAATATGTGAACTTCCTGTACCTGGCCAGCCACTATGAGAAGCTGAAGGGCTCCCCCGAGG
ATAATGAGCAGAAACAGCTGTTTGTGGAACAGCACAAGCACTACCTGGACGAGATCATCGAGCAGATCAGC
GAGTTCTCCAAGAGAGTGATCCTGGCCGACGCTAATCTGGACAAAGTGCTGTCCGCCTACAACAAGCACCG
GGATAAGCCCATCAGAGAGCAGGCCGAGAATATCATCCACCTGTTTACCCTGACCAATCTGGGAGCCCCTG
CCGCCTTCAAGTACTTTGACACCACCATCGACCGGAAGAGGTACACCAGCACCAAAGAGGTGCTGGACGC
CACCCTGATCCACCAGAGCATCACCGGCCTGTACGAGACACGGATCGACCTGTCTCAGCTGGGAGGTGAC
TCCGGCGGAAGCTCTGGTGGCAGCAAGCGGACCGCCGACGGCTCTGAATTCGAGAGCCCTAAGAAGAAAA
GAAAGGTGAGCGGAGGCTCTAGCGGCGGAAGCACCCTGAACATTGAAGACGAGTATAGACTGCATGAAACA
AGCAAGGAACCCGACGTGTCCCTGGGCTCCACCTGGCTGTCCGACTTTCCCCAGGCCTGGGCCGAGACA
GGAGGAATGGGCCTGGCCGTGCGGCAGGCACCCCTGATCATCCCTCTGAAGGCCACCTCTACACCCGTGA
GCATCAAGCAGTACCCTATGTCTCAGGAGGCCAGACTGGGCATCAAGCCTCACATCCAGAGGCTGCTGGAC
CAGGGCATCCTGGTGCCATGCCAGAGCCCCTGGAACACACCACTGCTGCCCGTGAAGAAGCCAGGCACCA
ATGACTATAGACCCGTGCAGGATCTGAGAGAGGTGAACAAGAGGGTGGAGGATATCCACCCCACCGTGCCC
AACCCTTACAATCTGCTGTCCGGCCTGCCCCCTTCTCACCAGTGGTATACAGTGCTGGACCTGAAGGATGC
CTTCTTTTGTCTGAGACTGCACCCTACCAGCCAGCCACTGTTCGCCTTTGAGTGGAGGGACCCTGAGATGG
GCATCTCTGGCCAGCTGACCTGGACACGCCTGCCTCAGGGCTTCAAGAATAGCCCAACACTGTTTAACGAG
GCCCTGCACCGCGACCTGGCAGATTTCCGGATCCAGCACCCAGATCTGATCCTGCTGCAGTACGTGGACG
ATCTGCTGCTGGCCGCCACCAGCGAGCTGGATTGCCAGCAGGGAACACGCGCCCTGCTGCAGACCCTGG
GAAACCTGGGATATAGGGCATCCGCCAAGAAGGCCCAGATCTGTCAGAAGCAGGTGAAGTACCTGGGCTAT

CTGCTGAAGGAGGGCCAGAGATGGCTGACAGAGGCCAGGAAGGAGACAGTGATGGGCCAGCCAACACCC
AAGACCCCAAGACAGCTGAGGGAGTTCCTGGGCAAAGCAGGATTTTGCAGGCTGTTCATCCCAGGATTCGC
AGAGATGGCAGCACCTCTGTACCCACTGACCAAGCCGGGCACCCTGTTTAATTGGGGCCCTGACCAGCAG
AAGGCCTATCAGGAGATCAAGCAGGCCCTGCTGACAGCACCAGCCCTGGGCCTGCCAGACCTGACCAAGC
CTTTCGAGCTGTTTGTGGATGAGAAGCAGGGCTACGCCAAGGGCGTGCTGACCCAGAAGCTGGGACCATG
GAGACGGCCCGTGGCCTATCTGTCCAAGAAGCTGGACCCAGTGGCAGCAGGATGGCCACCATGCCTGAGG
ATGGTGGCAGCAATCGCCGTGCTGACAAAGGATGCCGGCAAGCTGACCATGGGACAGCCACTGGTCATCC
TGGCACCACACGCAGTGGAGGCCCTGGTGAAGCAGCCTCCAGATCGCTGGCTGTCTAACGCCCGGATGAC
ACACTACCAGGCCCTGCTGCTGGACACCGATCGCGTGCAGTTTGGCCCTGTGGTGGCCCTGAATCCAGCC
ACCCTGCTGCCTCTGCCAGAGGAGGGCCTGCAGCACAACTGTCTGGACATCCTGGCAGAGGCACACGGAA
CAAGGCCAGACCTGACCGATCAGCCCCTGCCTGACGCCGATCACACATGGTATACCGATGGAAGCTCCCTG
CTGCAGGAGGGCCAGAGGAAGGCAGGAGCAGCAGTGACCACAGAGACAGAAGTGATCTGGGCCAAGGCC
CTGCCAGCAGGCACATCCGCCCAGCGGGCCGAGCTGATCGCCCTGACCCAGGCCCTGAAGATGGCCGAG
GGCAAGAAGCTGAACGTGTACACAGACTCCAGATATGCCTTCGCCACCGCACACATCCACGGAGAGATCTA
CAGGCGCCGGGGCTGGCTGACCTCTGAGGGCAAGGAGATCAAGAACAAGGATGAGATCCTGGCCCTGCT
GAAGGCCCTGTTTCTGCCCAAGCGGCTGAGCATCATCCACTGTCCTGGACACCAGAAGGGACACTCCGCC
GAGGCAAGGGGCAATCGGATGGCCGACCAGGCCGCCAGAAAGGCTGCTATTACTGAAACTCCCGACACTT
CCACTCTGCTGTCTGGCGGCTCAAAAAGAACCGCCGACGGCAGCGAATTCGAGTCTCCCAAGAAGAAGAG
GAAAGTCGGCTCTGGCCCTGCCGCTAAGAGAGTGAAGCTGGACTGA

Supplementary Sequence 2. Gag-PE in v3 PE3-eVLPs.

Key: Gag(MA+p12 domain)-3xNES-Gag(CA+NC domain)-linker-engineered protease cleavage site-linker- PEmax with 6 amino acid deletion at C-terminus of MMLV RT (NLS-Cas9 nickase (R221K, N393K, H840A)-linker-MMLV RT (6 amino acid del)-NLS-cmycNLS)

ATGGGCCAGACTGTTACCACTCCCTTAAGTTTGACCTTAGGTCACTGGAAAGATGTCGAGCGGATCGCTCAC
AACCAGTCGGTAGATGTCAAGAAGAGACGTTGGGTTACCTTCTGCTCTGCAGAATGGCCAACCTTTAACGTC
GGATGGCCGCGAGACGGCACCTTTAACCGAGACCTCATCACCCAGGTTAAGATCAAGGTCTTTTCACCTGG
CCCGCATGGACACCCAGACCAGGTCCCCTACATCGTGACCTGGGAAGCCTTGGCTTTTGACCCCCCTCCCT
GGGTCAAGCCCTTTGTACACCCTAAGCCTCCGCCTCCTCTTCCTCCATCCGCCCCGTCTCTCCCCCTTGAA
CCTCCTCGTTCGACCCCGCCTCGATCCTCCCTTTATCCAGCCCTCACTCCTTCTCTAGGCGCCAAACCTAAA
CCTCAAGTTCTTTCTGACAGTGGGGGGCCGCTCATCGACCTACTTACAGAAGACCCCCCGCCTTATAGGGA
CCCAAGACCACCCCCTTCCGACAGGGACGGAAATGGTGGAGAAGCGACCCCTGCGGGAGAGGCACCGGA
CCCCTCCCCAATGGCATCTCGCCTACGTGGGAGACGGGAGCCCCCTGTGGCCGACTCCACTTCTGGCGGC
TCACTTCAACTGCCTCCACTTGAAAGACTGACACTGGGATCATTACAATTACCTCCTTTAGAACGATTAACAC
TCGGTTCACTACAGCTTCCGCCTCTTGAGAGATTGACATTAAGTGGTGGATCTACCTCGCAGGCATTCCCCC
TCCGCGCAGGAGGAAACGGACAGCTTCAATACTGGCCGTTCTCCTCTTCTGACCTTTACAACTGGAAAAATA
ATAACCCTTCTTTTTCTGAAGATCCAGGTAAACTGACAGCTCTGATCGAGTCTGTCCTCATCACCCATCAGCC
CACCTGGGACGACTGTCAGCAGCTGTTGGGGACTCTGCTGACCGGAGAAGAAAAACAACGGGTGCTCTTA
GAGGCTAGAAAGGCGGTGCGGGGCGATGATGGGCGCCCCACTCAACTGCCCAATGAAGTCGATGCCGCTT
TTCCCCTCGAGCGCCCAGACTGGGATTACACCACCCAGGCAGGTAGGAACCACCTAGTCCACTATCGCCAG
TTGCTCCTAGCGGGTCTCCAAAACGCGGGCAGAAGCCCCACCAATTTGGCCAAGGTAAAAGGAATAACACA
AGGGCCCAATGAGTCTCCCTCGGCCTTCCTAGAGAGACTTAAGGAAGCCTATCGCAGGTACACTCCTTATGA
CCCTGAGGACCCAGGGCAAGAAACTAATGTGTCTATGTCTTTCATTTGGCAGTCTGCCCCAGACATTGGGAG
AAAGTTAGAGAGGTTAGAAGATTTAAAAAACAAGACGCTTGGAGATTTGGTTAGAGAGGCAGAAAAGATCTTT
AATAAACGAGAAACCCCGGAAGAAAGAGAGGAACGTATCAGGAGAGAAACAGAGGAAAAAGAAGAACGCC
GTAGGACAGAGGATGAGCAGAAAGAGAAAGAAAGAGATCGTAGGAGACATAGAGAGATGAGCAAGCTATTG
GCCACTGTCGTTAGTGGACAGAAACAGGATAGACAGGGAGGAGAACGAAGGAGGTCCCAACTCGATCGCG
ACCAGTGTGCCTACTGCAAAGAAAAGGGGCACTGGGCTAAAGATTGTCCCAAGAAACCACGAGGACCTCG
GGGACCAAGACCCCAGACCTCCCTCCTGACCCTAGATGACGGCGGATCCACGTCCACGCTGCTAATGGAG
AACTCGTCTGGAGGTTCTAAACGGACAGCCGACGGAAGCGAGTTCGAGTCACCAAAGAAGAAGCGGAAAG
TCGACAAGAAGTACAGCATCGGCCTGGACATCGGCACCAACTCTGTGGGCTGGGCCGTGATCACCGACGA
GTACAAGGTGCCCAGCAAGAAATTCAAGGTGCTGGGCAACACCGACCGGCACAGCATCAAGAAGAACCTG
ATCGGAGCCCTGCTGTTCGACAGCGGCGAAACAGCCGAGGCCACCCGGCTGAAGAGAACCGCCAGAAGA
AGATACACCAGACGGAAGAACCGGATCTGCTATCTGCAAGAGATCTTCAGCAACGAGATGGCCAAGGTGGA
CGACAGCTTCTTCCACAGACTGGAAGAGTCCTTCCTGGTGGAAGAGGATAAGAAGCACGAGCGGCACCCC
ATCTTCGGCAACATCGTGGACGAGGTGGCCTACCACGAGAAGTACCCCACCATCTACCACCTGAGAAAGAA
ACTGGTGGACAGCACCGACAAGGCCGACCTGCGGCTGATCTATCTGGCCCTGGCCCACATGATCAAGTTC

CGGGGCCACTTCCTGATCGAGGGCGACCTGAACCCCGACAACAGCGACGTGGACAAGCTGTTCATCCAGC
TGGTGCAGACCTACAACCAGCTGTTCGAGGAAAACCCCATCAACGCCAGCGGCGTGGACGCCAAGGCCAT
CCTGTCTGCCAGACTGAGCAAGAGCAGAAAGCTGGAAAATCTGATCGCCCAGCTGCCCGGCGAGAAGAAG
AATGGCCTGTTCGGAAACCTGATTGCCCTGAGCCTGGGCCTGACCCCCAACTTCAAGAGCAACTTCGACCT
GGCCGAGGATGCCAAACTGCAGCTGAGCAAGGACACCTACGACGACGACCTGGACAACCTGCTGGCCCA
GATCGGCGACCAGTACGCCGACCTGTTTCTGGCCGCCAAGAACCTGTCCGACGCCATCCTGCTGAGCGAC
ATCCTGAGAGTGAACACCGAGATCACCAAGGCCCCCCTGAGCGCCTCTATGATCAAGAGATACGACGAGCA
CCACCAGGACCTGACCCTGCTGAAAGCTCTCGTGCGGCAGCAGCTGCCTGAGAAGTACAAAGAGATTTTCT
TCGACCAGAGCAAGAACGGCTACGCCGGCTACATTGACGGCGGAGCCAGCCAGGAAGAGTTCTACAAGTT
CATCAAGCCCATCCTGGAAAAGATGGACGGCACCGAGGAACTGCTCGTGAAGCTGAAGAGAGAGGACCTG
CTGCGGAAGCAGCGGACCTTCGACAACGGCAGCATCCCCCACCAGATCCACCTGGGAGAGCTGCACGCC
ATTCTGCGGCGGCAGGAAGATTTTTACCCATTCCTGAAGGACAACCGGGAAAAGATCGAGAAGATCCTGAC
CTTCCGCATCCCCTACTACGTGGGCCCTCTGGCCAGGGGAAACAGCAGATTCGCCTGGATGACCAGAAAG
AGCGAGGAAACCATCACCCCCTGGAACTTCGAGGAAGTGGTGGACAAGGGCGCTTCCGCCCAGAGCTTCA
TCGAGCGGATGACCAACTTCGATAAGAACCTGCCCAACGAGAAGGTGCTGCCCAAGCACAGCCTGCTGTAC
GAGTACTTCACCGTGTATAACGAGCTGACCAAAGTGAAATACGTGACCGAGGGAATGAGAAAGCCCGCCTT
CCTGAGCGGCGAGCAGAAAAAGGCCATCGTGGACCTGCTGTTCAAGACCAACCGGAAAGTGACCGTGAAG
CAGCTGAAAGAGGACTACTTCAAGAAAATCGAGTGCTTCGACTCCGTGGAAATCTCCGGCGTGGAAGATCG
GTTCAACGCCTCCCTGGGCACATACCACGATCTGCTGAAAATTATCAAGGACAAGGACTTCCTGGACAATGA
GGAAAACGAGGACATTCTGGAAGATATCGTGCTGACCCTGACACTGTTTGAGGACAGAGAGATGATCGAGG
AACGGCTGAAAACCTATGCCCACCTGTTCGACGACAAAGTGATGAAGCAGCTGAAGCGGCGGAGATACACC
GGCTGGGGCAGGCTGAGCCGGAAGCTGATCAACGGCATCCGGGACAAGCAGTCCGGCAAGACAATCCTG
GATTTCCTGAAGTCCGACGGCTTCGCCAACAGAAACTTCATGCAGCTGATCCACGACGACAGCCTGACCTT
TAAAGAGGACATCCAGAAAGCCCAGGTGTCCGGCCAGGGCGATAGCCTGCACGAGCACATTGCCAATCTG
GCCGGCAGCCCCGCCATTAAGAAGGGCATCCTGCAGACAGTGAAGGTGGTGGACGAGCTCGTGAAAGTGA
TGGGCCGGCACAAGCCCGAGAACATCGTGATCGAAATGGCCAGAGAGAACCAGACCACCCAGAAGGGACA
GAAGAACAGCCGCGAGAGAATGAAGCGGATCGAAGAGGGCATCAAAGAGCTGGGCAGCCAGATCCTGAAA
GAACACCCCGTGGAAAACACCCAGCTGCAGAACGAGAAGCTGTACCTGTACTACCTGCAGAATGGGCGGG
ATATGTACGTGGACCAGGAACTGGACATCAACCGGCTGTCCGACTACGATGTGGACGCTATCGTGCCTCAG
AGCTTTCTGAAGGACGACTCCATCGACAACAAGGTGCTGACCAGAAGCGACAAGAACCGGGGCAAGAGCG
ACAACGTGCCCTCCGAAGAGGTCGTGAAGAAGATGAAGAACTACTGGCGGCAGCTGCTGAACGCCAAGCT
GATTACCCAGAGAAAGTTCGACAATCTGACCAAGGCCGAGAGAGGCGGCCTGAGCGAACTGGATAAGGCC
GGCTTCATCAAGAGACAGCTGGTGGAAACCCGGCAGATCACAAAGCACGTGGCACAGATCCTGGACTCCC
GGATGAACACTAAGTACGACGAGAATGACAAGCTGATCCGGGAAGTGAAAGTGATCACCCTGAAGTCCAAG
CTGGTGTCCGATTTCCGGAAGGATTTCCAGTTTTACAAAGTGCGCGAGATCAACAACTACCACCACGCCCAC
GACGCCTACCTGAACGCCGTCGTGGGAACCGCCCTGATCAAAAAGTACCCTAAGCTGGAAAGCGAGTTCGT
GTACGGCGACTACAAGGTGTACGACGTGCGGAAGATGATCGCCAAGAGCGAGCAGGAAATCGGCAAGGCT
ACCGCCAAGTACTTCTTCTACAGCAACATCATGAACTTTTTCAAGACCGAGATTACCCTGGCCAACGGCGAG
ATCCGGAAGCGGCCTCTGATCGAGACAAACGGCGAAACCGGGGAGATCGTGTGGGATAAGGGCCGGGATT

TTGCCACCGTGCGGAAAGTGCTGAGCATGCCCCAAGTGAATATCGTGAAAAAGACCGAGGTGCAGACAGG
CGGCTTCAGCAAAGAGTCTATCCTGCCCAAGAGGAACAGCGATAAGCTGATCGCCAGAAAGAAGGACTGG
GACCCTAAGAAGTACGGCGGCTTCGACAGCCCCACCGTGGCCTATTCTGTGCTGGTGGTGGCCAAAGTGG
AAAAGGGCAAGTCCAAGAAACTGAAGAGTGTGAAAGAGCTGCTGGGGATCACCATCATGGAAAGAAGCAG
CTTCGAGAAGAATCCCATCGACTTTCTGGAAGCCAAGGGCTACAAAGAAGTGAAAAAGGACCTGATCATCAA
GCTGCCTAAGTACTCCCTGTTCGAGCTGGAAAACGGCCGGAAGAGAATGCTGGCCTCTGCCGGCGAACTG
CAGAAGGGAAACGAACTGGCCCTGCCCTCCAAATATGTGAACTTCCTGTACCTGGCCAGCCACTATGAGAA
GCTGAAGGGCTCCCCCGAGGATAATGAGCAGAAACAGCTGTTTGTGGAACAGCACAAGCACTACCTGGAC
GAGATCATCGAGCAGATCAGCGAGTTCTCCAAGAGAGTGATCCTGGCCGACGCTAATCTGGACAAAGTGCT
GTCCGCCTACAACAAGCACCGGGATAAGCCCATCAGAGAGCAGGCCGAGAATATCATCCACCTGTTTACCCT
GACCAATCTGGGAGCCCCTGCCGCCTTCAAGTACTTTGACACCACCATCGACCGGAAGAGGTACACCAGCA
CCAAAGAGGTGCTGGACGCCACCCTGATCCACCAGAGCATCACCGGCCTGTACGAGACACGGATCGACCT
GTCTCAGCTGGGAGGTGACTCCGGCGGAAGCTCTGGTGGCAGCAAGCGGACCGCCGACGGCTCTGAATT
CGAGAGCCCTAAGAAGAAAAGAAAGGTGAGCGGAGGCTCTAGCGGCGGAAGCACCCTGAACATTGAAGAC
GAGTATAGACTGCATGAAACAAGCAAGGAACCCGACGTGTCCCTGGGCTCCACCTGGCTGTCCGACTTTCC
CCAGGCCTGGGCCGAGACAGGAGGAATGGGCCTGGCCGTGCGGCAGGCACCCCTGATCATCCCTCTGAA
GGCCACCTCTACACCCGTGAGCATCAAGCAGTACCCTATGTCTCAGGAGGCCAGACTGGGCATCAAGCCTC
ACATCCAGAGGCTGCTGGACCAGGGCATCCTGGTGCCATGCCAGAGCCCCTGGAACACACCACTGCTGCC
CGTGAAGAAGCCAGGCACCAATGACTATAGACCCGTGCAGGATCTGAGAGAGGTGAACAAGAGGGTGGAG
GATATCCACCCCACCGTGCCCAACCCTTACAATCTGCTGTCCGGCCTGCCCCCTTCTCACCAGTGGTATACA
GTGCTGGACCTGAAGGATGCCTTCTTTTGTCTGAGACTGCACCCTACCAGCCAGCCACTGTTCGCCTTTGA
GTGGAGGGACCCTGAGATGGGCATCTCTGGCCAGCTGACCTGGACACGCCTGCCTCAGGGCTTCAAGAAT
AGCCCAACACTGTTTAACGAGGCCCTGCACCGCGACCTGGCAGATTTCCGGATCCAGCACCCAGATCTGAT
CCTGCTGCAGTACGTGGACGATCTGCTGCTGGCCGCCACCAGCGAGCTGGATTGCCAGCAGGGAACACGC
GCCCTGCTGCAGACCCTGGGAAACCTGGGATATAGGGCATCCGCCAAGAAGGCCCAGATCTGTCAGAAGC
AGGTGAAGTACCTGGGCTATCTGCTGAAGGAGGGCCAGAGATGGCTGACAGAGGCCAGGAAGGAGACAGT
GATGGGCCAGCCAACACCCAAGACCCCAAGACAGCTGAGGGAGTTCCTGGGCAAAGCAGGATTTTGCAGG
CTGTTCATCCCAGGATTCGCAGAGATGGCAGCACCTCTGTACCCACTGACCAAGCCGGGCACCCTGTTTAA
TTGGGGCCCTGACCAGCAGAAGGCCTATCAGGAGATCAAGCAGGCCCTGCTGACAGCACCAGCCCTGGGC
CTGCCAGACCTGACCAAGCCTTTCGAGCTGTTTGTGGATGAGAAGCAGGGCTACGCCAAGGGCGTGCTGA
CCCAGAAGCTGGGACCATGGAGACGGCCCGTGGCCTATCTGTCCAAGAAGCTGGACCCAGTGGCAGCAG
GATGGCCACCATGCCTGAGGATGGTGGCAGCAATCGCCGTGCTGACAAAGGATGCCGGCAAGCTGACCAT
GGGACAGCCACTGGTCATCCTGGCACCACACGCAGTGGAGGCCCTGGTGAAGCAGCCTCCAGATCGCTG
GCTGTCTAACGCCCGGATGACACACTACCAGGCCCTGCTGCTGGACACCGATCGCGTGCAGTTTGGCCCT
GTGGTGGCCCTGAATCCAGCCACCCTGCTGCCTCTGCCAGAGGAGGGCCTGCAGCACAACTGTCTGGACA
TCCTGGCAGAGGCACACGGAACAAGGCCAGACCTGACCGATCAGCCCCTGCCTGACGCCGATCACACATG
GTATACCGATGGAAGCTCCCTGCTGCAGGAGGGCCAGAGGAAGGCAGGAGCAGCAGTGACCACAGAGAC
AGAAGTGATCTGGGCCAAGGCCCTGCCAGCAGGCACATCCGCCCAGCGGGCCGAGCTGATCGCCCTGAC
CCAGGCCCTGAAGATGGCCGAGGGCAAGAAGCTGAACGTGTACACAGACTCCAGATATGCCTTCGCCACC

GCACACATCCACGGAGAGATCTACAGGCGCCGGGGCTGGCTGACCTCTGAGGGCAAGGAGATCAAGAACA
AGGATGAGATCCTGGCCCTGCTGAAGGCCCTGTTTCTGCCCAAGCGGCTGAGCATCATCCACTGTCCTGGA
CACCAGAAGGGACACTCCGCCGAGGCAAGGGGCAATCGGATGGCCGACCAGGCCGCCAGAAAGGCTGCT
ATTACTGAAACTCCCGACACTTCCACTCTGCTGTCTGGCGGCTCAAAAAGAACCGCCGACGGCAGCGAATT
CGAGTCTCCCAAGAAGAAGAGGAAAGTCGGCTCTGGCCCTGCCGCTAAGAGAGTGAAGCTGGACTGA

Supplementary Sequence 3. Gag-MCP-pol in v3 PE-eVLPs.

Key: Gag-linker-**MCP**-linker-pol

ATGGGCCAGACTGTTACCACTCCCTTAAGTTTGACCTTAGGTCACTGGAAAGATGTCGAGCGGATCGCTCAC
AACCAGTCGGTAGATGTCAAGAAGAGACGTTGGGTTACCTTCTGCTCTGCAGAATGGCCAACCTTTAACGTC
GGATGGCCGCGAGACGGCACCTTTAACCGAGACCTCATCACCCAGGTTAAGATCAAGGTCTTTTCACCTGG
CCCGCATGGACACCCAGACCAGGTCCCCTACATCGTGACCTGGGAAGCCTTGGCTTTTGACCCCCCTCCCT
GGGTCAAGCCCTTTGTACACCCTAAGCCTCCGCCTCCTCTTCCTCCATCCGCCCCGTCTCTCCCCCTTGAA
CCTCCTCGTTCGACCCCGCCTCGATCCTCCCTTTATCCAGCCCTCACTCCTTCTCTAGGCGCCAAACCTAAA
CCTCAAGTTCTTTCTGACAGTGGGGGGCCGCTCATCGACCTACTTACAGAAGACCCCCCGCCTTATAGGGA
CCCAAGACCACCCCCTTCCGACAGGGACGGAAATGGTGGAGAAGCGACCCCTGCGGGAGAGGCACCGGA
CCCCTCCCCAATGGCATCTCGCCTACGTGGGAGACGGGAGCCCCCTGTGGCCGACTCCACTACCTCGCAG
GCATTCCCCCTCCGCGCAGGAGGAAACGGACAGCTTCAATACTGGCCGTTCTCCTCTTCTGACCTTTACAA
CTGGAAAAATAATAACCCTTCTTTTTCTGAAGATCCAGGTAAACTGACAGCTCTGATCGAGTCTGTCCTCATC
ACCCATCAGCCCACCTGGGACGACTGTCAGCAGCTGTTGGGGACTCTGCTGACCGGAGAAGAAAAACAAC
GGGTGCTCTTAGAGGCTAGAAAGGCGGTGCGGGGCGATGATGGGCGCCCCACTCAACTGCCCAATGAAGT
CGATGCCGCTTTTCCCCTCGAGCGCCCAGACTGGGATTACACCACCCAGGCAGGTAGGAACCACCTAGTC
CACTATCGCCAGTTGCTCCTAGCGGGTCTCCAAAACGCGGGCAGAAGCCCCACCAATTTGGCCAAGGTAAA
AGGAATAACACAAGGGCCCAATGAGTCTCCCTCGGCCTTCCTAGAGAGACTTAAGGAAGCCTATCGCAGGT
ACACTCCTTATGACCCTGAGGACCCAGGGCAAGAAACTAATGTGTCTATGTCTTTCATTTGGCAGTCTGCCC
CAGACATTGGGAGAAAGTTAGAGAGGTTAGAAGATTTAAAAAACAAGACGCTTGGAGATTTGGTTAGAGAGG
CAGAAAAGATCTTTAATAAACGAGAAACCCCGGAAGAAAGAGAGGAACGTATCAGGAGAGAAACAGAGGAA
AAAGAAGAACGCCGTAGGACAGAGGATGAGCAGAAAGAGAAAGAAAGAGATCGTAGGAGACATAGAGAGAT
GAGCAAGCTATTGGCCACTGTCGTTAGTGGACAGAAACAGGATAGACAGGGAGGAGAACGAAGGAGGTCC
CAACTCGATCGCGACCAGTGTGCCTACTGCAAAGAAAAGGGGCACTGGGCTAAAGATTGTCCCAAGAAACC
ACGAGGACCTCGGGGACCAAGACCCCAGACCTCCCTCCTGACCCTAGATGACTCCGGTGGAGGTGGATCC
GGTGGAGGTTCC**ATGGCCTCTAATTTTACTCAATTTGTGCTTGTCGATAATGGGGGGACGGGAGATGTGAC**
CGTTGCCCCTAGCAATTTCGCAAATGGCGTTGCAGAATGGATCTCTAGCAACAGCAGAAGCCAAGCGTAC
AAAGTAACGTGTTCCGTTCGCCAAAGCTCCGCCCAAAAACGGAAGTATACAATAAAGGTTGAGGTGCCGA
AAGTAGCCACTCAAACAGTTGGTGGGGTAGAATTGCCCGTAGCGGCATGGCGGTCATATCTCAATATGGA
ACTCACTATCCCAATCTTCGCCACGAATAGCGATTGTGAGCTGATAGTTAAGGCTATGCAAGGTCTTCTCA
AAGATGGAAACCCTATTCCATCTGCTATCGCCGCCAACAGCGGGATATACTCCGGAGGTGGAGGTGGATA
GGGAGGTCAGGGTCAGGAGCCCCCCCTGAACCCAGGATAACCCTCAAAGTCGGGGGGCAACCCGTCAC
CTTCCTGGTAGATACTGGGGCCCAACACTCCGTGCTGACCCAAAATCCTGGACCCCTAAGTGATAAGTCTGC
CTGGGTCCAAGGGGCTACTGGAGGAAAGCGGTATCGCTGGACCACGGATCGCAAAGTACATCTAGCTACCG
GTAAGGTCACCCACTCTTTCCTCCATGTACCAGACTGTCCCTATCCTCTGTTAGGAAGAGATTTGCTGACTAA
ACTAAAAGCCCAAATCCACTTTGAGGGATCAGGAGCTCAGGTTATGGGACCAATGGGGCAGCCCCTGCAAG
TGTTGACCCTAAATATAGAAGATGAGTATCGGCTACATGAGACCTCAAAAGAGCCAGATGTTTCTCTAGGGTC

CACATGGCTGTCTGATTTTCCTCAGGCCTGGGCGGAAACCGGGGGCATGGGACTGGCAGTTCGCCAAGCT
CCTCTGATCATACCTCTGAAAGCAACCTCTACCCCCGTGTCCATAAAACAATACCCCATGTCACAAGAAGCCA
GACTGGGGATCAAGCCCCACATACAGAGACTGTTGGACCAGGGAATACTGGTACCCTGCCAGTCCCCCTGG
AACACGCCCCTGCTACCCGTTAAGAAACCAGGGACTAATGATTATAGGCCTGTCCAGGATCTGAGAGAAGTC
AACAAGCGGGTGGAAGACATCCACCCCACCGTGCCCAACCCTTACAACCTCTTGAGCGGGCTCCCACCGT
CCCACCAGTGGTACACTGTGCTTGATTTAAAGGATGCCTTTTTCTGCCTGAGACTCCACCCCACCAGTCAGC
CTCTCTTCGCCTTTGAGTGGAGAGATCCAGAGATGGGAATCTCAGGACAATTGACCTGGACCAGACTCCCA
CAGGGTTTCAAAAACAGTCCCACCCTGTTTGATGAGGCACTGCACAGAGACCTAGCAGACTTCCGGATCCA
GCACCCAGACTTGATCCTGCTACAGTACGTGGATGACTTACTGCTGGCCGCCACTTCTGAGCTAGACTGCC
AACAAGGTACTCGGGCCCTGTTACAAACCCTAGGGAACCTCGGGTATCGGGCCTCGGCCAAGAAAGCCCA
AATTTGCCAGAAACAGGTCAAGTATCTGGGGTATCTTCTAAAAGAGGGTCAGAGATGGCTGACTGAGGCCA
GAAAAGAGACTGTGATGGGGCAGCCTACTCCGAAGACCCCTCGACAACTAAGGGAGTTCCTAGGGACGGC
AGGCTTCTGTCGCCTCTGGATCCCTGGGTTTGCAGAAATGGCAGCCCCCTTGTACCCTCTCACCAAAACGG
GGACTCTGTTTAATTGGGGCCCAGACCAACAAAAGGCCTATCAAGAAATCAAGCAAGCTCTTCTAACTGCCC
CAGCCCTGGGGTTGCCAGATTTGACTAAGCCCTTTGAACTCTTTGTCGACGAGAAGCAGGGCTACGCCAAA
GGTGTCCTAACGCAAAAACTGGGACCTTGGCGTCGGCCGGTGGCCTACCTGTCCAAAAAGCTAGACCCAG
TAGCAGCTGGGTGGCCCCCTTGCCTACGGATGGTAGCAGCCATTGCCGTACTGACAAAGGATGCAGGCAA
GCTAACCATGGGACAGCCACTAGTCATTCTGGCCCCCCATGCAGTAGAGGCACTAGTCAAACAACCCCCCG
ACCGCTGGCTTTCCAACGCCCGGATGACTCACTATCAGGCCTTGCTTTTGGACACGGACCGGGTCCAGTTC
GGACCGGTGGTAGCCCTGAACCCGGCTACGCTGCTCCCACTGCCTGAGGAAGGGCTGCAACACAACTGC
CTTGATATCCTGGCCGAAGCCCACGGAACCCGACCCGACCTAACGGACCAGCCGCTCCCAGACGCCGACC
ACACCTGGTACACGGATGGAAGCAGTCTCTTACAAGAGGGACAGCGTAAGGCGGGAGCTGCGGTGACCAC
CGAGACCGAGGTAATCTGGGCTAAAGCCCTGCCAGCCGGGACATCCGCTCAGCGGGCTGAACTGATAGCA
CTCACCCAGGCCCTAAAGATGGCAGAAGGTAAGAAGCTAAATGTTTATACTGATAGCCGTTATGCTTTTGCTA
CTGCCCATATCCATGGAGAAATATACAGAAGGCGTGGGTTGCTCACATCAGAAGGCAAAGAGATCAAAAATA
AAGACGAGATCTTGGCCCTACTAAAAGCCCTCTTTCTGCCCAAAAGACTTAGCATAATCCATTGTCCAGGACA
TCAAAAGGGACACAGCGCCGAGGCTAGAGGCAACCGGATGGCTGACCAAGCGGCCCGAAAGGCAGCCAT
CACAGAGACTCCAGACACCTCTACCCTCCTCATAGAAAATTCATCACCCTACACCTCAGAACATTTTCATTAC
ACAGTGACTGATATAAAGGACCTAACCAAGTTGGGGGCCATTTATGATAAAACAAAGAAGTATTGGGTCTACC
AAGGAAAACCTGTGATGCCTGACCAGTTTACTTTTGAATTATTAGACTTTCTTCATCAGCTGACTCACCTCAG
CTTCTCAAAAATGAAGGCTCTCCTAGAGAGAAGCCACAGTCCCTACTACATGCTGAACCGGGATCGAACACT
CAAAAATATCACTGAGACCTGCAAAGCTTGTGCACAAGTCAACGCCAGCAAGTCTGCCGTTAAACAGGGAA
CTAGGGTCCGCGGGCATCGGCCCGGCACTCATTGGGAGATCGATTTCACCGAGATAAAGCCCGGATTGTAT
GGCTATAAATATCTTCTAGTTTTTATAGATACCTTTTCTGGCTGGATAGAAGCCTTCCCAACCAAGAAAGAAAC
CGCCAAGGTCGTAACCAAGAAGCTACTAGAGGAGATCTTCCCCAGGTTCGGCATGCCTCAGGTATTGGGAA
CTGACAATGGGCCTGCCTTCGTCTCCAAGGTGAGTCAGACAGTGGCCGATCTGTTGGGGATTGATTGGAAA
TTACATTGTGCATACAGACCCCAAAGCTCAGGCCAGGTAGAAAGAATGAATAGAACCATCAAGGAGACTTTAA
CTAAATTAACGCTTGCAACTGGCTCTAGAGACTGGGTGCTCCTACTCCCCTTAGCCCTGTACCGAGCCCGCA
ACACGCCGGGCCCCCATGGCCTCACCCCATATGAGATCTTATATGGGGCACCCCCGCCCCTTGTAAACTTC

CCTGACCCTGACATGACAAGAGTTACTAACAGCCCCTCTCTCCAAGCTCACTTACAGGCTCTCTACTTAGTC
CAGCACGAAGTCTGGAGACCTCTGGCGGCAGCCTACCAAGAACAACTGGACCGACCGGTGGTACCTCACC
CTTACCGAGTCGGCGACACAGTGTGGGTCCGCCGACACCAGACTAAGAACCTAGAACCTCGCTGGAAAGG
ACCTTACACAGTCCTGCTGACCACCCCCACCGCCCTCAAAGTAGACGGCATCGCAGCTTGGATACACGCCG
CCCACGTGAAGGCTGCCGACCCCGGGGGTGGACCATCCTCTAGACTGACATGGCGCGTTCAACGCTCTCA
AAACCCCTTAAAAATAAGGTTGACGCGTCAACCCGCGAGGCCCCCTAATCCCCTTAATTCTTCTGATGCTCA
GAGGGGTCAGTATCGAATTCCTGCAGCCCCGCTGATCAGCCTCGACTGTGCCTTCTAG

Supplementary Sequence 4. P4-PE in v3b PE-eVLPs.

Key: P4-linker-3xNES- engineered protease cleavage site-linker- PEmax with 6 amino acid deletion at C-terminus of MMLV RT (NLS-Cas9 nickase (R221K, N393K, H840A)-linker-MMLV RT (6 amino acid del)-NLS-cmycNLS)

ATGTCTCCCGAAGATAAAATAGCTCAACTCAAACAGAAAATTCAGGCTTTGAAACAAGAAAACCAGCAGTTG
GAGGAAGAAAATGCGGCCCTGGAGTATGGCAGTGGCGGTAGTTCTGGCGGCTCACTTCAACTGCCTCCAC
TTGAAAGACTGACACTGGGATCATTACAATTACCTCCTTTAGAACGATTAACACTCGGTTCACTACAGCTTCC
GCCTCTTGAGAGATTGACATTAAGTGGTGGATCTACGTCCACGCTGCTAATGGAGAACTCGTCTGGAGGTTC
TGATTACAAAGACGATGACGATAAGAAACGGACAGCCGACGGAAGCGAGTTCGAGTCACCAAAGAAGAAGC
GGAAAGTCGACAAGAAGTACAGCATCGGCCTGGACATCGGCACCAACTCTGTGGGCTGGGCCGTGATCAC
CGACGAGTACAAGGTGCCCAGCAAGAAATTCAAGGTGCTGGGCAACACCGACCGGCACAGCATCAAGAAG
AACCTGATCGGAGCCCTGCTGTTCGACAGCGGCGAAACAGCCGAGGCCACCCGGCTGAAGAGAACCGCC
AGAAGAAGATACACCAGACGGAAGAACCGGATCTGCTATCTGCAAGAGATCTTCAGCAACGAGATGGCCAA
GGTGGACGACAGCTTCTTCCACAGACTGGAAGAGTCCTTCCTGGTGGAAGAGGATAAGAAGCACGAGCGG
CACCCCATCTTCGGCAACATCGTGGACGAGGTGGCCTACCACGAGAAGTACCCCACCATCTACCACCTGAG
AAAGAAACTGGTGGACAGCACCGACAAGGCCGACCTGCGGCTGATCTATCTGGCCCTGGCCCACATGATC
AAGTTCCGGGGCCACTTCCTGATCGAGGGCGACCTGAACCCCGACAACAGCGACGTGGACAAGCTGTTCA
TCCAGCTGGTGCAGACCTACAACCAGCTGTTCGAGGAAAACCCCATCAACGCCAGCGGCGTGGACGCCAA
GGCCATCCTGTCTGCCAGACTGAGCAAGAGCAGAAAGCTGGAAAATCTGATCGCCCAGCTGCCCGGCGAG
AAGAAGAATGGCCTGTTCGGAAACCTGATTGCCCTGAGCCTGGGCCTGACCCCCAACTTCAAGAGCAACTT
CGACCTGGCCGAGGATGCCAAACTGCAGCTGAGCAAGGACACCTACGACGACGACCTGGACAACCTGCTG
GCCCAGATCGGCGACCAGTACGCCGACCTGTTTCTGGCCGCCAAGAACCTGTCCGACGCCATCCTGCTGA
GCGACATCCTGAGAGTGAACACCGAGATCACCAAGGCCCCCCTGAGCGCCTCTATGATCAAGAGATACGAC
GAGCACCACCAGGACCTGACCCTGCTGAAAGCTCTCGTGCGGCAGCAGCTGCCTGAGAAGTACAAAGAGA
TTTTCTTCGACCAGAGCAAGAACGGCTACGCCGGCTACATTGACGGCGGAGCCAGCCAGGAAGAGTTCTAC
AAGTTCATCAAGCCCATCCTGGAAAAGATGGACGGCACCGAGGAACTGCTCGTGAAGCTGAAGAGAGAGG
ACCTGCTGCGGAAGCAGCGGACCTTCGACAACGGCAGCATCCCCCACCAGATCCACCTGGGAGAGCTGCA
CGCCATTCTGCGGCGGCAGGAAGATTTTTACCCATTCCTGAAGGACAACCGGGAAAAGATCGAGAAGATCC
TGACCTTCCGCATCCCCTACTACGTGGGCCCTCTGGCCAGGGGAAACAGCAGATTCGCCTGGATGACCAG
AAAGAGCGAGGAAACCATCACCCCCTGGAACTTCGAGGAAGTGGTGGACAAGGGCGCTTCCGCCCAGAG
CTTCATCGAGCGGATGACCAACTTCGATAAGAACCTGCCCAACGAGAAGGTGCTGCCCAAGCACAGCCTGC
TGTACGAGTACTTCACCGTGTATAACGAGCTGACCAAAGTGAAATACGTGACCGAGGGAATGAGAAAGCCC
GCCTTCCTGAGCGGCGAGCAGAAAAAGGCCATCGTGGACCTGCTGTTCAAGACCAACCGGAAAGTGACCG
TGAAGCAGCTGAAAGAGGACTACTTCAAGAAAATCGAGTGCTTCGACTCCGTGGAAATCTCCGGCGTGGAA
GATCGGTTCAACGCCTCCCTGGGCACATACCACGATCTGCTGAAAATTATCAAGGACAAGGACTTCCTGGAC
AATGAGGAAAACGAGGACATTCTGGAAGATATCGTGCTGACCCTGACACTGTTTGAGGACAGAGAGATGATC
GAGGAACGGCTGAAAACCTATGCCCACCTGTTCGACGACAAAGTGATGAAGCAGCTGAAGCGGCGGAGAT

ACACCGGCTGGGGCAGGCTGAGCCGGAAGCTGATCAACGGCATCCGGGACAAGCAGTCCGGCAAGACAA
TCCTGGATTTCCTGAAGTCCGACGGCTTCGCCAACAGAAACTTCATGCAGCTGATCCACGACGACAGCCTG
ACCTTTAAAGAGGACATCCAGAAAGCCCAGGTGTCCGGCCAGGGCGATAGCCTGCACGAGCACATTGCCA
ATCTGGCCGGCAGCCCCGCCATTAAGAAGGGCATCCTGCAGACAGTGAAGGTGGTGGACGAGCTCGTGAA
AGTGATGGGCCGGCACAAGCCCGAGAACATCGTGATCGAAATGGCCAGAGAGAACCAGACCACCCAGAAG
GGACAGAAGAACAGCCGCGAGAGAATGAAGCGGATCGAAGAGGGCATCAAAGAGCTGGGCAGCCAGATC
CTGAAAGAACACCCCGTGGAAAACACCCAGCTGCAGAACGAGAAGCTGTACCTGTACTACCTGCAGAATGG
GCGGGATATGTACGTGGACCAGGAACTGGACATCAACCGGCTGTCCGACTACGATGTGGACGCTATCGTGC
CTCAGAGCTTTCTGAAGGACGACTCCATCGACAACAAGGTGCTGACCAGAAGCGACAAGAACCGGGGCAA
GAGCGACAACGTGCCCTCCGAAGAGGTCGTGAAGAAGATGAAGAACTACTGGCGGCAGCTGCTGAACGCC
AAGCTGATTACCCAGAGAAAGTTCGACAATCTGACCAAGGCCGAGAGAGGCGGCCTGAGCGAACTGGATAA
GGCCGGCTTCATCAAGAGACAGCTGGTGGAAACCCGGCAGATCACAAAGCACGTGGCACAGATCCTGGAC
TCCCGGATGAACACTAAGTACGACGAGAATGACAAGCTGATCCGGGAAGTGAAAGTGATCACCCTGAAGTC
CAAGCTGGTGTCCGATTTCCGGAAGGATTTCCAGTTTTACAAAGTGCGCGAGATCAACAACTACCACCACGC
CCACGACGCCTACCTGAACGCCGTCGTGGGAACCGCCCTGATCAAAAAGTACCCTAAGCTGGAAAGCGAG
TTCGTGTACGGCGACTACAAGGTGTACGACGTGCGGAAGATGATCGCCAAGAGCGAGCAGGAAATCGGCA
AGGCTACCGCCAAGTACTTCTTCTACAGCAACATCATGAACTTTTTCAAGACCGAGATTACCCTGGCCAACG
GCGAGATCCGGAAGCGGCCTCTGATCGAGACAAACGGCGAAACCGGGGAGATCGTGTGGGATAAGGGCC
GGGATTTTGCCACCGTGCGGAAAGTGCTGAGCATGCCCCAAGTGAATATCGTGAAAAAGACCGAGGTGCAG
ACAGGCGGCTTCAGCAAAGAGTCTATCCTGCCCAAGAGGAACAGCGATAAGCTGATCGCCAGAAAGAAGGA
CTGGGACCCTAAGAAGTACGGCGGCTTCGACAGCCCCACCGTGGCCTATTCTGTGCTGGTGGTGGCCAAA
GTGGAAAAGGGCAAGTCCAAGAAACTGAAGAGTGTGAAAGAGCTGCTGGGGATCACCATCATGGAAAGAA
GCAGCTTCGAGAAGAATCCCATCGACTTTCTGGAAGCCAAGGGCTACAAAGAAGTGAAAAAGGACCTGATC
ATCAAGCTGCCTAAGTACTCCCTGTTCGAGCTGGAAAACGGCCGGAAGAGAATGCTGGCCTCTGCCGGCG
AACTGCAGAAGGGAAACGAACTGGCCCTGCCCTCCAAATATGTGAACTTCCTGTACCTGGCCAGCCACTAT
GAGAAGCTGAAGGGCTCCCCCGAGGATAATGAGCAGAAACAGCTGTTTGTGGAACAGCACAAGCACTACCT
GGACGAGATCATCGAGCAGATCAGCGAGTTCTCCAAGAGAGTGATCCTGGCCGACGCTAATCTGGACAAAG
TGCTGTCCGCCTACAACAAGCACCGGGATAAGCCCATCAGAGAGCAGGCCGAGAATATCATCCACCTGTTTA
CCCTGACCAATCTGGGAGCCCCTGCCGCCTTCAAGTACTTTGACACCACCATCGACCGGAAGAGGTACACC
AGCACCAAAGAGGTGCTGGACGCCACCCTGATCCACCAGAGCATCACCGGCCTGTACGAGACACGGATCG
ACCTGTCTCAGCTGGGAGGTGACTCCGGCGGAAGCTCTGGTGGCAGCAAGCGGACCGCCGACGGCTCTG
AATTCGAGAGCCCTAAGAAGAAAAGAAAGGTGAGCGGAGGCTCTAGCGGCGGAAGCACCCTGAACATTGA
AGACGAGTATAGACTGCATGAAACAAGCAAGGAACCCGACGTGTCCCTGGGCTCCACCTGGCTGTCCGACT
TTCCCCAGGCCTGGGCCGAGACAGGAGGAATGGGCCTGGCCGTGCGGCAGGCACCCCTGATCATCCCTC
TGAAGGCCACCTCTACACCCGTGAGCATCAAGCAGTACCCTATGTCTCAGGAGGCCAGACTGGGCATCAAG
CCTCACATCCAGAGGCTGCTGGACCAGGGCATCCTGGTGCCATGCCAGAGCCCCTGGAACACACCACTGC
TGCCCGTGAAGAAGCCAGGCACCAATGACTATAGACCCGTGCAGGATCTGAGAGAGGTGAACAAGAGGGT
GGAGGATATCCACCCCACCGTGCCCAACCCTTACAATCTGCTGTCCGGCCTGCCCCCTTCTCACCAGTGGT
ATACAGTGCTGGACCTGAAGGATGCCTTCTTTTGTCTGAGACTGCACCCTACCAGCCAGCCACTGTTCGCC

TTTGAGTGGAGGGACCCTGAGATGGGCATCTCTGGCCAGCTGACCTGGACACGCCTGCCTCAGGGCTTCA
AGAATAGCCCAACACTGTTTAACGAGGCCCTGCACCGCGACCTGGCAGATTTCCGGATCCAGCACCCAGAT
CTGATCCTGCTGCAGTACGTGGACGATCTGCTGCTGGCCGCCACCAGCGAGCTGGATTGCCAGCAGGGAA
CACGCGCCCTGCTGCAGACCCTGGGAAACCTGGGATATAGGGCATCCGCCAAGAAGGCCCAGATCTGTCA
GAAGCAGGTGAAGTACCTGGGCTATCTGCTGAAGGAGGGCCAGAGATGGCTGACAGAGGCCAGGAAGGA
GACAGTGATGGGCCAGCCAACACCCAAGACCCCAAGACAGCTGAGGGAGTTCCTGGGCAAAGCAGGATTT
TGCAGGCTGTTCATCCCAGGATTCGCAGAGATGGCAGCACCTCTGTACCCACTGACCAAGCCGGGCACCC
TGTTTAATTGGGGCCCTGACCAGCAGAAGGCCTATCAGGAGATCAAGCAGGCCCTGCTGACAGCACCAGC
CCTGGGCCTGCCAGACCTGACCAAGCCTTTCGAGCTGTTTGTGGATGAGAAGCAGGGCTACGCCAAGGGC
GTGCTGACCCAGAAGCTGGGACCATGGAGACGGCCCGTGGCCTATCTGTCCAAGAAGCTGGACCCAGTGG
CAGCAGGATGGCCACCATGCCTGAGGATGGTGGCAGCAATCGCCGTGCTGACAAAGGATGCCGGCAAGCT
GACCATGGGACAGCCACTGGTCATCCTGGCACCACACGCAGTGGAGGCCCTGGTGAAGCAGCCTCCAGAT
CGCTGGCTGTCTAACGCCCGGATGACACACTACCAGGCCCTGCTGCTGGACACCGATCGCGTGCAGTTTG
GCCCTGTGGTGGCCCTGAATCCAGCCACCCTGCTGCCTCTGCCAGAGGAGGGCCTGCAGCACAACTGTCT
GGACATCCTGGCAGAGGCACACGGAACAAGGCCAGACCTGACCGATCAGCCCCTGCCTGACGCCGATCAC
ACATGGTATACCGATGGAAGCTCCCTGCTGCAGGAGGGCCAGAGGAAGGCAGGAGCAGCAGTGACCACAG
AGACAGAAGTGATCTGGGCCAAGGCCCTGCCAGCAGGCACATCCGCCCAGCGGGCCGAGCTGATCGCCC
TGACCCAGGCCCTGAAGATGGCCGAGGGCAAGAAGCTGAACGTGTACACAGACTCCAGATATGCCTTCGC
CACCGCACACATCCACGGAGAGATCTACAGGCGCCGGGGCTGGCTGACCTCTGAGGGCAAGGAGATCAAG
AACAAGGATGAGATCCTGGCCCTGCTGAAGGCCCTGTTTCTGCCCAAGCGGCTGAGCATCATCCACTGTCC
TGGACACCAGAAGGGACACTCCGCCGAGGCAAGGGGCAATCGGATGGCCGACCAGGCCGCCAGAAAGGC
TGCTATTACTGAAACTCCCGACACTTCCACTCTGCTGTCTGGCGGCTCAAAAAGAACCGCCGACGGCAGCG
AATTCGAGTCTCCCAAGAAGAAGAGGAAAGTCGGCTCTGGCCCTGCCGCTAAGAGAGTGAAGCTGGACTG
A

Supplementary Sequence 5. Gag-P3-pol in v3b PE-eVLPs.

Key: Gag-linker-**P3**-linker-pol

ATGGGCCAGACTGTTACCACTCCCTTAAGTTTGACCTTAGGTCACTGGAAAGATGTCGAGCGGATCGCTCAC
AACCAGTCGGTAGATGTCAAGAAGAGACGTTGGGTTACCTTCTGCTCTGCAGAATGGCCAACCTTTAACGTC
GGATGGCCGCGAGACGGCACCTTTAACCGAGACCTCATCACCCAGGTTAAGATCAAGGTCTTTTCACCTGG
CCCGCATGGACACCCAGACCAGGTCCCCTACATCGTGACCTGGGAAGCCTTGGCTTTTGACCCCCCTCCCT
GGGTCAAGCCCTTTGTACACCCTAAGCCTCCGCCTCCTCTTCCTCCATCCGCCCCGTCTCTCCCCCTTGAA
CCTCCTCGTTCGACCCCGCCTCGATCCTCCCTTTATCCAGCCCTCACTCCTTCTCTAGGCGCCAAACCTAAA
CCTCAAGTTCTTTCTGACAGTGGGGGGCCGCTCATCGACCTACTTACAGAAGACCCCCCGCCTTATAGGGA
CCCAAGACCACCCCCTTCCGACAGGGACGGAAATGGTGGAGAAGCGACCCCTGCGGGAGAGGCACCGGA
CCCCTCCCCAATGGCATCTCGCCTACGTGGGAGACGGGAGCCCCCTGTGGCCGACTCCACTACCTCGCAG
GCATTCCCCCTCCGCGCAGGAGGAAACGGACAGCTTCAATACTGGCCGTTCTCCTCTTCTGACCTTTACAA
CTGGAAAAATAATAACCCTTCTTTTTCTGAAGATCCAGGTAAACTGACAGCTCTGATCGAGTCTGTCCTCATC
ACCCATCAGCCCACCTGGGACGACTGTCAGCAGCTGTTGGGGACTCTGCTGACCGGAGAAGAAAAACAAC
GGGTGCTCTTAGAGGCTAGAAAGGCGGTGCGGGGCGATGATGGGCGCCCCACTCAACTGCCCAATGAAGT
CGATGCCGCTTTTCCCCTCGAGCGCCCAGACTGGGATTACACCACCCAGGCAGGTAGGAACCACCTAGTC
CACTATCGCCAGTTGCTCCTAGCGGGTCTCCAAAACGCGGGCAGAAGCCCCACCAATTTGGCCAAGGTAAA
AGGAATAACACAAGGGCCCAATGAGTCTCCCTCGGCCTTCCTAGAGAGACTTAAGGAAGCCTATCGCAGGT
ACACTCCTTATGACCCTGAGGACCCAGGGCAAGAAACTAATGTGTCTATGTCTTTCATTTGGCAGTCTGCCC
CAGACATTGGGAGAAAGTTAGAGAGGTTAGAAGATTTAAAAAACAAGACGCTTGGAGATTTGGTTAGAGAGG
CAGAAAAGATCTTTAATAAACGAGAAACCCCGGAAGAAAGAGAGGAACGTATCAGGAGAGAAACAGAGGAA
AAAGAAGAACGCCGTAGGACAGAGGATGAGCAGAAAGAGAAAGAAAGAGATCGTAGGAGACATAGAGAGAT
GAGCAAGCTATTGGCCACTGTCGTTAGTGGACAGAAACAGGATAGACAGGGAGGAGAACGAAGGAGGTCC
CAACTCGATCGCGACCAGTGTGCCTACTGCAAAGAAAAGGGGCACTGGGCTAAAGATTGTCCCAAGAAACC
ACGAGGACCTCGGGGACCAAGACCCCAGACCTCCCTCCTGACCCTAGATGACTCCGGTGGAGGTGGATCC
GGTGGAGGTTCC**TCCCCGGAAGACGAAATCCAACAGTTGGAGGAAGAGATCGCACAACTTGAGCAGAA**
AAACGCAGCACTCAAAGAGAAAAACCAAGCACTGAAGTACGGGTCCGGAGGTGGAGGTGGATAGGGAG
GTCAGGGTCAGGAGCCCCCCCTGAACCCAGGATAACCCTCAAAGTCGGGGGGCAACCCGTCACCTTCCT
GGTAGATACTGGGGCCCAACACTCCGTGCTGACCCAAAATCCTGGACCCCTAAGTGATAAGTCTGCCTGGG
TCCAAGGGGCTACTGGAGGAAAGCGGTATCGCTGGACCACGGATCGCAAAGTACATCTAGCTACCGGTAAG
GTCACCCACTCTTTCCTCCATGTACCAGACTGTCCCTATCCTCTGTTAGGAAGAGATTTGCTGACTAAACTAA
AAGCCCAAATCCACTTTGAGGGATCAGGAGCTCAGGTTATGGGACCAATGGGGCAGCCCCTGCAAGTGTTG
ACCCTAAATATAGAAGATGAGTATCGGCTACATGAGACCTCAAAAGAGCCAGATGTTTCTCTAGGGTCCACAT
GGCTGTCTGATTTTCCTCAGGCCTGGGCGGAAACCGGGGGCATGGGACTGGCAGTTCGCCAAGCTCCTCT
GATCATACCTCTGAAAGCAACCTCTACCCCCGTGTCCATAAAACAATACCCCATGTCACAAGAAGCCAGACT
GGGGATCAAGCCCCACATACAGAGACTGTTGGACCAGGGAATACTGGTACCCTGCCAGTCCCCCTGGAACA
CGCCCCTGCTACCCGTTAAGAAACCAGGGACTAATGATTATAGGCCTGTCCAGGATCTGAGAGAAGTCAACA

AGCGGGTGGAAGACATCCACCCCACCGTGCCCAACCCTTACAACCTCTTGAGCGGGCTCCCACCGTCCCA
CCAGTGGTACACTGTGCTTGATTTAAAGGATGCCTTTTTCTGCCTGAGACTCCACCCCACCAGTCAGCCTCT
CTTCGCCTTTGAGTGGAGAGATCCAGAGATGGGAATCTCAGGACAATTGACCTGGACCAGACTCCCACAGG
GTTTCAAAAACAGTCCCACCCTGTTTGATGAGGCACTGCACAGAGACCTAGCAGACTTCCGGATCCAGCAC
CCAGACTTGATCCTGCTACAGTACGTGGATGACTTACTGCTGGCCGCCACTTCTGAGCTAGACTGCCAACAA
GGTACTCGGGCCCTGTTACAAACCCTAGGGAACCTCGGGTATCGGGCCTCGGCCAAGAAAGCCCAAATTTG
CCAGAAACAGGTCAAGTATCTGGGGTATCTTCTAAAAGAGGGTCAGAGATGGCTGACTGAGGCCAGAAAAG
AGACTGTGATGGGGCAGCCTACTCCGAAGACCCCTCGACAACTAAGGGAGTTCCTAGGGACGGCAGGCTT
CTGTCGCCTCTGGATCCCTGGGTTTGCAGAAATGGCAGCCCCCTTGTACCCTCTCACCAAAACGGGGACTC
TGTTTAATTGGGGCCCAGACCAACAAAAGGCCTATCAAGAAATCAAGCAAGCTCTTCTAACTGCCCCAGCCC
TGGGGTTGCCAGATTTGACTAAGCCCTTTGAACTCTTTGTCGACGAGAAGCAGGGCTACGCCAAAGGTGTC
CTAACGCAAAAACTGGGACCTTGGCGTCGGCCGGTGGCCTACCTGTCCAAAAAGCTAGACCCAGTAGCAG
CTGGGTGGCCCCCTTGCCTACGGATGGTAGCAGCCATTGCCGTACTGACAAAGGATGCAGGCAAGCTAACC
ATGGGACAGCCACTAGTCATTCTGGCCCCCCATGCAGTAGAGGCACTAGTCAAACAACCCCCCGACCGCTG
GCTTTCCAACGCCCGGATGACTCACTATCAGGCCTTGCTTTTGGACACGGACCGGGTCCAGTTCGGACCG
GTGGTAGCCCTGAACCCGGCTACGCTGCTCCCACTGCCTGAGGAAGGGCTGCAACACAACTGCCTTGATAT
CCTGGCCGAAGCCCACGGAACCCGACCCGACCTAACGGACCAGCCGCTCCCAGACGCCGACCACACCTG
GTACACGGATGGAAGCAGTCTCTTACAAGAGGGACAGCGTAAGGCGGGAGCTGCGGTGACCACCGAGACC
GAGGTAATCTGGGCTAAAGCCCTGCCAGCCGGGACATCCGCTCAGCGGGCTGAACTGATAGCACTCACCC
AGGCCCTAAAGATGGCAGAAGGTAAGAAGCTAAATGTTTATACTGATAGCCGTTATGCTTTTGCTACTGCCCA
TATCCATGGAGAAATATACAGAAGGCGTGGGTTGCTCACATCAGAAGGCAAAGAGATCAAAAATAAAGACGA
GATCTTGGCCCTACTAAAAGCCCTCTTTCTGCCCAAAAGACTTAGCATAATCCATTGTCCAGGACATCAAAAG
GGACACAGCGCCGAGGCTAGAGGCAACCGGATGGCTGACCAAGCGGCCCGAAAGGCAGCCATCACAGAG
ACTCCAGACACCTCTACCCTCCTCATAGAAAATTCATCACCCTACACCTCAGAACATTTTCATTACACAGTGA
CTGATATAAAGGACCTAACCAAGTTGGGGGCCATTTATGATAAAACAAAGAAGTATTGGGTCTACCAAGGAAA
ACCTGTGATGCCTGACCAGTTTACTTTTGAATTATTAGACTTTCTTCATCAGCTGACTCACCTCAGCTTCTCAA
AAATGAAGGCTCTCCTAGAGAGAAGCCACAGTCCCTACTACATGCTGAACCGGGATCGAACACTCAAAAATA
TCACTGAGACCTGCAAAGCTTGTGCACAAGTCAACGCCAGCAAGTCTGCCGTTAAACAGGGAACTAGGGTC
CGCGGGCATCGGCCCGGCACTCATTGGGAGATCGATTTCACCGAGATAAAGCCCGGATTGTATGGCTATAAA
TATCTTCTAGTTTTTATAGATACCTTTTCTGGCTGGATAGAAGCCTTCCCAACCAAGAAAGAAACCGCCAAGG
TCGTAACCAAGAAGCTACTAGAGGAGATCTTCCCCAGGTTCGGCATGCCTCAGGTATTGGGAACTGACAATG
GGCCTGCCTTCGTCTCCAAGGTGAGTCAGACAGTGGCCGATCTGTTGGGGATTGATTGGAAATTACATTGT
GCATACAGACCCCAAAGCTCAGGCCAGGTAGAAAGAATGAATAGAACCATCAAGGAGACTTTAACTAAATTAA
CGCTTGCAACTGGCTCTAGAGACTGGGTGCTCCTACTCCCCTTAGCCCTGTACCGAGCCCGCAACACGCC
GGGCCCCCATGGCCTCACCCCATATGAGATCTTATATGGGGCACCCCCGCCCCTTGTAAACTTCCCTGACCC
TGACATGACAAGAGTTACTAACAGCCCCTCTCTCCAAGCTCACTTACAGGCTCTCTACTTAGTCCAGCACGA
AGTCTGGAGACCTCTGGCGGCAGCCTACCAAGAACAACTGGACCGACCGGTGGTACCTCACCCTTACCGA
GTCGGCGACACAGTGTGGGTCCGCCGACACCAGACTAAGAACCTAGAACCTCGCTGGAAAGGACCTTACA
CAGTCCTGCTGACCACCCCCACCGCCCTCAAAGTAGACGGCATCGCAGCTTGGATACACGCCGCCCACGT

GAAGGCTGCCGACCCCGGGGGTGGACCATCCTCTAGACTGACATGGCGCGTTCAACGCTCTCAAAACCCC
TTAAAAATAAGGTTGACGCGTCAACCCGCGAGGCCCCCTAATCCCCTTAATTCTTCTGATGCTCAGAGGGGT
CAGTATCGAATTCCTGCAGCCCCGCTGATCAGCCTCGACTGTGCCTTCTAG

Supplementary Sequence 6. Gag-COM-pol in v3b PE-eVLPs.

Key: Gag-linker-**COM**-linker-pol

ATGGGCCAGACTGTTACCACTCCCTTAAGTTTGACCTTAGGTCACTGGAAAGATGTCGAGCGGATCGCTCAC
AACCAGTCGGTAGATGTCAAGAAGAGACGTTGGGTTACCTTCTGCTCTGCAGAATGGCCAACCTTTAACGTC
GGATGGCCGCGAGACGGCACCTTTAACCGAGACCTCATCACCCAGGTTAAGATCAAGGTCTTTTCACCTGG
CCCGCATGGACACCCAGACCAGGTCCCCTACATCGTGACCTGGGAAGCCTTGGCTTTTGACCCCCCTCCCT
GGGTCAAGCCCTTTGTACACCCTAAGCCTCCGCCTCCTCTTCCTCCATCCGCCCCGTCTCTCCCCCTTGAA
CCTCCTCGTTCGACCCCGCCTCGATCCTCCCTTTATCCAGCCCTCACTCCTTCTCTAGGCGCCAAACCTAAA
CCTCAAGTTCTTTCTGACAGTGGGGGGCCGCTCATCGACCTACTTACAGAAGACCCCCCGCCTTATAGGGA
CCCAAGACCACCCCCTTCCGACAGGGACGGAAATGGTGGAGAAGCGACCCCTGCGGGAGAGGCACCGGA
CCCCTCCCCAATGGCATCTCGCCTACGTGGGAGACGGGAGCCCCCTGTGGCCGACTCCACTACCTCGCAG
GCATTCCCCCTCCGCGCAGGAGGAAACGGACAGCTTCAATACTGGCCGTTCTCCTCTTCTGACCTTTACAA
CTGGAAAAATAATAACCCTTCTTTTTCTGAAGATCCAGGTAAACTGACAGCTCTGATCGAGTCTGTCCTCATC
ACCCATCAGCCCACCTGGGACGACTGTCAGCAGCTGTTGGGGACTCTGCTGACCGGAGAAGAAAAACAAC
GGGTGCTCTTAGAGGCTAGAAAGGCGGTGCGGGGCGATGATGGGCGCCCCACTCAACTGCCCAATGAAGT
CGATGCCGCTTTTCCCCTCGAGCGCCCAGACTGGGATTACACCACCCAGGCAGGTAGGAACCACCTAGTC
CACTATCGCCAGTTGCTCCTAGCGGGTCTCCAAAACGCGGGCAGAAGCCCCACCAATTTGGCCAAGGTAAA
AGGAATAACACAAGGGCCCAATGAGTCTCCCTCGGCCTTCCTAGAGAGACTTAAGGAAGCCTATCGCAGGT
ACACTCCTTATGACCCTGAGGACCCAGGGCAAGAAACTAATGTGTCTATGTCTTTCATTTGGCAGTCTGCCC
CAGACATTGGGAGAAAGTTAGAGAGGTTAGAAGATTTAAAAAACAAGACGCTTGGAGATTTGGTTAGAGAGG
CAGAAAAGATCTTTAATAAACGAGAAACCCCGGAAGAAAGAGAGGAACGTATCAGGAGAGAAACAGAGGAA
AAAGAAGAACGCCGTAGGACAGAGGATGAGCAGAAAGAGAAAGAAAGAGATCGTAGGAGACATAGAGAGAT
GAGCAAGCTATTGGCCACTGTCGTTAGTGGACAGAAACAGGATAGACAGGGAGGAGAACGAAGGAGGTCC
CAACTCGATCGCGACCAGTGTGCCTACTGCAAAGAAAAGGGGCACTGGGCTAAAGATTGTCCCAAGAAACC
ACGAGGACCTCGGGGACCAAGACCCCAGACCTCCCTCCTGACCCTAGATGACTCCGGTGGCGGTGGATCA
GGGGGTGGTTCC**AAATCAATTCGCTGTAAAAACTGCAACAAACTGTTATTTAAGGCGGATTCCTTTGATCA**
CATTGAAATCAGGTGTCCGCGTTGCAAACGTCACATCATAATGCTGAATGCCTGCGAGCATCCCACGGAG
AAACATTGTGGGAAAAGAGAAAAAATCACGCATTCTGACGAAACCGTGCGTTATTCCGGAGGTGGAGGT
GGATAGGGAGGTCAGGGTCAGGAGCCCCCCCTGAACCCAGGATAACCCTCAAAGTCGGGGGGCAACCC
GTCACCTTCCTGGTAGATACTGGGGCCCAACACTCCGTGCTGACCCAAAATCCTGGACCCCTAAGTGATAA
GTCTGCCTGGGTCCAAGGGGCTACTGGAGGAAAGCGGTATCGCTGGACCACGGATCGCAAAGTACATCTA
GCTACCGGTAAGGTCACCCACTCTTTCCTCCATGTACCAGACTGTCCCTATCCTCTGTTAGGAAGAGATTTG
CTGACTAAACTAAAAGCCCAAATCCACTTTGAGGGATCAGGAGCTCAGGTTATGGACCAATGGGGCAGCC
CCTGCAAGTGTTGACCCTAAATATAGAAGATGAGTATCGGCTACATGAGACCTCAAAAGAGCCAGATGTTTCT
CTAGGGTCCACATGGCTGTCTGATTTTCCTCAGGCCTGGGCGGAAACCGGGGGCATGGGACTGGCAGTTC
GCCAAGCTCCTCTGATCATACCTCTGAAAGCAACCTCTACCCCCGTGTCCATAAAACAATACCCCATGTCACA
AGAAGCCAGACTGGGGATCAAGCCCCACATACAGAGACTGTTGGACCAGGGAATACTGGTACCCTGCCAGT

CCCCCTGGAACACGCCCCTGCTACCCGTTAAGAAACCAGGGACTAATGATTATAGGCCTGTCCAGGATCTGA
GAGAAGTCAACAAGCGGGTGGAAGACATCCACCCCACCGTGCCCAACCCTTACAACCTCTTGAGCGGGCT
CCCACCGTCCCACCAGTGGTACACTGTGCTTGATTTAAAGGATGCCTTTTTCTGCCTGAGACTCCACCCCAC
CAGTCAGCCTCTCTTCGCCTTTGAGTGGAGAGATCCAGAGATGGGAATCTCAGGACAATTGACCTGGACCA
GACTCCCACAGGGTTTCAAAAACAGTCCCACCCTGTTTGATGAGGCACTGCACAGAGACCTAGCAGACTTC
CGGATCCAGCACCCAGACTTGATCCTGCTACAGTACGTGGATGACTTACTGCTGGCCGCCACTTCTGAGCT
AGACTGCCAACAAGGTACTCGGGCCCTGTTACAAACCCTAGGGAACCTCGGGTATCGGGCCTCGGCCAAG
AAAGCCCAAATTTGCCAGAAACAGGTCAAGTATCTGGGGTATCTTCTAAAAGAGGGTCAGAGATGGCTGACT
GAGGCCAGAAAAGAGACTGTGATGGGGCAGCCTACTCCGAAGACCCCTCGACAACTAAGGGAGTTCCTAG
GGACGGCAGGCTTCTGTCGCCTCTGGATCCCTGGGTTTGCAGAAATGGCAGCCCCCTTGTACCCTCTCACC
AAAACGGGGACTCTGTTTAATTGGGGCCCAGACCAACAAAAGGCCTATCAAGAAATCAAGCAAGCTCTTCTA
ACTGCCCCAGCCCTGGGGTTGCCAGATTTGACTAAGCCCTTTGAACTCTTTGTCGACGAGAAGCAGGGCTA
CGCCAAAGGTGTCCTAACGCAAAAACTGGGACCTTGGCGTCGGCCGGTGGCCTACCTGTCCAAAAAGCTA
GACCCAGTAGCAGCTGGGTGGCCCCCTTGCCTACGGATGGTAGCAGCCATTGCCGTACTGACAAAGGATG
CAGGCAAGCTAACCATGGGACAGCCACTAGTCATTCTGGCCCCCCATGCAGTAGAGGCACTAGTCAAACAA
CCCCCCGACCGCTGGCTTTCCAACGCCCGGATGACTCACTATCAGGCCTTGCTTTTGGACACGGACCGGG
TCCAGTTCGGACCGGTGGTAGCCCTGAACCCGGCTACGCTGCTCCCACTGCCTGAGGAAGGGCTGCAACA
CAACTGCCTTGATATCCTGGCCGAAGCCCACGGAACCCGACCCGACCTAACGGACCAGCCGCTCCCAGAC
GCCGACCACACCTGGTACACGGATGGAAGCAGTCTCTTACAAGAGGGACAGCGTAAGGCGGGAGCTGCGG
TGACCACCGAGACCGAGGTAATCTGGGCTAAAGCCCTGCCAGCCGGGACATCCGCTCAGCGGGCTGAACT
GATAGCACTCACCCAGGCCCTAAAGATGGCAGAAGGTAAGAAGCTAAATGTTTATACTGATAGCCGTTATGCT
TTTGCTACTGCCCATATCCATGGAGAAATATACAGAAGGCGTGGGTTGCTCACATCAGAAGGCAAAGAGATC
AAAAATAAAGACGAGATCTTGGCCCTACTAAAAGCCCTCTTTCTGCCCAAAAGACTTAGCATAATCCATTGTC
CAGGACATCAAAAGGGACACAGCGCCGAGGCTAGAGGCAACCGGATGGCTGACCAAGCGGCCCGAAAGG
CAGCCATCACAGAGACTCCAGACACCTCTACCCTCCTCATAGAAAATTCATCACCCTACACCTCAGAACATTT
TCATTACACAGTGACTGATATAAAGGACCTAACCAAGTTGGGGGCCATTTATGATAAAACAAAGAAGTATTGG
GTCTACCAAGGAAAACCTGTGATGCCTGACCAGTTTACTTTTGAATTATTAGACTTTCTTCATCAGCTGACTCA
CCTCAGCTTCTCAAAAATGAAGGCTCTCCTAGAGAGAAGCCACAGTCCCTACTACATGCTGAACCGGGATCG
AACACTCAAAAATATCACTGAGACCTGCAAAGCTTGTGCACAAGTCAACGCCAGCAAGTCTGCCGTTAAACA
GGGAACTAGGGTCCGCGGGCATCGGCCCGGCACTCATTGGGAGATCGATTTCACCGAGATAAAGCCCGGA
TTGTATGGCTATAAATATCTTCTAGTTTTTATAGATACCTTTTCTGGCTGGATAGAAGCCTTCCCAACCAAGAAA
GAAACCGCCAAGGTCGTAACCAAGAAGCTACTAGAGGAGATCTTCCCCAGGTTCGGCATGCCTCAGGTATT
GGGAACTGACAATGGGCCTGCCTTCGTCTCCAAGGTGAGTCAGACAGTGGCCGATCTGTTGGGGATTGATT
GGAAATTACATTGTGCATACAGACCCCAAAGCTCAGGCCAGGTAGAAAGAATGAATAGAACCATCAAGGAGA
CTTTAACTAAATTAACGCTTGCAACTGGCTCTAGAGACTGGGTGCTCCTACTCCCCTTAGCCCTGTACCGAG
CCCGCAACACGCCGGGCCCCCATGGCCTCACCCCATATGAGATCTTATATGGGGCACCCCCGCCCCTTGTA
AACTTCCCTGACCCTGACATGACAAGAGTTACTAACAGCCCCTCTCTCCAAGCTCACTTACAGGCTCTCTAC
TTAGTCCAGCACGAAGTCTGGAGACCTCTGGCGGCAGCCTACCAAGAACAACTGGACCGACCGGTGGTAC
CTCACCCTTACCGAGTCGGCGACACAGTGTGGGTCCGCCGACACCAGACTAAGAACCTAGAACCTCGCTG

GAAAGGACCTTACACAGTCCTGCTGACCACCCCCACCGCCCTCAAAGTAGACGGCATCGCAGCTTGGATAC
ACGCCGCCCACGTGAAGGCTGCCGACCCCGGGGGTGGACCATCCTCTAGACTGACATGGCGCGTTCAAC
GCTCTCAAAACCCCTTAAAAATAAGGTTGACGCGTCAACCCGCGAGGCCCCCTAATCCCCTTAATTCTTCTG
ATGCTCAGAGGGGTCAGTATCGAATTCCTGCAGCCCCGCTGATCAGCCTCGACTGTGCCTTCTAG

Supplementary References

1 Nelson, J. W. *et al.* Engineered pegRNAs improve prime editing efficiency. *Nat Biotechnol* **40**, 402-410 (2022). https://doi.org:10.1038/s41587-021-01039-7
