# Supplementary material for: Engineered virus-like particles for transient delivery of prime editor ribonucleoprotein complexes in vivo
Source: Nat Biotechnol. 2024 Jan 8;42(10):1526–37. doi: 10.1038/s41587-023-02078-y (PMC11228131; doi:10.1038/s41587-023-02078-y)
Supplement: Supplementary file 2 — Reporting Summary [file 41587_2023_2078_MOESM2_ESM.pdf]

nature portfolio

Corresponding author(s): David R. Liu

Last updated by author(s): Nov 16, 2023

Reporting Summary

Nature Portfolio wishes to improve the reproducibility of the work that we publish. This form provides structure for consistency and transparency in reporting. For further information on Nature Portfolio policies, see our Editorial Policies and the Editorial Policy Checklist.

Statistics

For all statistical analyses, confirm that the following items are present in the figure legend, table legend, main text, or Methods section.

n/a	Confirmed	
☐	☒	The exact sample size (*n*) for each experimental group/condition, given as a discrete number and unit of measurement
☐	☒	A statement on whether measurements were taken from distinct samples or whether the same sample was measured repeatedly
☒	☐	The statistical test(s) used AND whether they are one- or two-sided<br>*Only common tests should be described solely by name; describe more complex techniques in the Methods section.*
☒	☐	A description of all covariates tested
☒	☐	A description of any assumptions or corrections, such as tests of normality and adjustment for multiple comparisons
☐	☒	A full description of the statistical parameters including central tendency (e.g. means) or other basic estimates (e.g. regression coefficient) AND variation (e.g. standard deviation) or associated estimates of uncertainty (e.g. confidence intervals)
☒	☐	For null hypothesis testing, the test statistic (e.g. *F*, *t*, *r*) with confidence intervals, effect sizes, degrees of freedom and *P* value noted<br>*Give P values as exact values whenever suitable.*
☒	☐	For Bayesian analysis, information on the choice of priors and Markov chain Monte Carlo settings
☒	☐	For hierarchical and complex designs, identification of the appropriate level for tests and full reporting of outcomes
☒	☐	Estimates of effect sizes (e.g. Cohen's *d*, Pearson's *r*), indicating how they were calculated

Our web collection on statistics for biologists contains articles on many of the points above.

Software and code

Policy information about availability of computer code

Data collection	Miseq reporter software (v2.6) was used on the Illumina Miseq to demultiplex HTS data. Sony MA900 sorter was used for FACS with MA900 Cell Sorter software v3.1. ERGs were recorded with the Celeris rodent electrophysiology system.
Data analysis	Frequency, mean, and standard deviations were calculated using GraphPad Prism 9. CRISPResso2 was used to analyze HTS data for quantifying editing efficiency at the genomic sites (https://github.com/pinellolab/CRISPResso2). ERGs were analyzed with Espion V6 software. CIRCLE-seq analysis was performed using open-source CIRCLE-seq analysis software and default recommended parameters (https://github.com/tsailabSJ/circleseq).

For manuscripts utilizing custom algorithms or software that are central to the research but not yet described in published literature, software must be made available to editors and reviewers. We strongly encourage code deposition in a community repository (e.g. GitHub). See the Nature Portfolio guidelines for submitting code & software for further information.

Data

Policy information about availability of data

All manuscripts must include a data availability statement. This statement should provide the following information, where applicable:

- Accession codes, unique identifiers, or web links for publicly available datasets
- A description of any restrictions on data availability
- For clinical datasets or third party data, please ensure that the statement adheres to our policy

High-throughput sequencing data files are available from NCBI SRA under accession code PRJNA980181. DNA sequences of the PE-eVLP architectures are provided in the Supplementary Information. Key plasmids from this work are available from Addgene and other plasmids are available from the corresponding author on request.

Research involving human participants, their data, or biological material

Policy information about studies with human participants or human data. See also policy information about sex, gender (identity/presentation), and sexual orientation and race, ethnicity and racism.

Reporting on sex and gender	This study does not involve human research participants.
Reporting on race, ethnicity, or other socially relevant groupings	N/A
Population characteristics	N/A
Recruitment	N/A
Ethics oversight	N/A

Note that full information on the approval of the study protocol must also be provided in the manuscript.

Field-specific reporting

Please select the one below that is the best fit for your research. If you are not sure, read the appropriate sections before making your selection.

☒ Life sciences ☐ Behavioural & social sciences ☐ Ecological, evolutionary & environmental sciences

For a reference copy of the document with all sections, see nature.com/documents/nr-reporting-summary-flat.pdf

Life sciences study design

All studies must disclose on these points even when the disclosure is negative.

Sample size	Sample sizes were n=3 independent biological replicates, in accordance with previous literature and standards in the field of genome editing technologies (Anzalone 2019, Levy 2020, Banskota and Raguram 2022)
Data exclusions	For the rd6 and rd12 model correction, mice were co-injected with PE-eVLPs and AAV1-GFP. GFP signal was examined as a marker for successful injection. Samples from the eyes that showed less than 80% GFP signal was excluded from the analysis due to inefficient injection. No other data were excluded from the study.
Replication	All experiments were repeated more than once. All attempts at replication were successful.
Randomization	All control and test conditions were assigned randomly. For example, for all in vitro/tissue culture experiments, conditions were assigned randomly to wells across 96- or 48-well plates and plate positioning is not expected to affect experimental outcome. For in vivo experiments, mice were assigned randomly into groups.
Blinding	All HTS data were analyzed using an automated CRISPResso2 script that does not allow experimenter intervention, therefore experimenter was not blinded. Subretinal injection editing data were analyzed by blinded investigator. Blinding was not necessary for other experiments because control and test conditions were processed identically.

Reporting for specific materials, systems and methods

We require information from authors about some types of materials, experimental systems and methods used in many studies. Here, indicate whether each material, system or method listed is relevant to your study. If you are not sure if a list item applies to your research, read the appropriate section before selecting a response.

Materials & experimental systems

n/a	Involved in the study	
☐	☒	Antibodies
☐	☒	Eukaryotic cell lines
☒	☐	Palaeontology and archaeology
☐	☒	Animals and other organisms
☒	☐	Clinical data
☒	☐	Dual use research of concern
☒	☐	Plants

Methods

n/a	Involved in the study	
☒	☐	ChIP-seq
☐	☒	Flow cytometry
☒	☐	MRI-based neuroimaging

Antibodies

Antibodies used	Mouse Cas9 antibody (Thermo Fisher Scientific, #MA5-23519); Rabbit GAPDH antibody (Cell signaling Technology, #2118); Goat anti-mouse MFRP antibody (R&D Systems #AF3445); Mouse anti-mouse RPE65 antibody (in house production; obtained from the laboratory of Dr. Krzysztof Palczewski, UC Irvine ; Golczak et al, 2010); Rabbit anti-beta-actin polyclonal antibody (Cell Signaling Technology; 4970S); Anti-NeuN antibody (Abcam, ab190565); Goat anti-mouse secondary antibody (LI-COR IRDye 680RD, 926-68070); Goat anti-rabbit antibody (LI-COR IRDye 800RD, 926-32211); Donkey anti-goat IgG-HRP antibody (Abcam, ab97110); Goat anti-mouse IgG-HRP antibody (Cell Signaling Technology, 7076S); Goat anti-rabbit IgG-HRP antibody (Cell signaling Technology, 7074S); Rabbit anti-ZO-1 polyclonal antibody (Invitrogen, #61-7300); Alexa Fluor 594-conjugated donkey anti-rabbit IgG (Thermo Fisher; A21207); Alexa Fluor 647-conjugated donkey anti-goat IgG (Thermo Fisher, A32849);
Validation	All commercial antibodies were validated by the manufacturers for western blot. For in-house produced antibody, we confirmed clear bands at the expected molecular weight with positive control included in the experiments.

Eukaryotic cell lines

Policy information about cell lines and Sex and Gender in Research

Cell line source(s)	HEK293T, Neuro-2A and HEK293T/17 cells were obtained from ATCC. Gesicle 293T cells were obtained from Takara Bio. NIH 3T3 cell containing lentivirally integrated Rpe65 gene containing mutation was a gift from Palczewski Lab.
Authentication	Commercial cell lines were authenticated by the supplier using STR analysis. The gifted cell line was not authenticated.
Mycoplasma contamination	All cell lines were tested negative for mycoplasma.
Commonly misidentified lines (See ICLAC register)	None used.

Animals and other research organisms

Policy information about studies involving animals; ARRIVE guidelines recommended for reporting animal research, and Sex and Gender in Research

Laboratory animals	Time pregnant C57BL/6J mice (027) for P0 studies were purchased from Charles River Laboratories. Age of the pregnant mice was not specified. Litters were used for injection on the day of birth. Reitnal degenration mice models rd6 (003684) and rd12 (005379) were purchased from the Jackson Laboratory. Subretinal injections were performed on 5-week-old rd6 and rd12 mice. Mouse housing facilities were maintained on a 12 h light/12 h dark cycle, with stable temperature (70 degree Fahrenheit +/- 2 degree Fahrenheit) and humidity (40% +/- 10%), with ad libitum access to standard rodent diet and water.
Wild animals	This study does not involve wild animals.
Reporting on sex	Both male and female mice were used for each condition.
Field-collected samples	This study does not involve samples collected from the field.
Ethics oversight	Broad Institute IACUC committee (D16-00903; 0048-04-15-2) and University of California, Irvine IACUC committee (D16-00259; AUP-21-096) provided ethics overnight for all experiments involving live animals.

Note that full information on the approval of the study protocol must also be provided in the manuscript.

Plants

Seed stocks	N/A
Novel plant genotypes	N/A
Authentication	N/A

Flow Cytometry

Plots

Confirm that:

☒ The axis labels state the marker and fluorochrome used (e.g. CD4-FITC).

☒ The axis scales are clearly visible. Include numbers along axes only for bottom left plot of group (a 'group' is an analysis of identical markers).

☒ All plots are contour plots with outliers or pseudocolor plots.

☒ A numerical value for number of cells or percentage (with statistics) is provided.

Methodology

Sample preparation	Nuclei extraction protocol for FACS is described in the methods section of this manuscript.
Instrument	Sony MA900 Cell Sorter
Software	MA900 Cell Sorter software v3.1
Cell population abundance	On average 2.9% of sorted nuclei were GFP-positive.
Gating strategy	Single nuclei were gated based on forward scatter (FSC-A) and back scatter (BSC-A) ratios, and DyeCycle Ruby or DAPI signal. GFP-positive nuclei were gated based on FITC signal. Representative gating strategy is presented in Extended Data Fig. 8.

☒ Tick this box to confirm that a figure exemplifying the gating strategy is provided in the Supplementary Information.
