# Supplementary figures and images for: Engineered virus-like particles for transient delivery of prime editor ribonucleoprotein complexes in vivo
Source: Nat Biotechnol. 2024 Jan 8;42(10):1526–37. doi: 10.1038/s41587-023-02078-y (PMC11228131; doi:10.1038/s41587-023-02078-y)

anti-MFRP

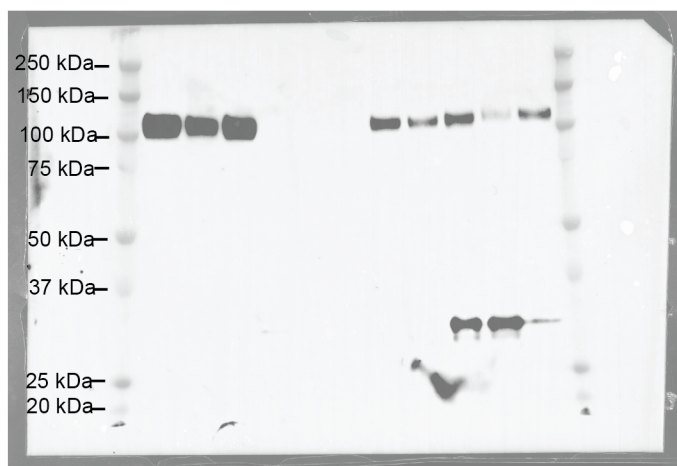

anti- $\beta$ -actin

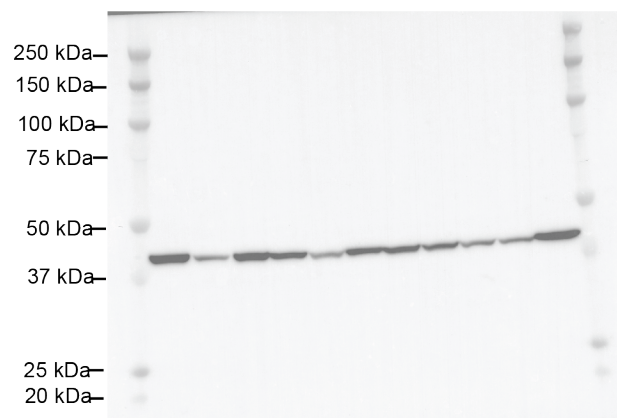

anti-RPE65

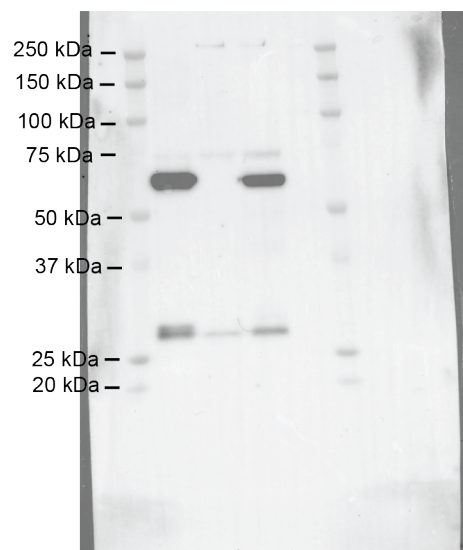

anti- $\beta$ -actin

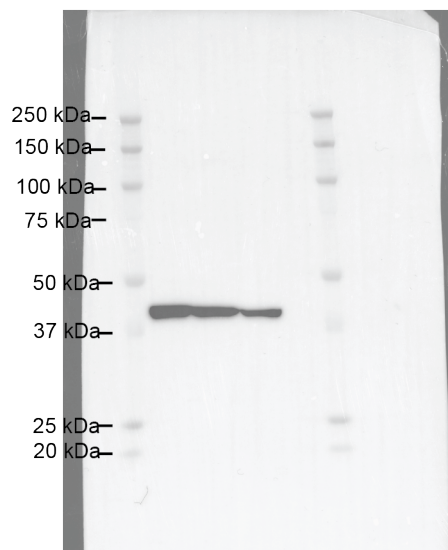

Supplement: Supplementary file 4 — Unprocessed western blots for Fig. 5d,k. [file 41587_2023_2078_MOESM4_ESM.pdf]
